# An integrative systematic framework helps to reconstruct skeletal evolution of glass sponges (Porifera, Hexactinellida)

**DOI:** 10.1186/s12983-017-0191-3

**Published:** 2017-03-21

**Authors:** Martin Dohrmann, Christopher Kelley, Michelle Kelly, Andrzej Pisera, John N. A. Hooper, Henry M. Reiswig

**Affiliations:** 1 Department of Earth & Environmental Sciences, Palaeontology & Geobiology, Molecular Geo- & Palaeobiology Lab, Ludwig-Maximilians-University Munich, Richard-Wagner-Str. 10, 80333 Munich, Germany; 2 Hawaii Undersea Research Laboratory, University of Hawaii at Manoa, 1000 Pope Rd, MSB 229, Honolulu, 96822 HI USA; 3 Coasts and Oceans National Centre, National Institute of Water and Atmospheric Research (NIWA) Ltd, Private Bag 99940, Newmarket, Auckland, 1149 New Zealand; 4 Institute of Paleobiology, Polish Academy of Sciences, ul. Twarda 51/55, 00-818 Warszawa, Poland; 5 Biodiversity & Geosciences Program, Queensland Museum, South Brisbane, QLD 4101 Australia; 6 Eskitis Institute for Drug Discovery, Griffith University, Nathan, QLD 4111 Australia; 7 Natural History Section, Royal British Columbia Museum, 675 Belleville Street, Victoria, BC V8W 9W2 Canada; 8 Department of Biology, University of Victoria, 3800 Finnerty Road, Victoria, BC V8P 4H9 Canada

**Keywords:** Ancestral state reconstruction, Character evolution, Classification, Hexactinellida, Integrative systematics, Phylogeny, Porifera, Total evidence

## Abstract

**Background:**

Glass sponges (Class Hexactinellida) are important components of deep-sea ecosystems and are of interest from geological and materials science perspectives. The reconstruction of their phylogeny with molecular data has only recently begun and shows a better agreement with morphology-based systematics than is typical for other sponge groups, likely because of a greater number of informative morphological characters. However, inconsistencies remain that have far-reaching implications for hypotheses about the evolution of their major skeletal construction types (body plans). Furthermore, less than half of all described extant genera have been sampled for molecular systematics, and several taxa important for understanding skeletal evolution are still missing. Increased taxon sampling for molecular phylogenetics of this group is therefore urgently needed. However, due to their remote habitat and often poorly preserved museum material, sequencing all 126 currently recognized extant genera will be difficult to achieve. Utilizing morphological data to incorporate unsequenced taxa into an integrative systematics framework therefore holds great promise, but it is unclear which methodological approach best suits this task.

**Results:**

Here, we increase the taxon sampling of four previously established molecular markers (18S, 28S, and 16S ribosomal DNA, as well as cytochrome oxidase subunit I) by 12 genera, for the first time including representatives of the order Aulocalycoida and the type genus of Dactylocalycidae, taxa that are key to understanding hexactinellid body plan evolution. Phylogenetic analyses suggest that Aulocalycoida is diphyletic and provide further support for the paraphyly of order Hexactinosida; hence these orders are abolished from the Linnean classification. We further assembled morphological character matrices to integrate so far unsequenced genera into phylogenetic analyses in maximum parsimony (MP), maximum likelihood (ML), Bayesian, and morphology-based binning frameworks. We find that of these four approaches, total-evidence analysis using MP gave the most plausible results concerning congruence with existing phylogenetic and taxonomic hypotheses, whereas the other methods, especially ML and binning, performed more poorly. We use our total-evidence phylogeny of all extant glass sponge genera for ancestral state reconstruction of morphological characters in MP and ML frameworks, gaining new insights into the evolution of major hexactinellid body plans and other characters such as different spicule types.

**Conclusions:**

Our study demonstrates how a comprehensive, albeit in some parts provisional, phylogeny of a larger taxon can be achieved with an integrative approach utilizing molecular and morphological data, and how this can be used as a basis for understanding phenotypic evolution. The datasets and associated trees presented here are intended as a resource and starting point for future work on glass sponge evolution.

**Electronic supplementary material:**

The online version of this article (doi:10.1186/s12983-017-0191-3) contains supplementary material, which is available to authorized users.

## Background

**Fig. 1:**
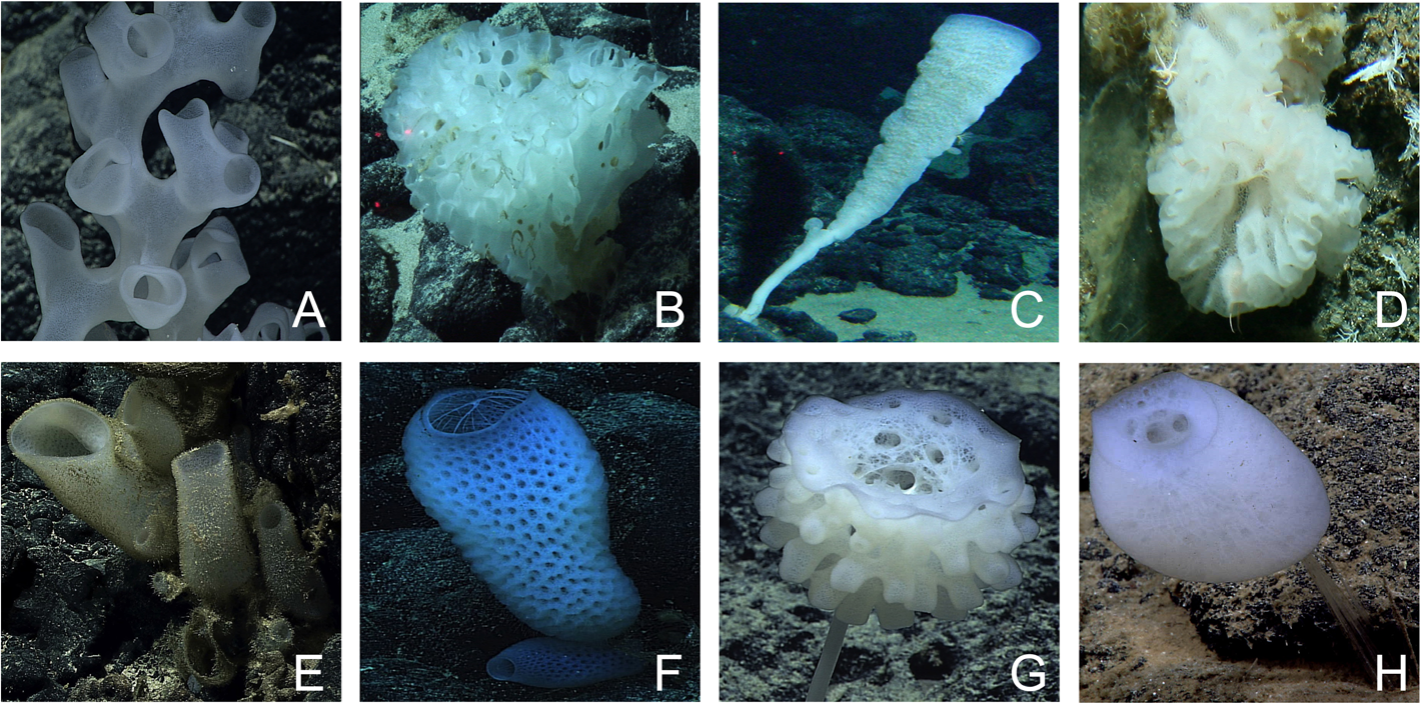
Some examples of glass sponges (Porifera: Hexactinellida). **a**-**c**, **e**-**h** from off Hawaii (images **a** and **e**-**h** captured by the *Deep Discoverer* ROV onboard the NOAA ship *Okeanos Explorer*, courtesy of NOAA OER; images **b**-**c** captured by the *Pisces 4 and 5* submersibles onboard the R/V *Kaimikai-o-Kanaloa*, courtesy of HURL); **d** from off New Zealand, Chatham Rise (image captured by DTIS [Deep Towed Imaging System] onboard RV Tangaroa, courtesy of NIWA). **a**-**d** examples of dictyonal sponges, **e**-**h** examples of lyssacine sponges (see text). **a**-**g** subclass Hexasterophora, **h** subclass Amphidiscophora. **a** *Farrea occa* (Farreidae), specimen 10–30 cm high, 2026 m depth. **b** *Heterorete* sp. (Euretidae), specimen 30–50 cm (?) diameter, 1559 m depth. **c** *Tretopleura* sp. (Uncinateridae), specimen 51 cm high, 888 m depth. **d** *Aulocalyx australis* (Aulocalycidae), specimen ~6 cm in diameter, 770–919 m depth. **e** *Lophocalyx* sp. (Rossellidae Lanuginellinae), specimens 10–50 cm high, depth 2247 m. **f** *Regadrella* sp. (Euplectellidae Corbitellinae, showing the iconical “venus-flower basket” body shape), specimen 5–30 cm high, 2132 m depth. **g** *Saccocalyx* sp. (Euplectellidae Bolosominae; note fleshy stalk below main body), specimen 30–50 cm (?) high, 1557 m depth. **h** *Hyalonema* sp. (Hyalonematidae; note stalk of naked anchor spicules below main body), specimen 5–10 cm high, 4824 m depth

**Fig. 2:**
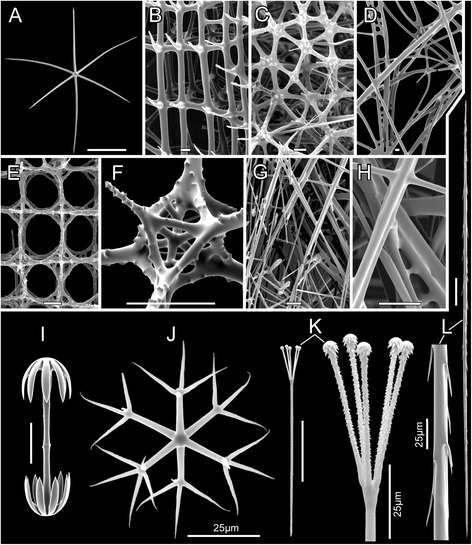
Hexactinellid framework and basic spicule types. Scanning Electron Micrographs (SEM). **a** hexactine megasclere (*Dictyocalyx* sp., Euplectellidae Corbitellinae), the eponymous character for the class. Other megasclere (structural spicule) types are derived from hexactins by reduction of rays. **b** dictyonal framework of the “regular” (euretoid) type (*Conorete gordoni*, Euretidae), as found in most sceptrulophorans. **c** dictyonal framework with “haphazard” connection of hexactins (*Dactylocalyx pumiceus*), as found in Dactylocalycidae. **d** dictyonal framework of the aulocalycoid type (*Aulocalyx australis*), as found in Aulocalycidae. Note that in addition to proper ray fusion (as in b, c, and e; see [2] and Additional file 4 for details), synapticular fusion – cementation of spicules by siliceous bridges – is also common in this type of framework construction. **e** dictyonal framework of the Lychniscosida (*Neoaulocystis zitelli*, Aulocystidae; facial view). Instead of regular hexactins, lantern-like spicules (lychniscs) are the building-blocks of the frameworks in this paleontologically important relict-group. **f** oblique view of surface lychnisc of *N. zitteli*. **g** lyssacine construction type of parenchymal skeleton (*Atlantisella* sp., Euplectellidae Corbitellinae), as found in Lyssacinosida and Amphidiscophora. In this type of body plan, spicules either do not fuse at all, or (often older) parts of the skeleton fuse by synapticular bridging (only in Lyssacinosida); proper ray fusion as in the dictyonal body plan never occurs. **h** detail of g, showing synapticular bridging (*lower right*). **i** amphidisc (*Hyalonema populiferum*, Hyalonematidae), the diagnostic microsclere and defining autapomorphy of subclass Amphidiscophora. **j** hexaster (*Farrea omniclavata*, Farreidae), the diagnostic microsclere and defining autapomorphy of subclass Hexasterophora. The pictured spicule is an oxyhexaster, meaning that the secondary ray tips are pointed (without ornamentation); for further examples of hexasters see Fig. 3. **k** septrule (*C. gordoni*), the diagnostic spicule type and defining autapomorphy of Sceptrulophora. The pictured spicule is a scopule; for further examples of sceptrules and discussion of their evolution, see [19]. **l** unicate (*F. omniclavata*), a spicule type found in most species of Sceptrulophora and Amphidiscophora. All *scale bars* without lettering = 100 μm

**Fig. 3:**
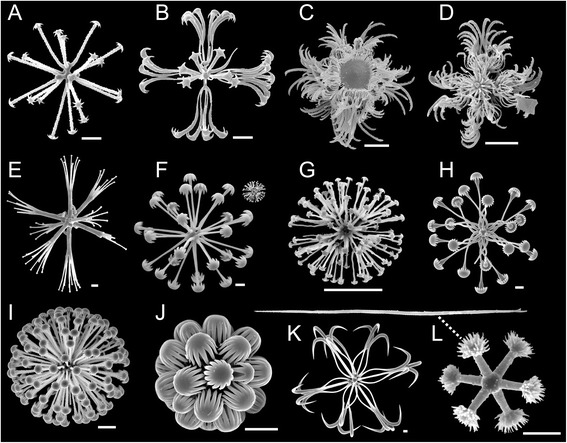
Some examples of hexasters. SEM. **a** spherical discohexaster with few terminal rays (*Hyalascus* sp., Rossellidae Rossellinae). **b** floricome (*Regadrella* sp., Euplectellidae Corbitellinae). **c** discoplumicome (*Saccocalyx pedunculatus*, Euplectellidae Bolosominae). **d** strobiloplumicome (*Doconesthes dustinchiversi*, Rossellidae Lanuginellinae). **e** discoctaster (*Acanthascus malacus*, Rossellidae Acanthascinae). **f** comparison of macrodiscohexaster (*Amphidiscella lecus*, Euplectellidae Bolosominae) and microdiscohexaster (*Schaudinnia* sp., Rossellidae Rossellinae) *upper right* at same scale. **g** microdiscohexaster, enlarged (*Schaudinnia* sp.). **h** spirodiscohexaster (*Saccocalyx pedunculatus*, Euplectellidae Bolosominae). **i** discaster (*Walteria flemmingi*, Euplectellidae Corbitellinae). **j** spherical discohexaster with large anchorate discs (*Rhabdopectella tintinnus*, Euplectellidae Bolosominae). **k** drepanocome (*Amphidiscella* sp.). **l** graphiocome, center and terminal ray (*Regadrella* sp., Euplectellidae Corbitellinae). All *scale bars* = 10 μm

Glass sponges (Hexactinellida; Fig. 1) constitute one of the four classes of Porifera, being distinguished from the other three classes (Demospongiae, Homoscleromorpha, and Calcarea) by having siliceous skeletal elements (spicules) with triaxonic symmetry (i.e., six-rayed spicules [hexactins] and their derivatives with reduced rays; Fig. 2a) and a largely syncytial soft tissue organization [1, 2]. Within Porifera, they are most closely related to Demospongiae [3, 4] and their monophyly is strongly supported by both morphological and molecular data [3]. Although in terms of known extant diversity they represent a relatively minor group (625 valid species as of May 2016 [5]), glass sponges are of great importance for the ecology of the deep-sea benthos (the habitat they are mostly restricted to) and are geologically relevant as they contributed to the formation of massive reefs, especially in the Mesozoic, which are still preserved as rock formations throughout Europe (e.g., [2, 6–9]). Furthermore, their spicules have remarkable physical properties, which make them highly interesting study objects for materials scientists (e.g., [10–12]). Glass sponges can be aesthetically appealing in terms of their unusual morphology and astonishing variety of spicule forms [2] (Figs. 1, 2 and 3). The high diversity and complexity of morphological features of hexactinellids provide ample characters for morphology-based systematics, and as a result there is relatively good agreement between molecular phylogenies and taxonomy in comparison to other sponge groups [3, 13, 14]. For example, monophyly is supported by molecular data for all except one of the families sampled so far for more than one genus, as well as for almost all genera sampled so far for more than one species [3, 15, 16] (the only exceptions were Euretidae, a clear “waste-bin” taxon [17], *Rossella*, which has subsequently been split into two separate genera [18], and *Aphrocallistes* and *Heterochone*, whose reciprocal monophyly might be difficult to reconstruct due to gene-tree species-tree conflicts [19]).

The division of Hexactinellida into the two subclasses Amphidiscophora and Hexasterophora [20, 21] is well supported by the mutually exclusive occurrence of amphidiscs and hexasters in these groups (Fig. 2i, j), and is also highly corroborated by molecular data [3]. Amphidiscophora contains a single extant order, Amphidiscosida (Hyalonematidae, Pheronematidae, Monorhaphididae). Hexasterophora currently comprises four orders: Hexactinosida (Aphrocallistidae, Auloplacidae, Craticulariidae, Cribrospongiidae, Dactylocalycidae, Euretidae, Farreidae, Fieldingiidae, Tretodictyidae), Aulocalycoida (Aulocalycidae, Uncinateridae), Lychniscosida (Aulocystidae, Diapleuridae), and Lyssacinosida (Euplectellidae, Leucopsacidae, Rossellidae). Members of the first three orders have so-called dictyonal frameworks, which are rigid internal skeletons composed of fused hexactins, whereas members of Lyssacinosida have internal skeletons composed of mostly unfused spicules, a condition called lyssacine that is also characteristic of Amphidiscophora (Fig. 2b-h). Molecular phylogenetic analyses based on ribosomal DNA (rDNA) and cytochrome oxidase subunit I (COI) sequences have supported monophyly of Lyssacinosida [15, 16] but have found that Dactylocalycidae is more closely related to that order than to the remaining hexactinosidans (= Sceptrulophora; a clade well-supported by possession of sceptrules and uncinates [Fig. 2k, l]), rendering Hexactinosida paraphyletic [3, 15, 16]. This suggests that dictyonal skeletons could have either evolved independently in Sceptrulophora and Dactylocalycidae, or alternatively that the lyssacine body plan “re-evolved” in Lyssacinosida [15]. However, a more comprehensive phylogeny, especially including Aulocalycoida and Lychniscosida, is necessary to understand the evolution of the dictyonal and lyssacine body plans. Understanding the evolution of other aspects of the glass sponge skeleton, such as the myriad different types of hexasters (see Fig. 3 for a few examples), would also greatly benefit from a phylogeny including as many genera as possible.

Prior to the present study, sequence coverage of genera was only ~36%. Although we here increase this number to ~45%, this is still low, and given the difficulties of obtaining suitable material from targeted taxa of this deep-sea group, is not likely to increase substantially in the near future. Therefore, utilization of morphological character data to integrate unsequenced genera into a phylogenetic analysis framework holds some promise to obtain a, albeit somewhat provisional, comprehensive phylogeny of Hexactinellida. In this study, we have increased molecular taxon sampling of the four markers established by Dohrmann et al. [3, 16] by 12 additional genera, which include representatives of one additional order (Aulocalycoida), and three additional families (Aulocalycidae, Craticulariidae, Uncinateridae). Furthermore, we compiled morphological character matrices from all described extant genera of the two subclasses. Molecular and morphological datasets were first analyzed separately and then combined to incorporate the unsequenced genera into the molecular phylogeny by using “total-evidence” approaches in maximum-parsimony (MP), maximum-likelihood (ML), and Bayesian analysis frameworks, as well as a “morphology-based phylogenetic binning” approach recently developed by Berger and Stamatakis [22]. Comparisons of the results of these four methods revealed that MP yielded trees in better congruence with previous taxonomic and phylogenetic hypotheses than the other methods, at least for Hexasterophora. We then used this comprehensive, total-evidence phylogeny to investigate hexactinellid skeletal evolution by means of ancestral state reconstruction in MP and ML frameworks. Based on our phylogenetic results, we also propose several changes to the current higher-level Linnean classification.

## Methods

### Morphological data assembly and phylogenetic analysis

Analyses of morphological data were performed on genus-level, i.e., monophyly of all genera was assumed a priori. Although a simplifying assumption, it is reasonable because most hexactinellid genera are morphologically well delineated. Furthermore, almost half of them (59/126 = 46.8%; counted May 2016) are monospecific (see Appendix 1). In any case, a comprehensive analysis on species-level would have been too time-consuming and is left for future projects.

A genus-level matrix comprising 105 taxa and 154 morphological characters was recently compiled by Henkel et al. [23] to reconstruct hexactinellid phylogeny, with a focus on Hexasterophora. This matrix was kindly provided to us by Daniela Henkel and used as a starting point for building our own morphological datasets. Upon inspection of this matrix, we noticed several errors and also that taxonomic literature published after the 2002 book *Systema Porifera* [1] was not taken into consideration by these authors. Thus, in order to incorporate post-2002 taxonomic work and correct errors, we re-checked every entry against the relevant literature [1, 17–19, 24–55], as well as some personal observations. We also excluded some uninformative or in our opinion not useful characters, while including others that we deemed informative. Our coding philosophy followed Dohrmann et al. [3], i.e., we used hierarchical, presence-absence (0/1) coding of inferred ground states, thereby avoiding an excess of character states, missing data, and polymorphisms. Matrix editing was performed in Mesquite 2.75 [56].

We also added eight genera that were described or resurrected after 2002 (*Acanthascus, Asceptrulum, Dictyoplax, Homoieurete, Indiella, Nodastrella*, *Pinulasma*, *Staurocalyptus*), eight genera of the subclass Amphidiscophora (*Chalaronema*, *Compsocalyx*, *Lophophysema*, *Platylistrum*, *Poliopogon*, *Schulzeviella*, *Sericolophus, Tabachnickia*), three genera that were not included by Henkel et al. [23] for unknown reasons (*Clathrochone*, *Hyaloplacoida, Ijimaiella*), and two so far undescribed genera for which DNA sequence data are available: Rossellinae n. gen. from New Zealand (Reiswig & Kelly, in prep.) and Bolosominae n. gen. from Hawaii (MD, unpubl. obs.). For rooting purposes, we also included an “artificial” outgroup taxon with all characters coded as “0” – using an actual non-hexactinellid sponge genus as an outgroup would have been useless since most characters used here are not comparable to characters of other sponge classes. The only taxa we did not include are three very poorly known genera (*Deanea, Diaretula, Hyalocaulus*), which would have mostly introduced missing data without contributing much information. A taxonomic overview of the included genera is given in Appendix 1.

Initial phylogenetic analysis of this matrix yielded quite unsatisfactory results (not shown), as many groups whose monophyly seems highly plausible were not recovered, and conversely some clades emerged that cannot reasonably be accepted as real. We therefore excluded some characters suspected to be overly homoplastic, included additional characters that we hoped might be informative, and/or recoded certain characters. Finally, we decided to construct separate matrices for the two subclasses. This was necessary because, although monophyly of Amphidiscophora was always recovered, this clade consistently nested within Hexasterophora – apparently because it shares some important characters with the hexasterophoran family Rossellidae (Lyssacinosida). Although these similarities are striking, it is highly unlikely that they represent synapomorphies of the two taxa – they are better interpreted as symplesiomorphies or convergences, because the two subclasses are highly supported as being reciprocally monophyletic by other characters and molecular data (see Introduction). The final Amphidiscophora matrix has 13 taxa and 29 characters, and the final Hexasterophora matrix has 114 taxa and 108 characters; these datasets can be found in Nexus format in Additional files 1 and 2 and as tables with annotated character lists in Additional files 3 and 4 (all Additional files are available at figshare [https://doi.org/10.6084/m9.figshare.3120130.v3]).

Phylogenetic analysis of the morphological data matrices was performed under maximum parsimony (MP) as implemented in TNT v1.1 [57], using “new technology” searches (sectorial search, ratchet, drift, and tree fusing; *init. addseqs* = 100, *find min. length* = 10) with implied weighting (default function, concavity constant *K* = 3.0), which is a technique to down-weight overly homoplastic characters [58]. Preliminary analyses under equal weighting were also performed but generally produced longer trees that were less congruent with morphology-based taxonomy and/or molecular evidence (results not shown). Assessment of clade support using resampling techniques was performed in provisional analyses, but these support values were generally very low, even for well-established taxa (results not shown). We here take the position that quantitative, especially resampling, metrics are of limited value in studies of relatively small morphological matrices with an expected high amount of homoplasy. We therefore took a qualitative approach to evaluating clade support, by looking for potential synapomorphies of sets of genera, using the MP-based tracing function in MacClade 4.08a [59].

It has been argued that model-based approaches for analyzing discrete morphological data are superior to MP [60, 61]. Therefore, we also analyzed the morphological data matrices in the Bayesian framework of MrBayes 3.2.3 [62] using the Markov-k model with a four-rate category gamma correction for among-site rate variation (Mk + G_4_) and *coding = informative* to account for the fact that we only included parsimony-informative characters [63, 64]. We ran 2 × 4 Markov Chain Monte Carlo (MCMC) chains in parallel using Metropolis coupling [65] for 10^6^ generations (sampling every 100), checked for convergence using Tracer 1.6 [66] and discarded the first 10% of samples as burn-in before calculating 50% majority-rule consensus trees (MRCs). For comparison, we also used the maximum likelihood (ML) implementation of the same model as provided by RAxML 8.2.4 [67], using the *-f a* option to perform rapid bootstrapping [68] followed by search for the ML tree. Bootstrapping was automatically stopped using the *autoMRE* option [69].

### Molecular data assembly and phylogenetic analysis

The datasets of Dohrmann et al. [16] – consisting of 18S ribosomal DNA (rDNA), 28S rDNA, mitochondrial (mt) 16S rDNA and mt cytochrome oxidase subunit I (COI) fragments (see [3, 16]) – were supplemented with subsequently published hexactinellid sequences [17, 18, 70]; sequences for 16 additional specimens were newly generated for this study (Table 1).

**Table 1:** Specimen information and sequence accession numbers for newly sampled species

Species	Family	Origin	Voucher	Source	18S	28S	16S	COI
*Schulzeviella* n. sp.	Pheronematidae	Hawaii	P4-224 sp5	HURL	–	LT627545^b^	LT627531	–
*Doconesthes dustinchiversi*	Rossellidae	B.C.	014-00412-001	RBCM	–	–	LT627517	LT627550^a^
*Asconema fristedti*	Rossellidae	Florida	17-XI-05-2-2	HBOI	–	LT627532^b^	LT627516	–
Bolosominae n. gen. n. sp.	Euplectellidae	Hawaii	P4-224 sp7	HURL	–	LT627534^b^	LT627520	LT627552^a^
*Atlantisella* sp.	Euplectellidae	Galapagos	22-X-95-1-7	HBOI	LT627547^b^	LT627533	LT627519	–
*Iphiteon* sp.	Dactylocalycidae	Bahamas	24-V-93-1-7	HBOI	–	LT627537^b^	LT627522	LT627553
*Dactylocalyx* sp.	Dactylocalycidae	Bahamas	22-XI-02-3-13	HBOI	–	LT627538^b^	LT627525	–
*Dactylocalyx pumiceus*	Dactylocalycidae	Bahamas	12-IV-05-1-10	HBOI	LT627548	LT627539	LT627523	–
*Dactylocalyx pumiceus*	Dactylocalycidae	Bonaire	RMNH POR 9215	RMNH	–	LT627540	LT627524	LT627554^a^
*Euryplegma auriculare*	Aulocalycidae	NZ	NIWA 43457	NIWA	LT627546^a^	LT627535^b^	LT627518	LT627551^a^
*Tretopleura* n. sp. 1	Uncinateridae	Hawaii	P5-701 sp4	HURL	–	LT627543^b^	LT627530	LT627555^b^
*Tretopleura* n. sp. 2	Uncinateridae	Hawaii	P4-229 sp10	HURL	–	LT627542^b^	LT627529	LT627556^b^
*Heterorete* sp.	Euretidae	Hawaii	P4-224 sp1	HURL	–	LT627536^b^	LT627521	–
*Homoieurete macquariense*	Euretidae	MR	QM G331848	QM	–	–	LT627528	LT627559^a^
*Cyrtaulon sigsbeei*	Tretodictyidae	Bonaire	RMNH POR 9219	RMNH	LT627549^b^	LT627544^a^	LT627526	LT627557^b^
*Laocoetis perion*	Craticulariidae	Madagascar	DW 3213	MIRIKY	–	LT627541	LT627527	LT627558^b^

Notes: *B.C.* British Columbia, Canada, *MR* Macquarie Ridge, *NZ* New Zealand (specimen supplied by NIWA Invertebrate Collection, NIWA, Wellington), *HURL* Hawaiian Undersea Research Laboratory (samples collected with submersible PISCES), *HBOI* Harbor Branch Oceanographic Institution (samples collected with submersible Johnson-Sea-Link II; most subsamples taken during August 2011 PorToL Integrative Taxonomy Workshop at Ft. Pierce, FL, USA), *QM* Queensland Museum, Brisbane, Australia, *NIWA* National Institute of Water and Atmospheric Research, New Zealand, *RBCM* Royal British Columbia Museum, *RMNH* Naturalis, Leiden, The Netherlands (samples provided by R.W.M. van Soest), *MIRIKY* French Expedition MIRIKY 2009. Underwater photographs of *Heterorete* sp. and *Tretopleura* n. sp. 1 are given in Fig. 1b and c, respectively. ^a^only 5′ half; ^b^only 3′ half

DNA was extracted by boiling small pieces of tissue for 20 min in 20% Chelex (Sigma-Aldrich) (detailed protocol available upon request from MD). Polymerase chain reaction (PCR) was performed with GoTaq (Promega) or MyTaq (Bioline) according to manufacturers’ instructions. Thermal regimes and primers are described in [3, 16]. PCR products were purified either with ExoSAP-IT (Affymetrix) in case of clear single bands, or otherwise cut out from 1% agarose gels and cleaned with a “freeze-squeeze” method adopted from [71] or a QIAquick Gel Extraction kit (QIAGEN). Sanger sequencing was performed with BigDye Terminator chemistry (Applied Biosystems) at the sequencing facility of the University of Alabama, Birmingham, AL, USA and the Sequencing Service of the Department of Biology at LMU Munich. Chromatograms were edited using Codon Code Aligner (Codon Code Corporation) or Geneious 6.1.6 (Biomatters) and consensus sequences manually aligned to the datasets described above.

In addition, 16S rDNA and COI sequences from *Lophophysema eversa*, *Tabachnickia* sp. (Amphidiscophora: Hyalonematidae), and *Vazella pourtalesii* (Lyssacinosida: Rossellidae) were extracted from mt genome sequencing data [72, 73] by aligning the whole-genome sequences to the 16S and COI alignments, respectively, with the profile alignment option in ClustalX 2.1 [74], followed by manual trimming and correction. Full single-gene alignments, including RNA structure information for 18S and 28S rDNA, are available in Additional files 5, 6, 7 and 8.

To check for conflicting phylogenetic signal between markers, single-gene alignments were first analyzed separately in RAxML 8.0.26 [67], after removal of unalignable regions and sites with excessive numbers of gaps. For COI and 16S rDNA, general-time-reversible (GTR) + G_4_ models [64, 75] were employed, and in the 18S and 28S rDNA analyses the S16 + G_4_ paired-sites model (see [76]) was assigned to stem-encoding regions in addition to GTR + G_4_ for loop-encoding regions. We used the *-f a* option to perform rapid bootstrapping [68] with the *autoMRE* option to automatically determine the sufficient number of pseudoreplicates [69]. For the final analyses, all four markers were concatenated in SeaView [77]. For *Dactylocalyx pumiceus*, a hybrid sequence was constructed from specimens HBOI 12-IV-05-1-10 (rDNA) and RMNH POR 9215 (COI) to maximize marker coverage (see Table 1). The concatenated matrix (supermatrix hereafter) consists of 73 taxa and 4806 base pairs (bp), and features 1926 distinct alignment patterns and ~30% missing data (Additional file 9; for RNA structure and partitioning information, see Additional files 10 and 11). Phylogenies were inferred from the supermatrix using ML and Bayesian methods as follows.

For ML analyses, we used RAxML under the models and options described above, assuming a single topology and set of branch lengths across partitions but independent model parameters for each partition. For Bayesian analyses, we used MrBayes 3.2.4 [62] under a model-partitioning scheme analogous to the ML analyses. For 18S and 28S rDNA stem-encoding regions we employed the Doublet model (based on [78]). Structure information was converted from dot-bracket format to MrBayes format using a perl script written by Oliver Voigt (see [79]). We ran 2 × 4 MCMC chains in parallel for 5 × 10^6^ generations, sampling every 100th. Convergence was checked in Tracer 1.6 [66] and 50% of samples were discarded as burn-in before calculating the MRC.

### Combined analysis of molecular and morphological data (“total evidence”)

To investigate whether addition of morphological characters to the molecular dataset would influence tree topologies, we first conducted analyses restricted to those genera with molecular data. For this purpose we split the supermatrix into submatrices representing Hexasterophora and Amphidiscophora, respectively (see above), excluded all but one species each of genera represented by multiple species, and renamed the remaining terminal taxa to match the taxon names in the morphological dataset. Species retained from multi-species genera were chosen as to minimize missing data; those were *C. weddelli* for *Caulophacus*, *R. nuda* for *Rossella*, *N. asconemaoida* for *Nodastrella*, *B. spinosus* for *Bathydorus*, *E.* sp. 1 for *Euplectella*, *I. panicea* for *Iphiteon*, *D. pumiceus* for *Dactylocalyx*, *H. calyx* for *Heterochone*, *A. vastus* for *Aphrocallistes*, *A. australia* for *Aspidoscopulia*, *T.* n. sp. 2 for *Tretopleura*, and *H.* sp. 3 for *Hyalonema. Iphiteon* and *Semperella* were included as outgroups for Amphidiscophora and Hexasterophora, respectively. Morphological characters for the outgroups were all coded as absent (0) in order to mimick the conditions of the morphological analyses (see above). Molecular and morphological partitions were then concatenated in SeaView and analyzed in RAxML and MrBayes as described above (using 10% burnin in the MrBayes analyses, and not accounting for ascertainment bias of the morphological partition in the RAxML analyses due to software limitations). To facilitate comparison, the taxon-reduced molecular supermatrices were also analyzed separately in RAxML.

Next, we attempted to reconstruct complete genus-level phylogenies of the two subclasses. For this purpose we first concatenated the taxon-reduced molecular supermatrices with the complete morphological matrices (resulting in datasets with ~62 and ~65% missing data for Amphidiscophora and Hexasterophora, respectively), and analyzed those datasets in RAxML and MrBayes as described above. Second, we analyzed the data with MP in TNT under the same settings as used for the morphology-only analyses (see above; gaps were treated as missing data). Because TNT does not support mixed data types, we first recoded the sequence data such that A was replaced with 0, C with 1, G with 2, and T with 3; all ambiguous characters (N, R, Y etc.) were recoded as gaps (−). For comparability with the ML analysis, we performed bootstrapping [80] with 550 (Hexasterophora) and 1000 (Amphidiscophora) pseudoreplicates (*init. addseqs* = 10, *find min. length* = 5). Third, we used the weighted version of the “fossil placement” or “morphology-based phylogenetic binning” approach developed by Berger and Stamatakis [22] as implemented in RAxML to place those genera without sequence data onto the molecular backbone (reference) phylogeny (see [22, 81] for details of the method). In these analyses, we also used the Mk + G_4_ model with the Lewis correction to account for ascertainment bias (see above).

### Ancestral state reconstruction

For the purpose of more in-depth investigations of character evolution across Hexactinellida, we first merged the morphological data sets of Amphidiscophora and Hexasterophora, resulting in a matrix of 124 characters for 125 taxa (plus outgroup coded as 0 for all characters; see above) (Additional file 12). We then also included six additional characters that were not used for the purpose of phylogeny reconstruction because they are too prone to homoplasy or overly simplistic representations of complex morphological features (extended matrix in Additional file 13): 1) pinular hexactins (hexactins with a bushy distal ray), 2) basiphytous attachment to the substrate (attachment by a siliceous plate), 3) lophophytous attachment to the substrate (attachment by anchoring spicules), 4) general presence of synapticular fusion (fusion of spicules through siliceous bridges [see Fig. 2d, h]; a merger of characters 23 and 39 in Additional file 2), 3) presence of a lyssacine body plan, and 4) presence of a dictyonal body plan. Finally, we manually combined the total-evidence trees of the two subclasses obtained with TNT (see Results and discussion) and used the resulting tree (included in Additional file 13) for ancestral state reconstruction.

In order to obtain quantitative estimates of the evolution of selected characters and reconstruct ground pattern features of major clades, we used ML ancestral state reconstruction methods as implemented in Mesquite 2.75 [56]. To assess the sensitivity of results to model choice, we employed two different models to calculate proportional likelihoods of ancestral character states: the 1-parameter Mk model (Mk1 [63]) and the asymmetrical Mk 2-parameter model (aMk2 [56]). The Mk1 model assumes equal rates of gains (0 to 1) and losses (1 to 0), whereas under the aMk2 model, the two rates are allowed to differ. For aMk2 analyses, we assumed equilibrium root state frequencies (default in Mesquite 2.75) (we also experimented with equal root state frequencies, although the assumption that the presence and absence of characters in the ancestral glass sponge are equally likely is clearly unrealistic; accordingly, these analyses yielded some contradictory and biologically nonsensical results [not shown]). Because phylogenetic uncertainty can bias ancestral state reconstruction [82] we also evaluated the influence of alternative topological arrangements in crucial parts of the tree on the reconstruction of important characters.

## Results and discussion

### Phylogenies inferred from morphological data

#### Amphidiscophora

**Fig. 4:**
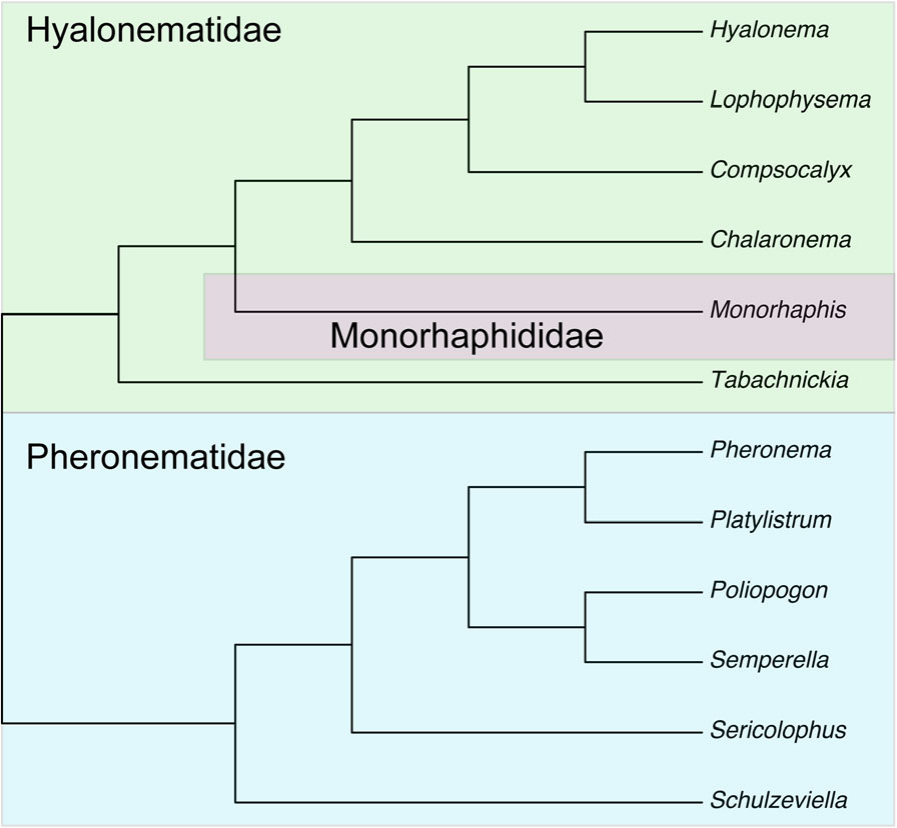
Most parsimonious tree (MPT) of Amphidiscophora inferred with TNT from the morphological data matrix. Treelength (TL) = 60, consistency index (CI) = 0.48, retention index (RI) = 0.65, rescaled CI (RC) = 0.31. Nexus file available at figshare (https://doi.org/10.6084/m9.figshare.3120130.v3)

Maximum-parsimony analysis of the Amphidiscophora matrix found one single most parsimonious tree (MPT) (Fig. 4). Congruent with previous results [3], Pheronematidae is resolved as monophyletic. The position of *Monorhaphis* (the sole representative of Monorhaphididae) is here resolved as being inside Hyalonematidae, rendering this family paraphyletic. However, this result has to be viewed with caution (see supplementary discussion in Additional file 14 available at figshare [https://doi.org/10.6084/m9.figshare.3120130.v3]). In the Bayesian tree (Additional file 15: Figure S1), Pheronematidae is also recovered as monophyletic (posterior probability [PP] = 0.96); four of the five genera of Hyalonematidae form a highly supported clade (PP = 0.98), but the positions of *Tabachnickia* and *Monorhaphis* within Amphidiscophora remain unresolved. The ML phylogeny (Additional file 16: Figure S2) is similar to the MP tree, but with somewhat different branching order within families; with two exceptions, bootstrap support (BS) is very low. For the interested reader, a more detailed account of the relationships within Pheronematidae and Hyalonematidae, and potential character support is provided in the supplementary discussion in Additional file 14.

#### Hexasterophora

**Fig. 5:**
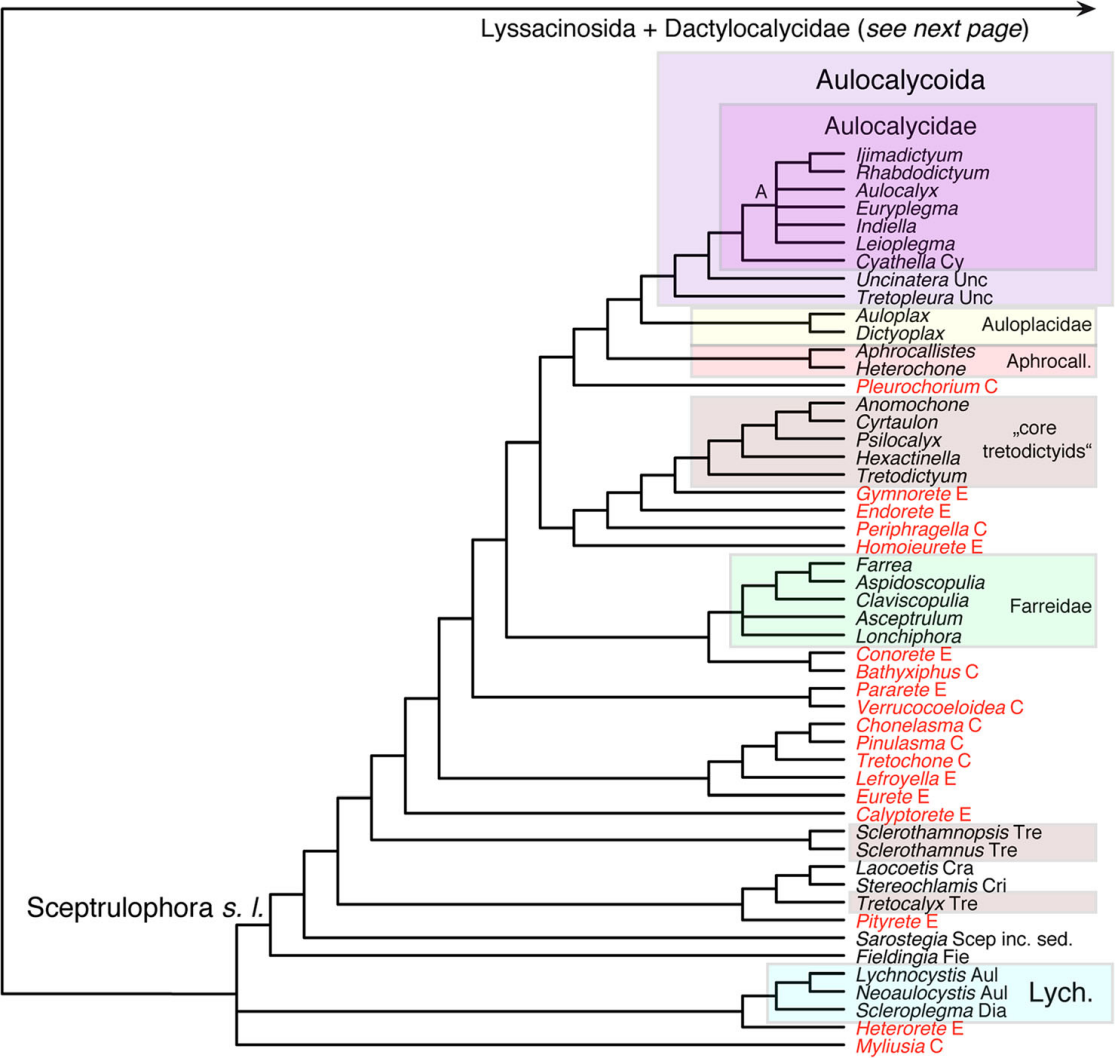
Strict consensus tree of the 46 MPTs of Hexasterophora inferred with TNT from the morphological data matrix. TL = 650, CI = 0.17, RI = 0.64, RC = 0.11. Part 1: A, Aulocalycinae; Aphrocall., Aphrocallistidae; Aul, Aulocystidae; C, Chonelasmatinae; Cra, Craticulariidae; Cri, Cribrospongiidae; Cy, Cyathellinae; D, Diapluridae; E, Euretinae; Fie, Fieldingiidae; Lych., Lychniscosida; Scep inc. sed., Sceptrulophora *incertae sedis*; s. l., *sensu lato*; Tre, Tretodictyidae; Unc, Uncinateridae. Genera currently classified in Euretidae highlighted in light red

**Fig. 6:**
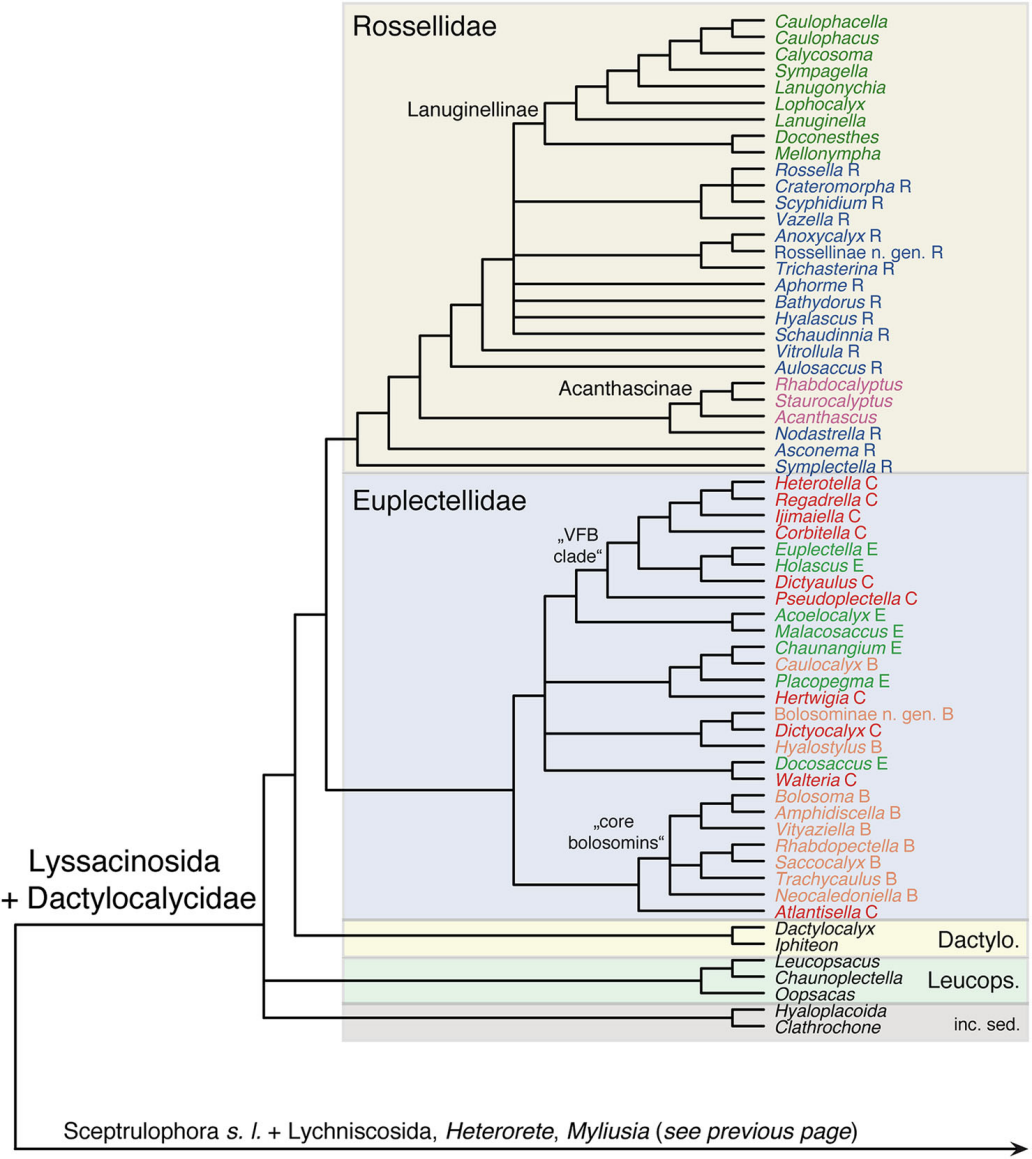
Strict consensus tree of the 46 MPTs of Hexasterophora inferred with TNT from the morphological data matrix. Part 2: TL = 650, CI = 0.17, RI = 0.64, RC = 0.11. B, Bolosominae; C, Corbitellinae; Dactylo., Dactylocalycidae; E, Euplectellinae; inc. sed., *incertae sedis*; Leucops., Leucopsacidae; R, Rossellinae; VFB, venus flower basket. Current subfamily assignment of rossellid and euplectellid genera indicated by different colours. Nexus file available at figshare (https://doi.org/10.6084/m9.figshare.3120130.v3)

Maximum-parsimony analysis of the Hexasterophora matrix resulted in 46 MPTs, the strict consensus of which is shown in Figs. 5 and 6. Of the four orders of Hexasterophora, only Aulocalycoida and the small relict group Lychniscosida are recovered as monophyletic. The genus *Heterorete* (Fig. 1b; currently in Sceptrulophora: Euretidae, although lacking sceptrules and uncinates) is reconstructed as the sister group of Lychniscosida, and Aulocalycoida is deeply nested within Sceptrulophora as the sister group of Auloplacidae. Lychniscosida + *Heterorete* and the Sceptrulophora *sensu lato* (*s. l.*) clade together form a clade with the second sceptrule- and uncinate-lacking euretid genus, *Myliusia*, the exact placement of which is not resolved. In agreement with molecular results [3, 15, 16], Dactylocalycidae comes out closer to Lyssacinosida than to Sceptrulophora. However, it is here reconstructed as the sister group to a Euplectellidae + Rossellidae clade to the exclusion of Leucopsacidae and the two Lyssacinosida *incertae sedis* (*inc. sed.*) genera, rendering Lyssacinosida paraphyletic.

Of the 12 hexasterophoran families with more than one genus, nine are recovered as monophyletic groups (Aphrocallistidae, Aulocalycidae, Aulocystidae, Auloplacidae, Dactylocalycidae, Euplectellidae, Farreidae, Leucopsacidae, Rossellidae) and three are inferred to be para- or polyphyletic (Uncinateridae, Euretidae, Tretodictyidae; for the latter family, five of the eight genera form a clade [“core tretodictyids”]). Within Rossellidae, only subfamilies Lanuginellinae (*sensu* [83]) and Acanthascinae (cf. [51]) are monophyletic; Rossellinae is not recovered as a natural group, congruent with previous results [3]. Boury-Esnault et al. [83] moved *Caulophacus* and *Caulophacella* from Rossellinae to Lanuginellinae, mostly based on molecular evidence [16, 83], which we confirm here with our cladistic analysis of morphological data (since the revised diagnosis of Lanuginellinae provided by [83] is rather vague, we provide a more concise and comprehensive summary based on our character analysis in Appendix 2). Within Euplectellidae, a clade of genera with the iconical “venus-flower basket” body shape (Fig. 1f) (“VFB clade”) and a clade comprising most genera of the stalked subfamily Bolosominae (Fig. 1g) (“core bolosomins”) is recovered; subfamilies Euplectellinae and Corbitellinae are clearly not recovered as natural groups (see also [16]). Overall, the Bayesian tree (Additional file 17: Figure S3) is similar but less well resolved than the MP tree. In contrast, the ML tree (Additional file 18: Figure S4) displays a vastly different topology (and branch lengths), and appears in large parts highly incongruent with current taxonomy and/or molecular evidence (e.g., paraphyletic Rossellidae basal to the remaining taxa). For a more detailed account, the interested reader is referred to the supplementary discussion in Additional file 14.

### Phylogenies inferred from molecular data

#### Congruence between markers

Topologies of the four single-gene trees were largely congruent, only differing in poorly supported regions (Additional files 19, 20, 21 and 22: Figures S5–S8). A notable exception to this was monophyly of *Aphrocallistes* in the 28S phylogeny (Additional file 21: Figure S7), as discussed previously [19]. Another conflict involved the position of *Dactylocalyx* sp., which was placed inside *D. pumiceus* (BS = 85%) in the 16S tree (Additional file 19: Figure S5), whereas *D. pumiceus* was monophyletic (BS = 70%) in the 28S tree (Additional file 21: Figure S7). Currently, there are only two accepted species of *Dactylocalyx*, although more nominal species exist [5, 84]. Our *Dactylocalyx* sp. might represent a so far undescribed species (HMR, pers. obs.), but our results further demonstrate that the genus is in urgent need of revision, preferably using a combined morphological/molecular approach.

#### Phylogenetic analyses of concatenated markers

**Fig. 7:**
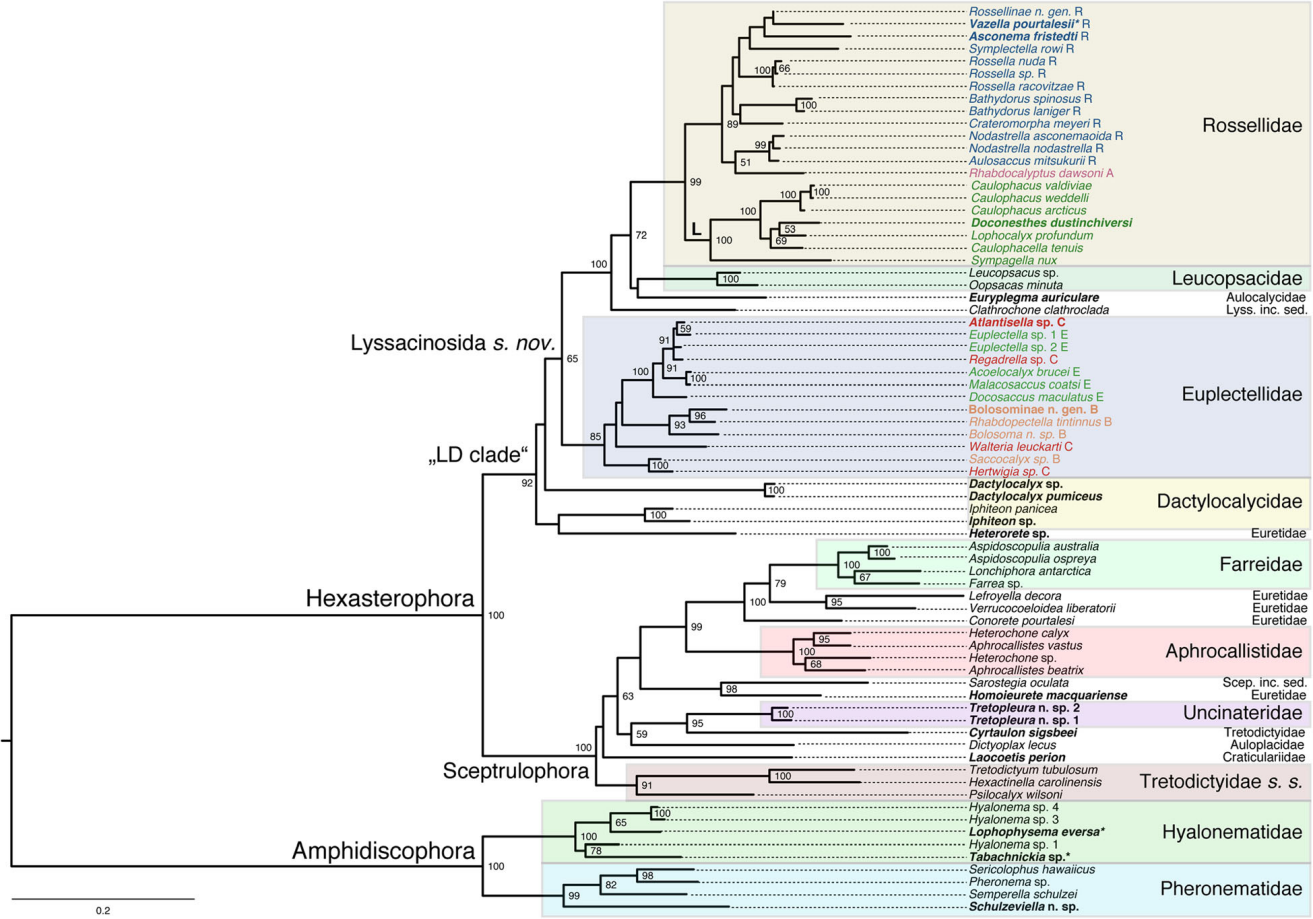
Phylogeny of Hexactinellida inferred from concatenated molecular markers with RAxML. Bootstrap values >50% shown on branches (based on 600 pseudoreplicates). Newly sampled species highlighted in ***bold***. *, 16S and COI sequence data from mitochondrial genome sequencing projects [72, 73]. A, Acanthascinae; B, Bolosominae; C, Corbitellinae; L, Lanuginellinae; Lyss inc. sed., Lyssacinosida *incertae sedis*; R, Rossellinae; Scep inc. sed., Sceptrulophora *incertae sedis*; s. nov., *sensu novo*; s. s., *sensu stricto*. Current subfamily assignment of rossellid and euplectellid genera indicated by different colours. Scale bar, expected number of substitutions per site. Nexus file available at figshare (https://doi.org/10.6084/m9.figshare.3120130.v3)

Figure 7 shows the ML phylogram obtained from the supermatrix. The Bayesian tree is largely congruent with this phylogeny and is given in Additional file 23: Figure S9. Corroborating previous analyses (reviewed in [14]), Hexactinellida is divided into three major, well-supported clades: Amphidiscophora, Sceptrulophora, and a clade containing Lyssacinosida and Dactylocalycidae (“LD clade” hereafter). Below we discuss relationships within these clades, but for the sake of brevity, we will refrain from describing results that are unchanged compared to previous studies [3, 15–18, 70].

***Amphidiscophora*** *.* Addition of *Schulzeviella*, *Tabachnickia*, and *Lophophysema* confirms monophyly of families Pheronematidae and Hyalonematidae. Among Pheronematidae, *Schulzeviella* n. sp. is sister to the remaining sampled genera, consistent with the morphology-based tree (Fig. 4). Among Hyalonematidae, we find the mega-diverse genus *Hyalonema* (120 spp. in 12 subgenera) to be polyphyletic: *Hyalonema* sp. 3 and *Hyalonema* sp. 4 are sister to *Lophophysema eversa*, whereas *Hyalonema* sp. 1 is sister to *Tabachnickia* sp. Because *H*. sp. 3 and 4 are likely members of *H.* (*Cyliconema*) whereas *H*. sp. 1 likely belongs to *H.* (*Corynonema*) (MD, pers. obs.), this result is in line with the view that at least some of the subgenera of *Hyalonema* should actually be classified as separate genera [85]. However, statistical support for the positions of *Lophophysema* and *Tabachnickia* is only moderate to low. Clearly, increased taxon sampling of *Hyalonema* spp., preferably including representatives of all 12 subgenera, will be necessary to support a revised classification of this large complex of morphologically poorly differentiated species.

***Sceptrulophora***. In this study, we have added five additional sceptrule-bearing species to the molecular dataset: *Tretopleura* n. sp. 1 and 2 (Uncinateridae; Fig. 1c), *Laocoetis perion* (the sole extant survivor of the paleontologically important Craticulariidae), the recently described euretid *Homoieurete macquariense* [44], and the tretodictyid *Cyrtaulon sigsbeei*. In the ML tree, *Laocoetis* is the sister taxon to a clade containing all sampled sceptrulophorans except *Tretodictyum*, *Hexactinella*, and *Psilocalyx* (Tretodictyidae *sensu stricto* [*s. str.*] hereafter). However, BS for this arrangement is very low, and in the Bayesian tree the position of this longest-ranging hexactinellid genus (since the Late Jurassic [86]) with respect to Tretodictyidae *s. str.* and the remaining sceptrulophorans remains unresolved. A clade including all sceptrulophorans except *Laocoetis* and Tretodictyidae *s. str.* is found in both the ML and Bayesian trees, but only receives significant support in the latter (PP = 0.99). Within this clade, *Cyrtaulon* is reconstructed as the sister group (within the current taxon sampling) of *Tretopleura* with high support, thus rejecting inclusion of *Cyrtaulon* in Tretodictyidae. *Homoieurete* forms a highly supported clade with *Sarostegia* (Sceptrulophora *inc. sed.*) that is sister to the well-established Aphrocallistidae + Euretidae *part*. + Farreidae clade (cf. [17]) in the ML tree, and sister to a *Dictyoplax* (Auloplacidae) + *Cyrtaulon*/*Tretopleura* clade in the Bayesian tree (both with low statistical support).

The position of *Tretopleura* within Sceptrulophora, combined with the fact that the two new species definitely bear sceptrules and uncinates (MD and HMR, pers. obs.), necessitates that Uncinateridae be moved from Aulocalycoida to Sceptrulophora within the Linnean classification (Appendix 2). The position of *Cyrtaulon* outside Tretodictyidae is not too unexpected given that this taxon lacks some typical morphological features of the family (see Additional file 14). On the other hand, potential synapomorphies with *Tretopleura* remain elusive to us. However, the long branches separating these two genera indicate that they are probably part of a larger clade including many of the so far unsampled genera of Sceptrulophora, making conclusions about morphological similarities (or lack thereof) premature. The same might be true for the highly supported position of *Homoieurete* as sister to *Sarostegia*, which is equally surprising from a morphological point of view. In any case, these results demonstrate that the scope and definition of Tretodictyidae and especially Euretidae are far from being stable – clearly, more genera of both of these families need to be sampled for molecular phylogenetics. As an interim solution, we remove *Homoieurete* and *Cyrtaulon* from their respective families and treat them as Sceptrulophora *inc. sed.* within the Linnean classification (Appendix 2), following Reiswig and Dohrmann [17].

***LD clade***. Dactylocalycidae (currently in Hexactinosida) was so far only represented by *Iphiteon panicea* in molecular phylogenies (reviewed in [14]). We here included another, possibly new, species of that genus, as well as two species (one possibly new) of the second and type genus of the family, *Dactylocalyx*. Whereas monophyly of *Iphiteon* and *Dactylocalyx*, respectively, is confirmed here, we did not recover monophyly of the family: *Iphiteon* is weakly reconstructed as sister to *Heterorete* (Euretidae, discussed below) and the position of *Dactylocalyx* with respect to *Iphiteon*/*Heterorete* and the remainder of the LD clade is basically unresolved (polytomy in the Bayesian tree and BS < 50% for a position closer to Lyssacinosida in the ML tree). However, given this poor resolution, our results do not provide strong evidence for non-monophyly of Dactylocalycidae (see also Additional file 14 and next section), so this family should be retained in the Linnean system for the time being. One reason for this lack of resolution could be that so far unsampled or undiscovered taxa might be related to *Iphiteon*, *Dactylocalyx*, and *Heterorete*, and would have to be included to resolve this part of the phylogeny. In any case, however, our molecular (and morphological; see above) analyses confirm that both *Iphiteon* and *Dactylocalyx* are more closely related to Lyssacinosida than to the remaining Hexactinosida (Sceptrulophora). We therefore here abolish the order Hexactinosida from the Linnean classification and elevate Sceptrulophora from subordinal [19] to ordinal status (Appendix 2). Pending further evidence, and given that recognizing the LD clade as a Linnean taxon is problematic (see below), we here treat Dactylocalycidae as Hexasterophora *inc. sed.* (Appendix 2).

The enigmatic dictyonal genus *Heterorete* (Fig. 1b; currently in Euretidae) is here included for the first time in a molecular phylogenetic study. As this taxon lacks sceptrules and uncinates it is not too surprising that it comes out closer to Lyssacinosida than to Sceptrulophora. One reason (but see also above) for our inability to confidently infer the exact position of this genus is likely the low gene coverage (Table 1); additional, better preserved specimens are needed to obtain more sequence data from this important taxon. Regardless of its exact position, however, our results clearly reject an affinity of *Heterorete* to Sceptrulophora, and it is best treated as Hexasterophora *inc. sed.* for the time being (Appendix 2).

The three lyssacinosidan families (Euplectellidae, Rossellidae, Leucopsacidae) and *Clathrochone* (Lyssacinosida *inc. sed.*) group together in a clade (BS = 65%, PP = 0.97). However, this clade also includes *Euryplegma auriculare*, which is the first-ever sampled representative of Aulocalycidae (Aulocalycoida), a family of dictyonal taxa without confirmed sceptrules or uncinates. *Euryplegma* nests within a maximally supported clade that is sister to Euplectellidae; within this clade *Clathrochone* is the earliest-branching genus. In the Bayesian tree, *Euryplegma* is weakly resolved as sister to Leucopsacidae + Rossellidae (PP = 0.51), whereas in the ML tree it weakly groups with Leucopsacidae (BS < 50%). A relationship of *Euryplegma* (and other aulocalycids) to Lyssacinosida, and especially Leucopsacidae, is consistent with some early taxonomic ideas (see historical overview in [87]). The firm placement of *Euryplegma* among lyssacinosidans implies convergent evolution of a dictyonal framework in this taxon (further discussed below) and renders Lyssacinosida (*sensu* [88]) paraphyletic. However, instead of abolishing this order, we here emend its diagnosis and broaden its scope to include Aulocalycidae; consequently, the order Aulocalycoida is abolished from the Linnean classification (Appendix 2).

Monophyly of Euplectellidae is supported in both the ML and Bayesian trees (BS = 85%, PP = 0.99). In line with previous molecular results [16] and the morphological analysis (Figs. 5 and 6), our phylogenies are inconsistent with the current subfamilial division of Euplectellidae (see below and Additional file 14). The topology is somewhat different to that obtained in [16], but this concerns only weakly supported nodes. The new genus of Bolosominae from Hawaii firmly groups with *Rhabdopectella* in both trees. The newly sampled *Atlantisella* sp. appears closely related to *Euplectella* and *Regadrella*. In fact, it is sister to *Euplectella* sp. 1 to the exclusion of *Euplectella* sp. 2, rendering *Euplectella* paraphyletic. However, this exact position receives insignificant support in the ML tree (BS = 59%). Moreover, branch lengths in this part of the tree are very short, suggesting that these three genera might be the product of a recent radiation that is likely difficult to resolve with the current set of markers.

Regarding the newly included taxa within Rossellidae, *Vazella pourtalesii* groups with *Symplectella rowi* (see [70]) in the Bayesian tree and with Rossellinae n. gen. (Reiswig & Kelly, in prep.) in the ML tree. However, neither of these positions is significantly supported. *Asconema fristedti* appears to be related to the three aforementioned genera, but its exact position is likewise poorly supported. *Doconesthes dustinchiversi* [55] is firmly nested in Lanuginellinae, further confirming monophyly of this subfamily (*sensu* [83]), although its exact position as sister to *Lophocalyx* remains uncertain due to low support values.

#### Maximum-likelihood analyses of the reduced supermatrices

The tree inferred from the Amphidiscophora supermatrix reduced to only one species per genus was fully consistent with the tree inferred from the complete matrix (not shown). Likewise, in the tree reconstructed from the reduced Hexasterophora matrix (Additional file 24: Figure S10), only a few minor differences concerning nodes with low BS are observed. Thus, reducing the taxon sampling of the molecular supermatrix to match the taxonomic level of the morphological matrix had no adverse effects on the inferred relationships.

### Phylogenies inferred from combined molecular and morphological data

#### Combined analyses restricted to genera with sequence data

Not surprisingly – given the small number of informative characters available for this subclass – addition of morphological data to the Amphidiscophora matrix had no effect on the tree topology; only some BS values slightly decreased (not shown). In contrast, addition of morphological characters to the Hexasterophora matrix had some noticeable effects: In the ML tree (Additional file 25: Figure S11), the exact positions of *Asconema*, *Rossella*, and *Atlantisella* within Rossellidae and Euplectellidae, respectively, changed (albeit with poor BS), Dactylocalycidae came out monophyletic (also with weak support), and support for monophyly of Lyssacinosida (*sensu novo* [*s. nov.*]), Euplectellidae, Tretodictyidae *s. str.*, and *Lophocalyx* + *Doconesthes* substantially increased. In the MrBayes tree (Additional file 26: Figure S12), the position of *Homoieurete* + *Sarostegia* changed (PP = 0.88), Dactylocalycidae came out monophyletic with high support (PP = 0.97), *Heterorete* was reconstructed as sister to the remaining LD clade (PP = 0.97, but note that support for the LD clade as a whole decreased to 0.79), and the topology within Rossellidae changed (similar to the ML analysis, albeit with overall less resolution). These results suggest that the morphological characters indeed harbor additional signal in support of some clades and can have an impact on phylogenetic inference, despite being much smaller in number than the molecular characters.

#### Combined analyses including all genera

**Fig. 8:**
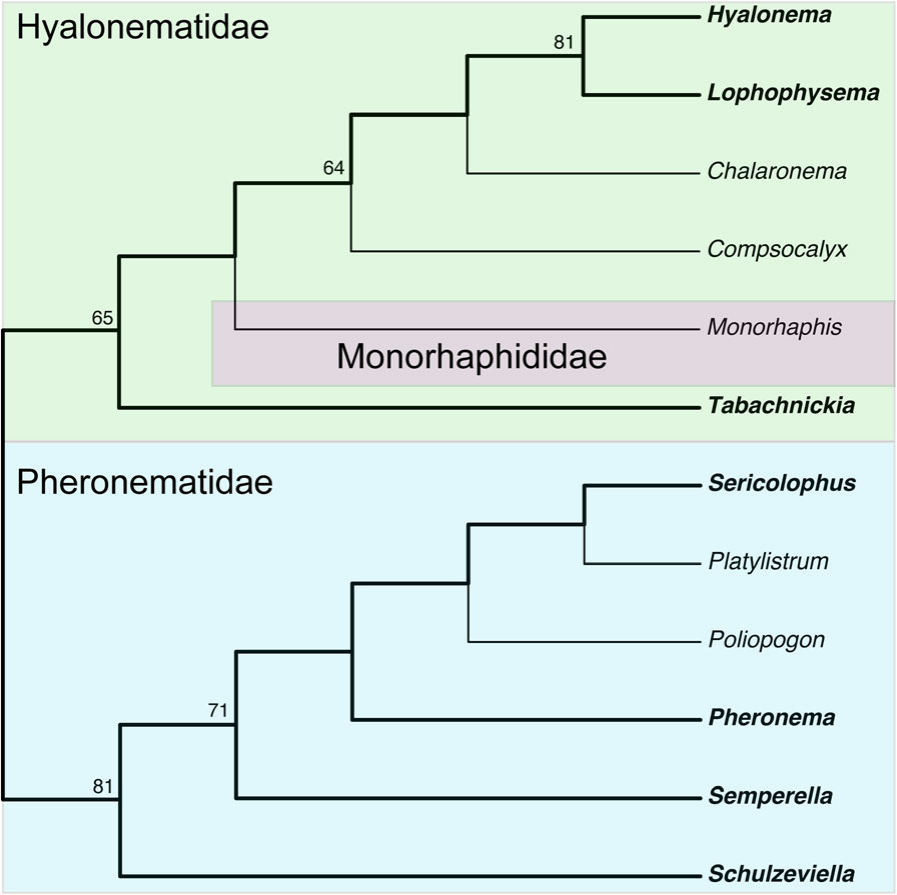
Phylogeny of Amphidiscophora inferred with TNT from concatenated molecular and morphological data, including all genera. Genera with sequence data highlighted in bold and connected with thick branches. Bootstrap values >50% shown on branches (based on 1000 pseudoreplicates). TL = 2704, CI = 0.78, RI = 0.54, RC = 0.42. Nexus file available at figshare (https://doi.org/10.6084/m9.figshare.3120130.v3)

**Fig. 9:**
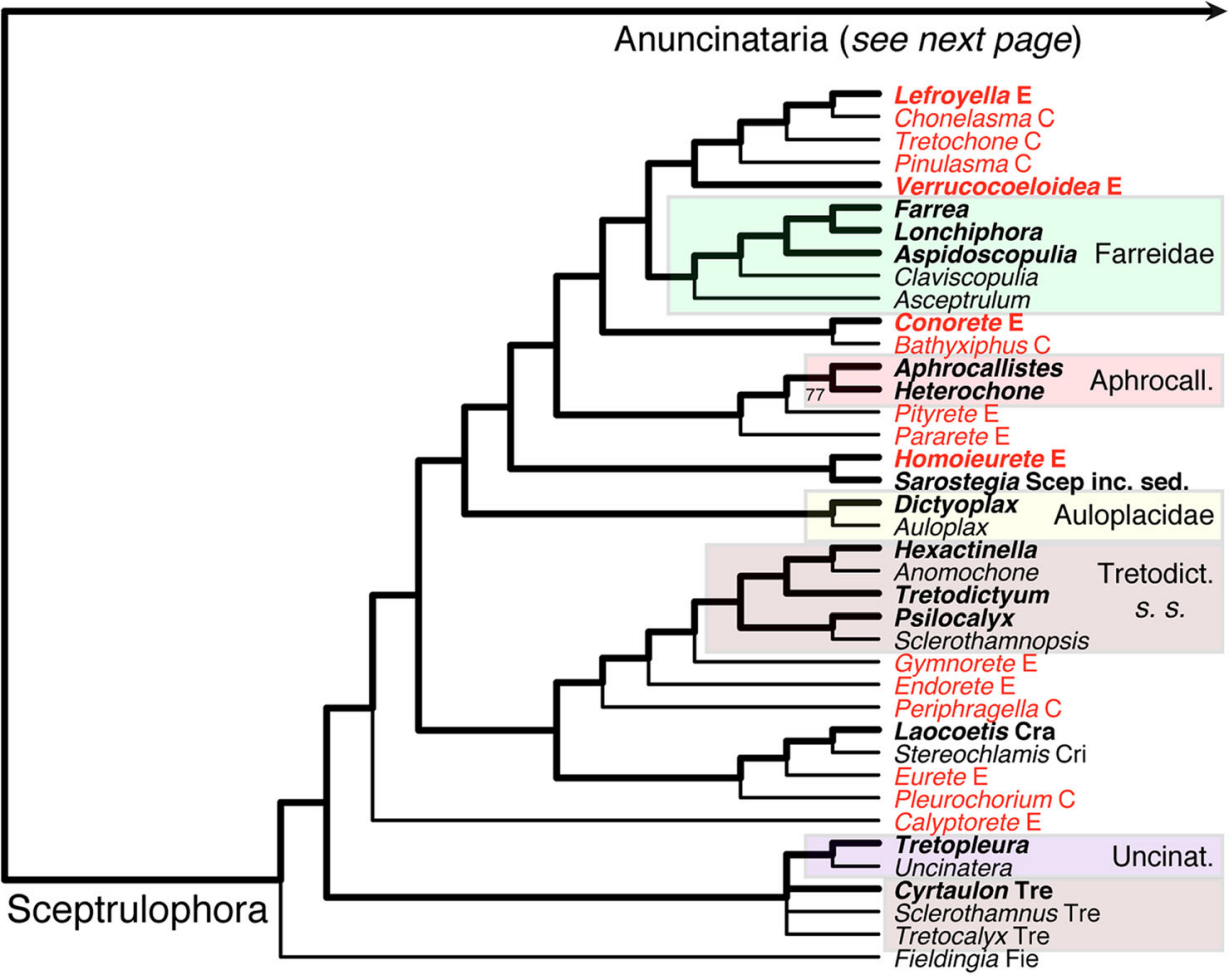
Phylogeny of Hexasterophora inferred with TNT from concatenated molecular and morphological data, including all genera. Genera with sequence data highlighted in bold and connected with thick branches. Bootstrap values >50% shown on branches (based on 1000 pseudoreplicates). TL = 13,147, CI = 0.28, RI = 0.53, RC = 0.15. Part 1: Aphrocall., Aphrocallistidae; C, Chonelasmatinae; Cra, Craticulariidae; Cri, Cribrospongiidae; E, Euretinae; Fie, Fieldingiidae; Scep inc. sed., Sceptrulophora *incertae sedis*; s. s., *sensu stricto*; Tre, Tretodictyidae; Unc, Uncinateridae. Genera currently classified in Euretidae highlighted in light red

**Fig. 10:**
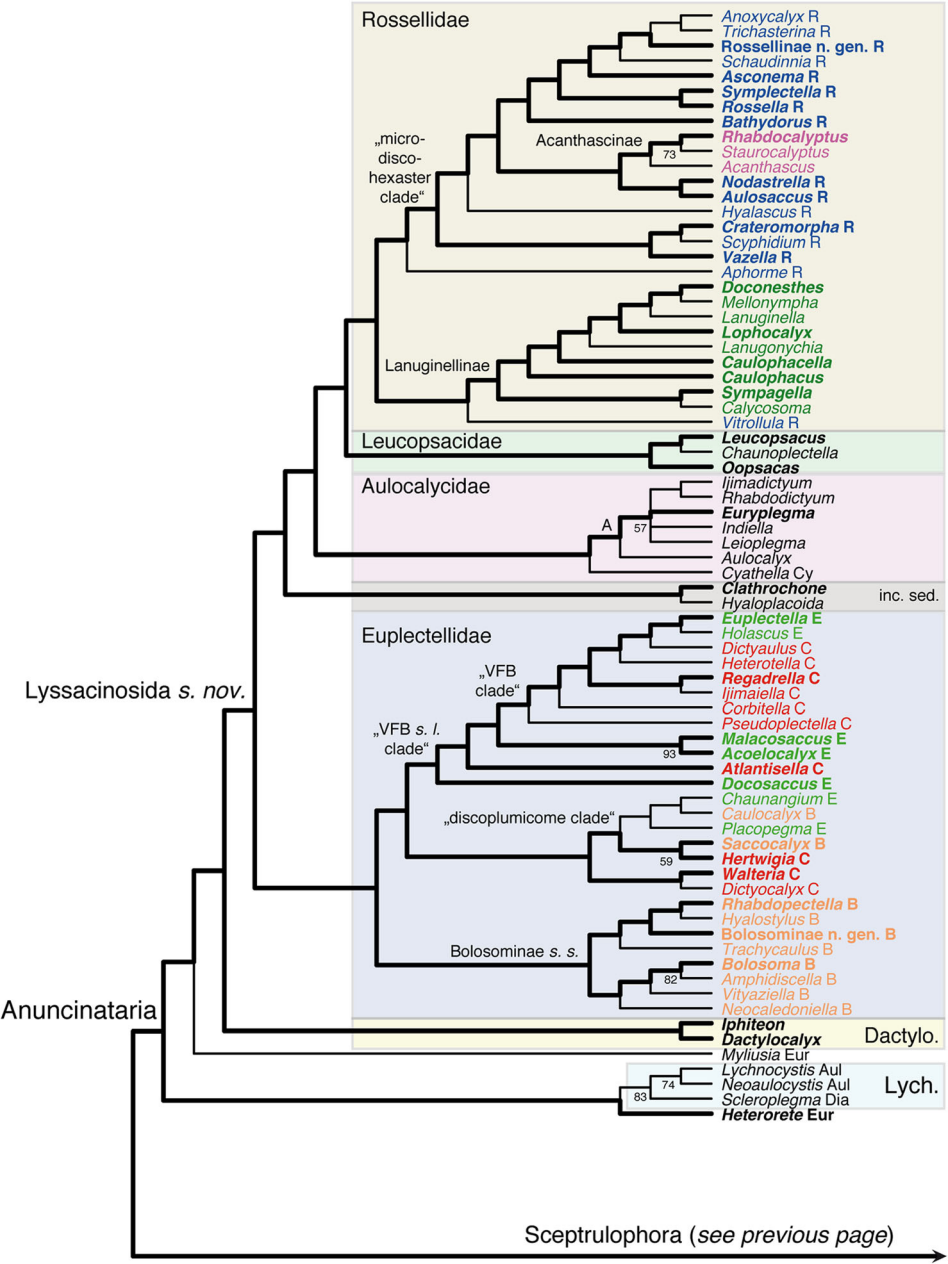
Phylogeny of Hexasterophora inferred with TNT from concatenated molecular and morphological data, including all genera. Part 2: Genera with sequence data highlighted in bold and connected with thick branches. Bootstrap values >50% shown on branches (based on 1000 pseudoreplicates). TL = 13,147, CI = 0.28, RI = 0.53, RC = 0.15. A, Aulocalycinae; Aul, Aulocystidae; B, Bolosominae; C, Corbitellinae; Cy, Cyathellinae; Dia, Diapluridae; Dactylo., Dactylocalycidae; E, Euplectellinae; Eur, Euretidae; inc. sed., *incertae sedis*; Lych., Lychniscosida; R, Rossellinae; s. l., *sensu lato*; s. nov., *sensu novo*; s. s., *sensu stricto*; VFB, venus flower basket. Current subfamily assignment of rossellid and euplectellid genera indicated by different colours. Nexus file available at figshare (https://doi.org/10.6084/m9.figshare.3120130.v3)

The Bayesian, ML, and MP analyses we used to obtain complete genus-level phylogenies of the two subclasses of Hexactinellida all produced poorly supported trees, i.e., with low (<0.95, < 70%) to very low (<0.5, < 50%) PP and BS values for most branches (for the morphological binning analyses, quantitative support was not assessed). We suspect that these low values are caused by the high amount of missing data (in the molecular partition), which is known to pose challenges for phylogenetic tree space exploration [89, 90]. Since quantitative support measures were not very useful in this situation, we again took a qualitative approach and looked for characters that might provide potential synapomorphies of groups of genera, as well as overall congruence of trees with well-supported taxonomic/phylogenetic hypotheses. Of the four approaches, the MP analyses produced the most plausible results (Figs. 8 and 9). In contrast, except for the Amphidiscophora Bayesian analysis (Additional file 27: Figure S14; see below), the ML and Bayesian analyses and the morphological binning approach yielded trees (Additional files 28, 29, 30, 31 and 32: Figures S13, S15-S18) that are less congruent with taxonomy and/or molecular evidence. For instance, monophyly of Euplectellidae was not recovered from these analyses (Additional files 30, 31 and 32: Figures S16-S18). The better performance of MP was somewhat surprising since this method is unable to account for multiple substitutions in molecular data sets, potentially leading to long-branch attraction artifacts [91]. Furthermore, biochemical background knowledge, such as higher frequency of transitions over transversions or coevolution of paired sites in ribosomal RNA cannot be incorporated, which could further bias results. Indeed, when we analyzed the molecular partition alone in TNT, we obtained a topology that was somewhat different from the ML tree, showing some “irregularities” such as *Heterorete* + *Iphiteon* sister to Euplectellidae (results not shown). However, the conflicting nodes had very low BS and the rest of the topology was largely congruent with the ML and Bayesian trees, indicating that phylogenetic signal in the molecular partition is fairly clear and robust to method choice. Apparently, MP was then better at handling the morphological data added in the total-evidence matrix, and this additional information helped to improve the overall result. In contrast, ML and Bayesian methods might not be able to correctly “model” morphological evolution, which might be causing their poorer performance. In-depth investigations of these issues are beyond the scope of the present paper, but our results (see also above for performance of ML on the morphological matrix) indicate that currently available model-based approaches to phylogenetic analysis of morphological data might not always be the best choice (*contra* [60, 61]). Below, we will only discuss the MP trees and regard these – with some caveats – as the currently best-supported working hypotheses for the phylogenetic relationships between all glass sponge genera. For Amphidiscophora, one single MPT was found (Fig. 8), whereas for Hexasterophora nine MPTs were found, the strict consensus of which is presented here (Figs. 9 and 10).

***Amphidiscophora***. The total-evidence tree of Amphidiscophora (Fig. 8) is fully congruent with the molecular phylogenies (Fig. 7, Additional file 23: Figure S9). As in the morphology-based tree (Fig. 4), *Monorhaphis* (Monorhaphididae) is nested within Hyalonematidae, rendering the family paraphyletic. However, this result has to be viewed with caution (see Additional file 14). Indeed, the Bayesian analysis weakly resolved Hyalonematidae as monophyletic, with *Monorhaphis* being sister to all remaining amphidiscophorans (Additional file 27: Figure S14). A possible synapomorphy of Hyalonematidae and Pheronematidae to the exclusion of *Monorhaphis* is the presence of anchorate basalia (attachment spicules with anchor-like distal ends). However, molecular data of *Monorhaphis* are required to further test its phylogenetic position within Amphidiscophora. The remaining part of the Hyalonematidae topology is congruent with the morphology-based tree (Fig. 4) in showing a sister-group relationship of *Hyalonema* and *Lophophysema*. However, the positions of *Compsocalyx* and *Chalaronema* are reversed, character support for which remains unclear. In any case, for a comprehensive understanding of character evolution within Hyalonematidae, all subgenera of *Hyalonema* need to be included in future studies, given the molecular evidence for non-monophyly of this genus (see above).

As in the morphology-based tree (Fig. 4), *Schulzeviella* forms the sister group to the remaining pheronematids. In contrast, addition of the molecular data substantially changed the rest of the topology: *Semperella* branches off after *Schulzeviella*, and *Pheronema* and *Poliopogon* are successive sister groups to a *Sericolophus* + *Platylistrum* clade. Regarding its implications for character evolution, this arrangement appears somewhat more plausible than the morphology-only topology. Choanosomal stauractins (four-rayed spicules in the skeleton of the middle tissue layer) are reconstructed as a synapomorphy of all genera except *Schulzeviella* (secondarily lost in *Sericolophus*); choanosomal tauactins (three-rayed spicules) are reconstructed as a synapomorphy (convergent to *Monorhaphis*, where they are the dominant spicules, however) for *Poliopogon*, *Sericolophus*, and *Platylistrum*; and *Sericolophus* and *Platylistrum* share the secondary absence of macramphidiscs (amphidiscs of the largest size class). Only the placement of *Pheronema* remains elusive in terms of character support. Interestingly, *Poliopogon*, *Sericolophus*, and *Platylistrum* share an asymmetric body shape, with the atrial (exhalant) surface exposed and directed to one side. This character was not included in the matrix, but could be another synapomorphy uniting these three genera (although see [92], who suggest that the body shape of *Sericolophus* resulted from an evolutionary pathway independent of the one relating *Poliopogon* and *Platylistrum*).

***Deep phylogeny of Hexasterophora***. The total-evidence tree of Hexasterophora (Figs. 9 and 10) is also largely congruent with the molecular phylogenies (Fig. 7, Additional file 23: Figure S9) and the trees inferred from the total-evidence matrix restricted to sequenced genera (Additional files 25 and 26: Figures S11-S12). In contrast to the morphology-only tree (Fig. 5), the Lychniscosida + *Heterorete* clade is now resolved as the sister group of the LD clade plus *Myliusia* (currently in Euretidae, but lacking sceptrules and uncinates), and Dactylocalycidae and Lyssacinosida *s. nov.* are reciprocally monophyletic. The latter not only includes *Euryplegma*, but the entire family Aulocalycidae as the sister group of Leucopsacidae + Rossellidae, which is in strong contrast to the morphology-only tree, where this taxon is deeply nested within Sceptrulophora (Fig. 5). Thus, according to these results, dictyonal frameworks of the “aulocalycoid” construction type (Fig. 2d; see [2] and Additional file 4 for details of framework construction) evolved entirely independently from dictyonal skeletons found in other taxa. Furthermore, the major division in Hexasterophora appears to be between Sceptrulophora and a clade containing all taxa that lack sceptrules and uncinates (and not between dictyonal and lyssacine taxa). For the latter we here propose the name “Anuncinataria”. This name is to be preferred over “Asceptrulophora” because uncinates also occur in most species of Amphidiscophora and hence the lack of uncinates might be a derived feature of this group. Even if uncinates evolved convergently in Amphidiscophora and Sceptrulophora (see section Maximum-likelihood ancestral state reconstruction and [93]), the absence of uncinates has at least some diagnostic value. However, we refrain from erecting a Linnean taxon for Anuncinataria for three reasons: 1) no meaningful *positive* morphological diagnosis can be provided for this clade; 2) super- or suborders would have to be introduced, but the number of ranks should be kept at a minimum; and 3) it is very important that the monophyly of this proposed group is first further tested with molecular data from Lychniscosida and *Myliusia*. However, we consider the morphological evidence for the placement of *Myliusia* outside Sceptrulophora sufficient to remove it from Euretidae and therefore re-classify it as Hexasterophora *inc. sed.* within the Linnean system (Appendix 2). On a historical note, it should be pointed out that our Anuncinataria concept was principally long foreshadowed by Schulze [94]. In his “genealogical tree” (his Figs. 9 and 10, p. 495) this author already divided Hexasterophora (although not in his Linnean classification) into a group with uncinates (Uncinataria, which later became Sceptrulophora [93]) and an unnamed group containing lyssacinosidans and his “Maeandrospongidae”, which included *Aulocystis* (= *Neoaulocystis*) and *Scleroplegma* (Lychniscosida), *Dactylocalyx*, *Margaritella* (= *Iphiteon*), and *Myliusia* [20].

Parallel fusion of the rays of hexactine choanosomal megascleres is present in the dictyonal frameworks of most sceptrulophorans (except Auloplacidae and Uncinateridae), Lychniscosida, *Heterorete*, *Myliusia*, as well as *Cyathella* (Aulocalycidae). According to the parsimony mapping in MacClade, this character evolved in the last common ancestor (LCA) of Hexasterophora and got subsequently lost in the LCA of Dactylocalycidae + Lyssacinosida (followed by a “reversal” in *Cyathella*). Thus, these results suggest that dictyonal skeletons with parallel ray fusion are an autapomorphy of Hexasterophora, and the lyssacine body plan evolved secondarily within Anuncinataria from a dictyonal ground pattern (see further discussion in section Maximum-likelihood ancestral state reconstruction; see also [15]).

Besides the loss of uncinates (but see section Maximum-likelihood ancestral state reconstruction), another potential autapomorphy of Anuncinataria is the ability to produce microscleres with floricoidal tips (paw-shaped distal ends of the secondary rays of hexasters and their derivatives). Such spicules are present in *Myliusia*, *Heterorete* (HMR & MD, unpubl. obs.), *Leucopsacus* (Leucopsacidae), some Acanthascinae (Rossellidae), and widespread in Euplectellidae (where they are called floricomes; Fig. 3b), but are unknown from sceptrulophorans. However, the scattered nature of this character across Anuncinataria greatly limits its diagnostic/phylogenetic value. Similarly, atrial megascleres (structural spicules of the inner surface layer) dominated by hexactins could be synapomorphic for *Myliusia*, Dactylocalycidae, and Lyssacinosida, but multiple absences in the latter (e.g., in Aulocalycidae) and presence in some sceptrulophorans weaken the usefulness of this character. Morphological character support for the Dactylocalycidae + Lyssacinosida clade is largely restricted to diactin (two-rayed) megascleres, which are very rare in Sceptrulophora (present in only four genera) and were lost twice, in *Acoelocalyx* + *Malacosaccus* (Euplectellidae) and in Aulocalycinae excl. *Aulocalyx* (see also Additional file 14). Finally, support for the sister-group relationship of *Heterorete* and Lychniscosida is largely limited to fused surface networks, a character that also occurs in some sceptrulophorans and is likely rather prone to homoplasy (see also Additional file 14). Clearly, inclusion of sequence data from Lychniscosida and *Myliusia*, as well as increased gene sampling of *Heterorete* (see section Phylogenies inferred from molecular data) are necessary to better resolve the phylogenetic placement of these key taxa and ultimately test the monophyly of Anuncinataria.

Below, we summarize the main findings of our total-evidence analysis concerning the internal relationships of Sceptrulophora and Lyssacinosida. For a more detailed account, the interested reader is referred to the supplementary discussion in Additional file 14.

***Sceptrulophora***. Relationships within Sceptrulophora are substantially altered compared to those inferred from the morphological data only (Fig. 5), especially concerning the deeper branching order, which appears to be largely driven by the molecular characters. Uncinateridae (*Uncinatera* + *Tretopleura*) is reconstructed as monophyletic, which is supported by the presence of overlapping continuous dictyonal framework rays. Furthermore, these genera are not closely related to Auloplacidae and Aulocalycidae (as in Fig. 5), with which they share several similarities in framework construction, implying that these characters evolved convergently in the three families. Tretodictyidae *s. str.* (see section Phylogenies inferred from molecular data) also includes *Anomochone* and *Sclerothamnopsis*, and the remaining three tretodictyid genera group together in a clade with Uncinateridae. Thus, this phylogeny appears more parsimonious in suggesting a diphyletic instead of a triphyletic (as in Fig. 5) origin of Tretodictyidae. Congruent with the morphology-only analysis, Aphrocallistidae, Auloplacidae, and Farreidae are reconstructed as monophyletic. Furthermore, *Laocoetis* and *Stereochlamis*, the only known extant genera of the paleontologically important Craticulariidae and Cribrospongiidae (cf. [7]) are reconstructed as sister groups, which is supported by the presence of a so-called diplorhysial framework channelization unique to these two families. As in Fig. 6 and 6, Euretidae is clearly polyphyletic, which is not surprising given that this family constitutes a “waste-bin taxon” for all genera that do not fit into any of the other families (see [17]). Morphological support for many parts of the topology within Sceptrulophora is not clear-cut, especially concerning the deepest nodes. Clearly, many placements of the unsequenced genera in Fig. 9, especially the euretids, can only serve as initial working hypotheses that await to be tested with molecular data.

***Lyssacinosida*** **s. nov**. Higher-level relationships within Lyssacinosida *s. nov.* are congruent with the molecular phylogenies (Fig. 7, Additional file 23: Figure S9), with Lyssacinosida *inc. sed.* (*Clathrochone*, *Hyaloplacoida*) and Leucopsacidae more closely related to Rossellidae than to Euplectellidae. As discussed in [14], morphological support for this branching order remains unclear. Aulocalycidae is here reconstructed as sister to Leucopsacidae + Rossellidae, in contrast to the ML phylogenies inferred from the molecular supermatrix and the total-evidence matrix restricted to sequenced genera (Fig. 7, Additional file 25: Figure S11), where Aulocalycidae (*Euryplegma*) and Leucopsacidae were reconstructed as sister groups (albeit with weak support). Molecular data from additional aulocalycids will be required to disambiguate between these two hypotheses. However, the latter one is intriguing because Leucopsacidae have choanosomal megascleres exclusively as hexactins, as is the case for all dictyonal taxa as well, whereas the majority of lyssacine hexasterophorans have choanosomal spicules dominated by diactins (see Additional file 14). That is, hexactine choanosomalia are a prerequisite for developing a dictyonal framework, and the evolution of this character in a hypothetical LCA of Leucopsacidae and Aulocalycidae could have provided a pre-adaption that opened the way for the convergent evolution of dictyonal frameworks in the latter. Interestingly, aulocalycid frameworks are characterized by intensive synapticular bridging (see Fig. 2d). Spicule fusion by synapticular bridging is widespread among Lyssacinosida (see Fig. 2h), but otherwise only rarely found (in the “euretids” *Heterorete*, *Tretochone*, and *Pleurochorium*), thus providing some morphological support for inclusion of Aulocalycidae in Lyssacinosida. Besides from that, morphological evidence for this new placement of Aulocalycidae remains scarce, except perhaps for the presence of stauractins and diactins in *Aulocalyx*, spicules that are common in Lyssacinosida but rare in other taxa.

Detailed accounts of morphological support for monophyly of Lyssacinosida, Euplectellidae, *Clathrochone* + *Hyaloplacoida*, Rossellidae, Leucopsacidae, and Aulocalycidae, as well as internal relationships of the latter two taxa, can be found in the first section of the supplementary discussion in Additional file 14.

***Euplectellidae***. Congruent with Fig. 6, the total-evidence analysis recovered a clade of all genera with the iconical *Euplectella*-like body shape (Fig. 1f), the “venus-flower basket” or VFB clade (which is similar but not identical to Euplectellidae *s. str.* of Mehl [93]). Successive sister groups to the VFB clade include genera with a body shape that can be interpreted as primitive to or derived from a venus-flower basket, so we refer to this larger assemblage as “VFB *sensu lato*”. In contrast to Fig. 6, all genera with discoplumicomes (Fig. 3c) group together, so the total-evidence phylogeny is more parsimonious in suggesting only a single origin for this complex type of microsclere. The “discoplumicome clade” contains members of all three currently accepted subfamilies [95], further demonstrating the artificial nature of this division, which is based on mode of attachment to the substrate, a rather homoplastic character. The genera *Walteria* and *Dictyocalyx* (Corbitellinae) together are sister to the discoplumicome clade, but we suspect that this is a misplacement and they are rather related to the VFB *s. l.* clade (see Additional file 14). Sister to all the above groups is a clade containing the majority of Bolosominae (which we call Bolosominae *sensu stricto*), the stalked euplectellids (Fig. 1g). This result is similar to the morphology-only analysis (“core bolosomins” as sister to the remaining euplectellids; Fig. 6) but is more parsimonious in that it includes the new genus from off Hawaii, which is very similar to *Rhabdopectella* in spiculation (MD, pers. obs.), and excludes only the discoplumicome-bearing genera *Saccocalyx* and *Caulocalyx*. We will not yet make any official classificatory changes on the basis of these findings, but we hope that support for this subdivision will solidify with increased sampling of euplectellid genera for molecular phylogenetics.

***Rossellidae***. The total-evidence topology of Rossellidae differs substantially from that inferred from morphological data alone. In accordance with the molecular results (Fig. 7, Additional file 23: Figure S9), most genera of Rossellidae fall in one of two major clades, Lanuginellinae (*sensu* [83]; i.e., including *Caulophacus* and *Caulophacella*), and a clade with mostly microdiscohexaster (Fig. 3g)-bearing genera (a division that was basically already recognized by Mehl [93]). Only the unsequenced *Vitrollula* and *Aphorme* seem to disrupt this simple picture: *Vitrollula* (with microdiscohexasters) is resolved as sister to Lanuginellinae, and *Aphorme* (without microdiscohexasters) sister to the remaining genera (note that microdiscohexasters are also, likely secondarily, absent in *Bathydorus* and *Trichasterina*). A sister-group relationship of *Vitrollula* and Lanuginellinae is supported by the presence of a significant number of hexactins supplementing the choanosomal diactins (secondarily lost in *Doconesthes* + *Mellonympha*). In contrast, the placement of *Aphorme* finds no obvious support from any morphological characters. Pending resolution of the positions of *Vitrollula* and *Aphorme* with molecular data, it would be tempting to recognize the microdiscohexaster clade as Rossellinae *s. nov.*, because this subfamily is currently purely negatively defined [96]. A subdivision of Rossellidae into Rossellinae *s. nov.* and Lanuginellinae *sensu* [83] would appear to be a natural choice. However, the recent resurrection of Acanthascinae [51] greatly complicates matters because this taxon appears to be an ingroup of Rossellinae *s. nov.* A close relationship between *Acanthascus*, *Rhabdocalyptus*, and *Staurocalyptus* is unambiguously supported by the exclusive presence of discoctasters (Fig. 3e) in these three taxa, but given the reassignment of subfamily rank to this group [51], a natural classification of Rossellidae that is free of paraphyletic taxa seems to be out of reach for now.

### Maximum-likelihood ancestral state reconstruction

**Fig. 11:**
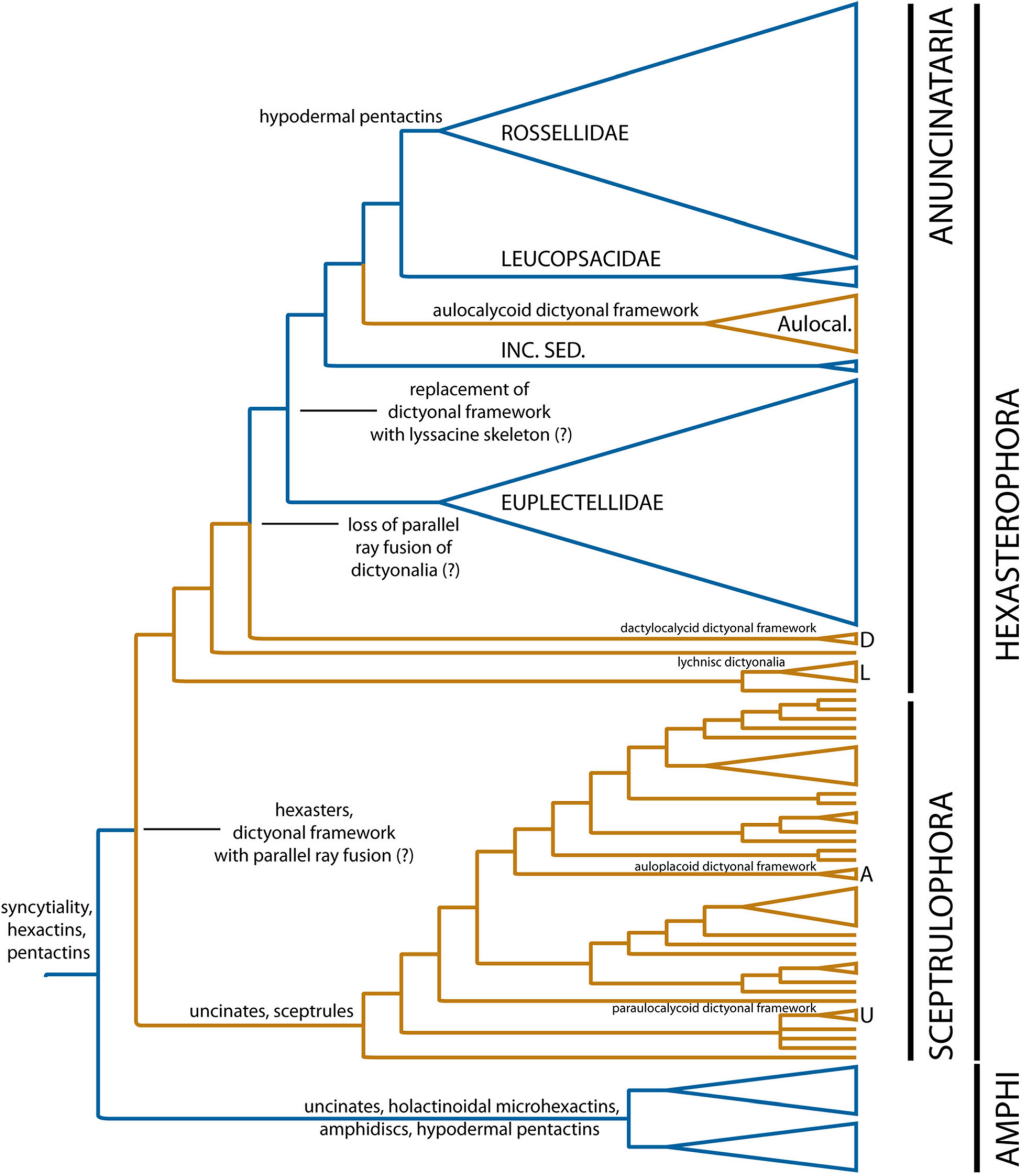
Simplified phylogeny of Hexactinellida (after Figs. 8, 9 and 10) showing tentative scenario of the evolution of selected characters. Blue branches represent lyssacine taxa, brown branches represent dictyonal taxa. Note that the reconstruction of some characters is sensitive to alternative topologies and/or ancestral state reconstruction model (see text for details). Amphi, Amphidiscophora; A, Auloplacidae; Aulocal., Aulocalycidae; D, Dactylocalycidae; INC. SED., Lyssacinosida *incertae sedis*; L, Lychniscosida; U, Uncinateridae

For clarity, we present a summary of our conclusions in Fig. 11. Besides from hexactins and syncytial tissue organization, which are the defining autapomorphies of Hexactinellida and were not included in the matrices as they are parsimony-uninformative for ingroup relationships, we inferred that pentactine (five-rayed) megascleres (proportional likelihood [pl] under Mk1/aMk2 = 1.00/0.90) and possibly a dermal (outer tissue layer) skeleton dominated by these spicules (pl = 0.80/0.68) were present in the LCA of Hexactinellida. Pentactine megascleres could also have been the dominant spicule type of the atrial (inner tissue layer) skeleton, but this was only marginally supported (0.54/0.56). For the choanosomal (middle tissue layer) megasclere composition no ancestral type could be found, although hexactins and/or pentactins would be obvious candidates. The presence of uncinates in the LCA of Hexactinellida was not supported (0.23/0.30), suggesting that it is more likely that these spicules evolved convergently in Amphidiscophora and Sceptrulophora. Interestingly, the presence of microhexactins (oxyhexactins; small hexactins with pointed tips and no secondary rays) – the most basic type of microsclere – was also not supported (0.49/0.48). This inference is in line with the observation that these spicules are holactins (proteinaceous axial filaments extending to the ray tips) in Amphidiscophora, but heteractins (axial filaments not extending to the ray tips) in Hexasterophora [2]. Thus, hexasterophoran oxyhexactins could have evolved independently via reduction of secondary rays of hexasters, leaving only a single ray per primary ray. Because other microsclere types (hexasters, amphidiscs) are mutually exclusive in the two subclasses, this raises the possibility that microscleres are not homologous in Amphidiscophora and Hexasterophora.

The ancestral mode of attachment to the substrate was reconstructed as basiphytous (0.82/0.82) for Hexactinellida. However, this result has to be viewed with caution because Hexasterophora, where the vast majority of genera uses this mode, are disproportionately more genus-rich than Amphidiscophora, where this mode never occurs. Thus, in a hypothetical scenario where both subclasses had the same number of genera, the ancestral state would probably be highly ambiguous. Similar arguments can be made for the ancestral hexactinellid body plan reconstructed by the methods employed here: this was marginally supported as dictyonal (0.52/0.59). However, we consider it unlikely that the LCA of Hexactinellida was a dictyonal sponge – we rather suspect that amphidiscophorans retained an ancestral unfused skeleton, because in this group evidence for spicule fusion is entirely lacking [2] and there is no reason to believe that fused skeletons are an ancestral feature of siliceous sponges (Demospongiae + Hexactinellida).

The LCA of Amphidiscophora was reconstructed as a lyssacine sponge with lophophytous attachment to the substrate (1.00/1.00), dermal skeleton with small megascleres supported by large hypodermal pentactins (1.00/1.00; convergent to Rossellidae), dermal skeleton dominated by pentactins (0.99/0.98), atrial skeleton dominated by pentactins (0.96/0.96), presence of pinular hexactins (0.97/0.93) and pentactins (1.00/1.00; convergent to Lanuginellinae), stauractins (0.89/0.91), uncinates (0.99/1.00), and microscleres as oxyhexactins (0.92/0.92) and amphidiscs of all three size classes (0.99 to 1.00). As these characters occur in almost all genera, this reconstruction is somewhat trivial. Regarding the choanosomal skeletal composition, however, only the presence of hexactins among mixed choanosomalia gained some support under the aMk2 model (0.87) – we hypothesize that the different compositions defining the three families [97] each evolved independently from a simple hexactin-dominated ground state. The aMk2 model analyses also reconstructed additional ancestral states for the LCA of Amphidiscophora that were not supported by the simpler Mk1 model: hypoatrial pentactins (0.69), amphidiscs with additional rays (0.99), oxyoidal (acute-tipped) or clavate (club-tipped) monactin/diactin attachment spicules (0.93), and toothed anchorate attachment spicules (1.00). Regarding the amphidiscs, this result implies that the six-rayed “hexadiscs” found in some genera in addition to the more common two-rayed regular amphidiscs (Fig. 2i) are plesiomorphic remnants and that the latter evolved from the former by ray reduction. The inference about the anchorate attachment spicules is sensitive to the position of *Monorhaphis* and disappears when this genus is placed as sister to Hyalonematidae + Pheronematidae (as in Additional file 27: Figure S14). This was not the case for the oxyoidal attachment spicules – these spicules are unknown from Hyalonematidae, so this result implies that they were secondarily lost in this family.

The LCA of Hexasterophora was reconstructed as a basiphytous (0.98/0.97), dictyonal (0.76/0.84) sponge with parallel ray fusion of dictyonal hexactins (0.66/0.94). As already discussed above, this implies that a lyssacine body plan “re-evolved” within this subclass, followed by independent evolution of dictyonal skeletons in Aulocalycidae. However, this reconstruction was somewhat sensitive to model choice and the topology at the base of Anuncinataria: when the position of *Myliusia* was changed to sister of *Heterorete* + Lychniscosida or to sister of Dactylocalycidae, or when all four taxa were constrained to form a clade, the likelihood of the presence of a dictyonal body plan in the LCA of Hexasterophora dropped below 0.45 under both models (range 0.13–0.43). However, the character “dictyonal bauplan” is an oversimplification (and was thus not used for phylogeny reconstruction), so “parallel ray fusion of dictyonal hexactins” is a more meaningful character to look at. The presence of this construction type in the LCA of Hexasterophora was also not supported under the three alternative topologies when the Mk1 model was used (range 0.26–0.48); however, the aMk2 model supported its presence by pl of 0.75–0.83. These observations demonstrate that a robust resolution of this part of the topology, especially by including sequence data from Lychniscosida and *Myliusia*, will be required to more confidently reconstruct the evolution of non-sceptrulophoran dictyonal frameworks. Furthermore, the distinct construction type of dactylocalycid frameworks (Fig. 2c) suggest that they might have evolved convergently (see Additional file 14), regardless of phylogenetic considerations. On the other hand, a possible link between dictyonal and lyssacine hexasterophorans are the so-called basidictyonal frameworks occuring in both groups, which are structures of fused spicules involved in the attachment of basiphytous species to the substratum [2]. Interestingly, basidictyonal spicules connect by tip-to-tip fusion [2], which is rare in sceptrulophoran choanosomal frameworks but very common in those of the sister-group of Lyssacinosida, the Dactylocalycidae. Thus, lyssacine hexasterophorans might have evolved by suppression of further development of their basidictyonal spicules into fully-fledged choanosomal dictyonal frameworks, instead re-deploying an ancient genetic program that instructs development of unfused choanosomalia (but see also discussion in [15]). Evolutionary developmental (evo-devo) studies would be of great help in answering these questions, but are likely too difficult to implement in absence of easily manipulatable hexactinellid model systems from different relevant taxa.

With respect to loose spiculation, the LCA of Hexasterophora likely retained pentactine dermal (0.82/0.68) and possibly atrial (0.54/0.56) megascleres from the ground pattern of Hexactinellida. Microscleres of course included hexasters (0.99/0.98), the defining apomorphy of this subclass, most likely in the form of oxy- (Fig. 2j) (0.59/0.71) and disco- (Fig. 3) hexasters (0.92/0.74). Presence of oxyhexactins in the LCA of Hexasterophora was poorly supported (0.48/0.47), suggesting that these spicules might have repeatedly evolved convergently, which appears plausible because under the scenario hypothesized above only loss of some secondary rays is required to evolve these spicules from hexasters.

Comparison of the two different ancestral state reconstruction models further revealed that the Mk1 model frequently supported multiple independent origins of a character towards the tips of a clade, whereas the aMk2 model preferred a single origin at the clade’s root, followed by multiple losses. For example, synapticular spicule fusion evolved eight times in Lyssacinosida according to the Mk1 model, whereas the aMk2 model inferred a single origin in the LCA of this group. Further examples include skeletal channelization in Sceptrulophora, choanosomal skeletons dominated by diactins in Lyssacinosida, graphiocomes and floricomes (Fig. 3b, l) in Euplectellidae, and strobiloplumicomes (Fig. 3d) in Lanuginellinae (see also results on Amphidiscophora above). This suggests that the simplifying assumption made by the Mk1 model that gains and losses are equally likely can frequently lead to an inflated estimate of convergent evolution. However, it has to be evaluated on a case-by-case basis whether loss or gain of a particular character is more likely. For instance, floricomes and strobiloplumicomes are quite complex spicules and therefore postulating convergent evolution for this character seems unparsimonious. On the other hand, skeletal channelization of dictyonal frameworks is probably something that is easily evolved, as can be seen for example by the occurrence of channels (epirhyses) in ontogenetically older specimens of the usually unchannelized farreids (e.g., [46]), and also by the multitude of different channelization types (see Additional file 4) that are indicative of convergent evolution. Therefore, although the aMk2 model generally appears to be more realistic by accounting for differences between gain and loss rates, it is important also to conduct analyses under the simpler Mk1 model and compare the results in the light of biological plausibility (see also [98]).

## Conclusions

In this study, we have increased the taxon sampling for molecular systematics of Hexactinellida by 15 species, 12 genera, three families, and one order. One major finding was that the order Aulocalycoida is polyphyletic because its two constituent families (Aulocalycidae and Uncinateridae) are resolved as ingroups of Lyssacinosida and Sceptrulophora, respectively. Furthermore, the sceptrule- and uncinate lacking dictyonal genera *Heterorete* (formerly Euretidae) and *Dactylocalyx* (Dactylocalycidae) were resolved as more closely related to Lyssacinosida than to Sceptrulophora, which further demonstrates the artificial nature of Hexactinosida. Consequently, we abolish Aulocalycoida and Hexactinosida, elevate Sceptrulophora from suborder to order, and emend diagnosis and scope of Lyssacinosida to include Aulocalycidae. These updates are timely and bring the Linnean classification of glass sponges in closer agreement with their phylogeny, similar to what was recently proposed for Demospongiae [99].

We further compiled morphological character matrices including all extant genera of Amphidiscophora and Hexasterophora and analyzed these alone and in combination with the molecular data. We compared MP, ML, and Bayesian approaches, as well as “morphology-based phylogenetic binning” [22] and found that MP consistently outperformed the other methods in terms of congruence with well-founded taxonomic and phylogenetic hypotheses. Bayesian analyses performed second best, whereas ML and binning gave largely dubious results. Phylogenies based only on morphological data were partly congruent with the molecular tree (e.g., paraphyly of Hexactinosida, monophyly of many families), but also conflicted in many areas (e.g., monophyletic Aulocalycoida nested within Sceptrulophora). The total-evidence trees were largely congruent with the molecular phylogeny and suggest that the major division of Hexasterophora is not between lyssacine and dictyonal taxa, but instead between taxa with and without sceptrules and uncinates, i.e., between Sceptrulophora and a clade we call Anuncinataria. Besides Lyssacinosida (including Aulocalycidae), Dactylocalycidae, and *Heterorete*, Anuncinataria also includes *Myliusia* (formerly Euretidae) and Lychniscosida, a species-poor relict group that was highly diverse in the Jurassic and Cretaceous. Inclusion of sequence data from the latter two taxa will be crucial to further test the monophyly of Anuncinataria. In general, placement of the unsequenced genera in our total-evidence phylogeny should not be taken as the last word but as a starting point; these are working hypotheses that need to be further tested by filling the gaps in the molecular dataset. Also, the morphological character matrices should not be viewed as static, but as a resource that is subject to constant revision.

Character mapping and ML ancestral state reconstruction (ASR) on the total-evidence tree allowed us to gain deeper insights into the evolution of skeletal structures in Hexactinellida. Our results suggest that evolution of the dictyonal body plan was more complex than previously thought. Besides from the obvious implication that the dictyonal skeletons of Aulocalycidae evolved convergently from a lyssacine condition and that the peculiar construction types of Uncinateridae and Auloplacidae evolved independently from a more regular type, we found that dictyonal skeletons with parallel ray fusion might have been present in the ground pattern of Hexasterophora and got secondarily lost in the stem lineage of Lyssacinosida. That is, the lyssacine condition in Lyssacinosida might represent a case of evolutionary reversal to an ancestral body plan, the genetic program for which was inherited from the last common ancestor of Hexactinellida. However, this inference was sensitive to ASR model choice and the branching pattern at the base of Anuncinataria, so the possibility that dictyonal frameworks evolved once or multiple times convergently in early-branching anuncinatarians (e.g., Dactylocalycidae) cannot be ruled out until the phylogenetic relationships of these taxa are better resolved.

Concerning loose spiculation, we also found – not unexpectedly – high levels of homoplasy. The degree to which this is due to multiple convergent origins or multiple losses of spicule types depends somewhat on the assumptions of the ASR model used, and has to be evaluated on a case-by-case basis of individual characters. However, it appears that hexactinellids, and sponges in general, are able to retain the genetic instructions to produce certain spicule types over long evolutionary time, even if they are not expressed in the phenotype. For example, discasters (Fig. 3i) and sigmato- or drepanocomes (Fig. 3k) are restricted to Lyssacinosida but within this group are only found in 14 and 12 genera, respectively, scattered across three families. That is, it is the ability to produce these spicules that can be interpreted as an apomorphy of Lyssacinosida, not their actual phenotypic expression. The importance of this phenomenon, which has been called “cryptotypic property” [100], in Hexactinellida was already pointed out by Mehl [93]. Furthermore, Maldonado et al. [101] showed that sponges can be forced to produce spicule types not normally found in a given species just by altering the silica concentration of sea water. Although these phenomena are largely ignored by sponge taxonomists, at least in Hexactinellida the problem of homoplasy seems less severe than in other sponge classes (cf. [99, 102]). Integrating morphology and molecular sequence data has great potential to inform us about the evolution of this fascinating group of animals.

## Appendix 1

**Table 2:** Taxonomic overview of hexactinellid genera

Subclass: Genus	Authority	#spp.	Subfamily	Order: Family
Amphidiscophora				Amphidiscosida
*Hyalonema*	Gray, 1832	120		Hyalonematidae
*Chalaronema*	Ijima, 1927	1		Hyalonematidae
*Compsocalyx*	Schulze, 1904	1		Hyalonematidae
*Lophophysema*	Schulze, 1900	4		Hyalonematidae
*Tabachnickia*	Özdikmen, 2010	1		Hyalonematidae
*Monorhaphis*	Schulze, 1904	1		Monorhaphididae
*Pheronema*	Leidy, 1868	18		Pheronematidae
*Platylistrum*	Schulze, 1904	1		Pheronematidae
*Poliopogon*	Thomson, 1878	6		Pheronematidae
*Schulzeviella*	Tabachnick, 1990	1		Pheronematidae
*Semperella*	Gray, 1868	11		Pheronematidae
*Sericolophus*	Ijima, 1901	5		Pheronematidae
Hexasterophora				Lyssacinosida
*Euplectella*	Owen, 1841	17	Euplectellinae	Euplectellidae
*Acoelocalyx*	Topsent, 1910	1	Euplectellinae	Euplectellidae
*Chaunangium*	Schulze, 1904	1	Euplectellinae	Euplectellidae
*Docosaccus*	Topsent, 1910	2	Euplectellinae	Euplectellidae
*Holascus*	Schulze, 1886	15	Euplectellinae	Euplectellidae
*Malacosaccus*	Schulze, 1886	7	Euplectellinae	Euplectellidae
*Placopegma*	Schulze, 1895	2	Euplectellinae	Euplectellidae
*Bolosoma*	Ijima, 1904	8	Bolosominae	Euplectellidae
*Amphidiscella*	Tabachnick & Lévi, 1997	4	Bolosominae	Euplectellidae
*Caulocalyx*	Schulze, 1886	1	Bolosominae	Euplectellidae
*Hyalostylus*	Schulze, 1886	2	Bolosominae	Euplectellidae
*Neocaledoniella*	Tabachnick & Lévi, 2004	1	Bolosominae	Euplectellidae
*Rhabdopectella* ^a^	Schmidt, 1880	1	Bolosominae	Euplectellidae
*Saccocalyx*	Schulze, 1896	3	Bolosominae	Euplectellidae
*Trachycaulus*	Schulze, 1886	1	Bolosominae	Euplectellidae
*Vityaziella*	Tabachnick & Lévi, 1997	1	Bolosominae	Euplectellidae
*Corbitella*	Gray, 1867	4	Corbitellinae	Euplectellidae
*Atlantisella*	Tabachnick, 2002	1	Corbitellinae	Euplectellidae
*Dictyaulus*	Schulze, 1896	4	Corbitellinae	Euplectellidae
*Dictyocalyx*	Schulze, 1886	2	Corbitellinae	Euplectellidae
*Hertwigia*	Schmidt, 1880	1	Corbitellinae	Euplectellidae
*Heterotella*	Gray, 1867	4	Corbitellinae	Euplectellidae
*Ijimaiella*	Tabachnick, 2002	1	Corbitellinae	Euplectellidae
*Pseudoplectella*	Tabachnick, 1990	1	Corbitellinae	Euplectellidae
*Regadrella*	Schmidt, 1880	8	Corbitellinae	Euplectellidae
*Walteria*	Schulze, 1886	2	Corbitellinae	Euplectellidae
*Leucopsacus*	Ijima, 1898	4		Leucopsacidae
*Oopsacas*	Topsent, 1927	4		Leucopsacidae
*Chaunoplectella*	Ijima, 1896	1		Leucopsacidae
*Rossella*	Carter, 1872	20	Rossellinae	Rossellidae
*Anoxycalyx*	Kirkpatrick, 1907	3	Rossellinae	Rossellidae
*Aphorme*	Schulze, 1899	1	Rossellinae	Rossellidae
*Asconema*	Kent, 1870	5	Rossellinae	Rossellidae
*Aulosaccus*	Ijima, 1896	5	Rossellinae	Rossellidae
*Bathydorus*	Schulze, 1886	7	Rossellinae	Rossellidae
*Crateromorpha*	Gray in Carter, 1872	15	Rossellinae	Rossellidae
*Hyalascus*	Ijima, 1896	9	Rossellinae	Rossellidae
*Nodastrella*	Dohrmann, Göcke, Reed & Janussen, 2012	2	Rossellinae	Rossellidae
*Schaudinnia*	Schulze, 1900	1	Rossellinae	Rossellidae
*Scyphidium*	Schulze, 1900	8	Rossellinae	Rossellidae
*Symplectella*	Dendy, 1924	1	Rossellinae	Rossellidae
*Trichasterina*	Schulze, 1900	2	Rossellinae	Rossellidae
*Vazella*	Gray, 1870	1	Rossellinae	Rossellidae
*Vitrollula*	Ijima, 1898	1	Rossellinae	Rossellidae
*Acanthascus*	Schulze, 1886	7	Acanthascinae	Rossellidae
*Rhabdocalyptus*	Schulze, 1886	18	Acanthascinae	Rossellidae
*Staurocalyptus*	Ijima, 1897	17	Acanthascinae	Rossellidae
*Lanuginella*	Schmidt, 1870	1	Lanuginellinae	Rossellidae
*Calycosoma*	Schulze, 1899	1	Lanuginellinae	Rossellidae
*Caulophacella* ^b^	Lendenfeld, 1915	1	Lanuginellinae	Rossellidae
*Caulophacus*	Schulze, 1886	26	Lanuginellinae	Rossellidae
*Doconesthes*	Topsent, 1928	2	Lanuginellinae	Rossellidae
*Lanugonychia*	Lendenfeld, 1915	1	Lanuginellinae	Rossellidae
*Lophocalyx*	Schulze, 1887	12	Lanuginellinae	Rossellidae
*Mellonympha*	Schulze, 1897	1	Lanuginellinae	Rossellidae
*Sympagella*	Schmidt, 1870	9	Lanuginellinae	Rossellidae
*Clathrochone*	Tabachnick, 2002	1		*incertae sedis*
*Hyaloplacoida*	Tabachnick, 1989	1		*incertae sedis*
				Hexactinosida (Sceptrulophora)
*Aphrocallistes*	Gray, 1858	2		Aphrocallistidae
*Heterochone*	Ijima, 1927	4		Aphrocallistidae
*Auloplax*	Schulze, 1904	4		Auloplacidae
*Dictyoplax*	Reiswig & Dohrmann, 2014	1		Auloplacidae
*Laocoetis*	Pomel, 1872	1		Craticulariidae
*Stereochlamis*	Schrammen, 1912	1		Cribrospongiidae
*Eurete*	Semper, 1868	12	Euretinae	Euretidae
*Calyptorete*	Okada, 1925	1	Euretinae	Euretidae
*Conorete*	Ijima, 1927	5	Euretinae	Euretidae
*Endorete*	Topsent, 1928	1	Euretinae	Euretidae
*Gymnorete*	Ijima, 1927	3	Euretinae	Euretidae
*Heterorete*	Dendy, 1916	1	Euretinae	Euretidae
*Homoieurete*	Reiswig & Kelly, 2011	1	Euretinae	Euretidae
*Lefroyella*	Thomson, 1878	2	Euretinae	Euretidae
*Pararete*	Ijima, 1927	7	Euretinae	Euretidae
*Pityrete*	Topsent, 1928	1	Euretinae	Euretidae
*Chonelasma*	Schulze, 1886	10	Chonelasmatinae	Euretidae
*Bathyxiphus*	Schulze, 1899	1	Chonelasmatinae	Euretidae
*Myliusia*	Gray, 1859	4	Chonelasmatinae	Euretidae
*Periphragella*	Marshall, 1875	6	Chonelasmatinae	Euretidae
*Pinulasma*	Reiswig & Stone, 2013	1	Chonelasmatinae	Euretidae
*Pleurochorium*	Schrammen, 1912	2	Chonelasmatinae	Euretidae
*Tretochone*	Reid, 1958	1	Chonelasmatinae	Euretidae
*Verrucocoeloidea*	Reid, 1969	2	Chonelasmatinae	Euretidae
*Farrea*	Bowerbank, 1862	30		Farreidae
*Asceptrulum*	Duplessis & Reiswig, 2004	1		Farreidae
*Aspidoscopulia*	Reiswig, 2002	5		Farreidae
*Claviscopulia*	Schulze, 1899	1		Farreidae
*Lonchiphora*	Ijima, 1927	2		Farreidae
*Fieldingia*	Kent, 1870	2		Fieldingiidae
*Tretodictyum*	Schulze, 1886	7		Tretodictyidae
*Anomochone*	Ijima, 1927	3		Tretodictyidae
*Cyrtaulon*	Schulze, 1886	2		Tretodictyidae
*Hexactinella*	Carter, 1885	14		Tretodictyidae
*Psilocalyx*	Ijima, 1927	1		Tretodictyidae
*Sclerothamnopsis*	Wilson, 1904	2		Tretodictyidae
*Sclerothamnus*	Marshall, 1875	1		Tretodictyidae
*Tretocalyx*	Schulze, 1901	1		Tretodictyidae
*Sarostegia*	Topsent, 1904	1		*incertae sedis*
				Hexactinosida
*Dactylocalyx*	Stutchbury, 1841	2		Dactylocalycidae
*Iphiteon*	Bowerbank, 1869	1		Dactylocalycidae
				Aulocalycoida
*Aulocalyx*	Schulze, 1886	3	Aulocalycinae	Aulocalycidae
*Euryplegma*	Schulze, 1886	1	Aulocalycinae	Aulocalycidae
*Ijimadictyum*	Mehl, 1992	1	Aulocalycinae	Aulocalycidae
*Indiella*	Sautya, Tabachnick & Ingole, 2011	1	Aulocalycinae	Aulocalycidae
*Leioplegma*	Reiswig & Tsurumi, 1996	1	Aulocalycinae	Aulocalycidae
*Rhabdodictyum*	Schmidt, 1880	1	Aulocalycinae	Aulocalycidae
*Cyathella*	Schmidt, 1880	1	Cyathellinae	Aulocalycidae
*Uncinatera*	Topsent, 1901	1		Uncinateridae
*Tretopleura*	Ijima, 1927	2		Uncinateridae
				Lychniscosida
*Neoaulocystis*	Zhuravleva, 1962	4		Aulocystidae
*Lychnocystis*	Reiswig, 2002	1		Aulocystidae
*Scleroplegma*	Schmidt, 1880	2		Diapleuridae

^a^Currently classified in Corbitellinae, but see Table 1: footnote c of [3]

^b^This genus was recently reclassified as a subgenus of *Caulophacus* ([83]; following [26]). However, molecular data do not support inclusion of *Caulophacella tenuis* in *Caulophacus* [3, 16]. Therefore, we here retained *Caulophacella* as a terminal taxon

## Appendix 2

### Changes to Linnean classification, including revised diagnoses

#### Summary

The subclass Hexasterophora Schmidt, 1870 now contains the following three orders: Sceptrulophora, Lyssacinosida, and Lychniscosida. The former order Hexactinosida is abolished by recognition of Sceptrulophora and Dactylocalycidae as unrelated taxa. The former order Aulocalycoida is abolished by moving its two constituent families to Sceptrulophora and Lyssacinosida, respectively. One family – Dactylocalycidae with the two genera *Dactylocalyx* and *Iphiteon* – and five further genera – *Heterorete*, *Myliusia* (formerly Euretidae), *Deanea*, *Diaretula*, and *Hyalocaulus* (formerly Hexactinosida *inc. sed.*) – cannot presently be assigned to any of the orders and are treated as Hexasterophora *inc. sed.*. Sceptrulophora contains nine families – Aphrocallistidae, Auloplacidae, Craticulariidae, Cribrospongiidae, Euretidae, Farreidae, Fieldingiidae, Tretodictyidae, and Uncinateridae (formerly Aulocalycoida); three genera are treated as Sceptrulophora *inc. sed.*: *Sarostegia* (cf. [17]), *Cyrtaulon* (formerly Tretodictyidae), and *Homoieurete* (formerly Euretidae). Lyssacinosida contains four families: Euplectellidae, Leucopsacidae, Rossellidae, and Aulocalycidae (formerly Aulocalycoida); two genera (*Clathrochone*, *Hyaloplacoida*) are treated as Lyssacinosida *inc. sed.*. Lychniscosida contains two families (Aulocystidae, Diapleuridae).

SCEPTRULOPHORA MEHL, 1992 **ord. nov.**

*Diagnosis (emended from Dohrmann* et al. [19]*)*: Dictyonal Hexactinellida with uncinates; sceptrules usually present but can be missing in rare cases. Body shape is highly variable from branching and anastomosing tubes to cup, funnel, or blade forms. Dictyonal skeleton mostly euretoid, but farreoid, auloplacoid, or paraulocalycoid patterns also occur. Channelization of dictyonal framework as epi- and/or aporhyses, schizorhyses, diarhyses, diplorhyses, amararhyses, or absent. Dermalia and atrialia usually pentactins, sometimes hexactins or absent. Microscleres oxy- and/or discohexasters and their derivatives; onycho- and tylo-tipped forms might also occur.

*Scope*: The following families are included in Sceptrulophora: Aphrocallistidae Gray, 1867, Auloplacidae Schrammen, 1912, Craticulariidae Rauff, 1893, Cribrospongiidae Roemer, 1864 (but see remarks in [19]), Euretidae Zittel, 1877, Farreidae Gray, 1872, Fieldingiidae Tabachnick & Janussen, 2004, Tretodictyidae Schulze, 1886, and Uncinateridae Reiswig, 2002. Uncinateridae (formerly Aulocalycoida Tabachnick & Reiswig, 2000) is here included on the basis of molecular evidence (e.g., Fig. 7) and the presence of uncinates (as well as sceptrules in *Tretopleura* Ijima, 1927).

*Remarks*: Sceptrules are absent from *Asceptrulum* Duplessis & Reiswig, 2004 (Farreidae) and have not been confirmed so far from the poorly known *Uncinatera* Topsent, 1901 (Uncinateridae), both of which are monospecific genera. However, the presence of uncinates in these species clearly supports their inclusion in Sceptrulophora. The genera *Heterorete* Dendy, 1916 and *Myliusia* Gray, 1859 (formerly Euretidae) have neither uncinates nor sceptrules, and are excluded from Sceptrulophora (see below). Uncinateridae Reiswig, 2002 (*Uncinatera* and *Tretopleura*) have a paraulocalycoid framework construction; the term “auloplacoid” is introduced here to distinguish the skeletal architecture of Auloplacidae Schrammen, 1912 from the prevailing euretoid pattern (see [17]). Elevation of Sceptrulophora from subordinal [19] to ordinal status follows from abolishment of Hexactinosida Schrammen, 1912 (see below). Because the concept of the new order is the same as for the clade name established by Mehl, we prefer to leave the authority with this author.

EURETIDAE ZITTEL, 1877

*Diagnosis (emended from Dohrmann* et al. [19]*)*: Sceptrulophora with body form either of branching and/or anastomosing tubes, or cup-funnel formed of a ring of tubes, or of a single tube, or of a single-wall funnel with or without lateral oscula extended on marginal tubes, or blade form; dictyonal meshes mainly rectangular or triangular or irregular; meshes usually equal-sided but elongate prismatic mesh series with transverse lamellae developed in some species; dictyonal strands, if developed, orientated longitudinally; with or without dictyonal cortices composed of primary or secondary dictyonalia; dermalia and atrialia are commonly pentactins or pinular hexactins with rays of approximately equal length, or both forms lacking; scopules (which might also be represented by sarules) and uncinates present; microscleres occur as oxyhexasters and/or discohexasters.

*Scope*: As of May 2016, Euretidae was comprised of 18 genera [5]. Here, we remove the sceptrule- and uncinate-lacking *Heterorete* and *Myliusia* and re-classify them provisionally as Hexasterophora *inc. sed.* because phylogenetic analyses (e.g., Figs. 5, 7 and 10) clearly place them outside of Sceptrulophora but their exact phylogenetic position (and therefore ordinal assignment) remains to be determined (see main text and Additional file 14 for further details). *Homoieurete* Reiswig & Kelly, 2011 is also removed from Euretidae, because molecular evidence (e.g., Fig. 7) strongly suggests that it is unrelated to the other three euretids with available DNA sequence data. Following suggestions of Reiswig and Dohrmann [17], we thus provisionally re-classify *Homoieurete* as Sceptrulophora *inc. sed.*

*Remarks*: The emended diagnosis reflects the fact that Euretidae no longer contains taxa without sceptrules and uncinates. However, even after removal of the above-mentioned genera, Euretidae clearly constitutes a “waste-bin” taxon that lumps together all sceptrulophoran genera not assignable to any of the other families (see [17]). Our phylogenetic analyses (e.g., Figs. 5 and 9) suggest that these genera are highly interspersed within Sceptrulophora, some being related to each other and others more closely related to different families. If at all, this taxonomic challenge can only be resolved by molecular data. For the time being, we retain Euretidae within the Linnean system while acknowledging its artificial nature.

TRETODICTYIDAE SCHULZE, 1886

*Diagnosis (emended from Reiswig and Kelly* [44]*)*: Sceptrulophora with body form varying from branching and anastomosing solid cylinders to branching and anastomosing tubes to funnel, cup, and irregular globular forms; with three-dimensional, small-meshed, euretoid dictyonal framework several dictyonalia in thickness at the growing edge; primary dictyonal frame consists at least in part of four-sided (square or rectangular) meshes; rays of dictyonalia extend only one-mesh in length to the next adjacent dictyonal centrum; longitudinally oriented dictyonal rays aligned and fused side-by-side to form longitudinal strands; schizorhysial channelisation developed by growth of framework in narrow vertical (dermal to atrial) and longitudinal oriented septa bridged by small patches of dictyonalia; such growth leaves a confluent system of small gauge channels 1–2 mm wide running mainly longitudinally, but connected transversely. Superficial cortices usually not developed but hypersilicification of dermal surfaces with swollen surface nodes occur in three genera; attachment of small hexactins to frameworks is rare; spiculation includes strongyloscopules; uncinates of intermediate size with poorly developed brackets and barbs are typical.

*Scope*: As of May 2016, Tretodictyidae was comprised of eight genera [5]. Here, we remove *Cyrtaulon* Schulze, 1886 from this family on the basis of molecular evidence (e.g., Fig. 7), and provisionally re-classify it as Sceptrulophora *inc. sed.* Inclusion of *Sclerothamnus* Marshall, 1875, *Sclerothamnopsis* Wilson, 1904, and *Tretocalyx* Schulze, 1901 is provisional and awaits to be tested with molecular data (see main text and Additional file 14).

*Remarks*: In the emended diagnosis, we removed “in all but one genus” after “strongyloscopules”. The lack of strongyloscopules in *Cyrtaulon* and the presence instead of the discohexaster-like “*Cyrtaulon*-spicule” [103] as the only scopule form, as well as presence of large uncinates with fully developed brackets and barbs (see Additional file 14), provide some morphological support for exclusion of *Cyrtaulon* from Tretodictyidae.

LYSSACINOSIDA ZITTEL, 1877

*Diagnosis (emended from Definition and Diagnosis of Reiswig* [88]*)*: Hexasterophora in which choanosomal megascleres remain as separate skeletal components, or, where fusion occurs it is by deposition of silica at contact points or as synapticula between diactine, tauactine, stauractine, or hexactine megascleres, or by tip-to-ray fusion of hexactine choanosomalia forming longitudinal strands of single continually extended rays with uniaxial connecting beams. Body form is typically a single ovoid, cup or tube bearing a single terminal osculum and deep atrial cavity, but might also be as branching fan or tubes, or tongue-like plate. Attachment to the substrate is either direct or by short peduncle or long stalk and is usually basiphytous with a thin basidictyonal plate of fused hexactins; lophophytous or rhizophytous attachment also occurs. Thin-walled forms may have a sieve plate over terminal osculum and a regular series of small parietal oscula; thicker-wall forms may occasionally bifurcate or grow one or more lateral diverticula, each with terminal osculum. Branching in stalks of cup-shaped members is poorly documented as a growth form and may result from secondary settlement. Choanosomal megascleres may be mainly diactins, or unfused or fused hexactins, or a combination of stauractins, tauactins, diactins, rarely pentactins. Dermalia may be large pentactins or hexactins unsupported by hypodermalia, or small hexactins, pentactins, stauractins or diactins supported by large pentactine hypodermalia. Atrialia may be either hexactins and/or pentactins and/or stauractins; hypoatrial pentactins may be present. Sceptrules and uncinates absent. Lateral prostalia may be absent or special diactins or extended hypodermal pentactins or simply the extended distal rays of choanosomal hexactins or pentactins; basalia of lophophytous forms may be monactine, diactine or pentactine anchors. Microscleres include single types or combinations of stellate and spherical discohexasters of regular or hemi-form, discoctasters, discohexactins, floricomes, discoplumicomes, strobiloplumicomes, sigmatocomes, oxyhexasters of regular and hemi-form, graphiocomes, trichasters, oxyhexactins, onychoexasters, and onychohexactins.

*Scope*: Four families are included in Lyssacinosida: Euplectellidae Gray, 1867, Leucopsacidae Ijima, 1903, Rossellidae Schulze, 1885, and Aulocalycidae Ijima, 1927. Two monospecific genera with uncertain family assignment (Lyssacinosida *inc. sed.*) are also included: *Clathrochone* Tabachnick, 2002 and *Hyaloplacoida* Tabachnick, 1989.

*Remarks*: Aulocalycidae is here included in Lyssacinosida on the basis of molecular evidence (e.g., Fig. 7), which is consistent with earlier taxonomic hypotheses (reviewed in [87]) and finds some morphological support from the widespread occurrence of synapticular fusion in this taxon (see main text for discussion). This result is based on sequence data from a single species, *Euryplegma auriculare* Schulze, 1886; the phylogenetic position of the remaining six genera currently included in Aulocalycidae (see Appendix 1) awaits to be determined with molecular data. The emended diagnosis reflects inclusion of additional characters present in Aulocalycidae. Inclusion of Aulocalycidae in Lyssacinosida requires abolishment of the order Aulocalycoida Tabachnick & Reiswig, 2000. Regarding the *inc. sed.* genera, *Clathrochone* is clearly not a member of any of the other families ([15, 16]; this study); molecular data from *Hyaloplacoida* are required to test the hypothesis that these two genera are sister groups (e.g., Figs. 5 and 10), which would justify erection of a separate family for them [14].

ROSSELLIDAE SCHULZE, 1885

LANUGINELLINAE GRAY, 1872

*Diagnosis*: Basiphytous, rarely lophophytous, often pedunculate, Rossellidae; dermalia hexactins, pentactins, stauractins, or diactins supported by large hypodermal pentactins; choanosomal spicules diactins, often supplemented by significant amount of hexactins; atrialia pentactins or hexactins often supported by large hypoatrial pentactins; dermal and atrial hexactins and pentactins frequently pinular; prostalia, if present, pentactins or diactins; microscleres include strobiloplumicomes, which may be absent in some species, oxy-, onycho-, or disco-tipped forms (hexasters, hemihexasters, hexactins); microdiscohexasters absent.

*Remarks*: The revised diagnosis reflects inclusion of *Caulophacus* Schulze, 1886 and *Caulophacella* Lendenfeld, 1915 in the subfamily. These genera were recently transferred from Rossellinae Schulze, 1885 to Lanuginellinae [83] based on molecular evidence [16, 83]. This move is further supported by our phylogenetic analyses of morphological data (e.g., Fig. 6, Additional file 17: Figure S3).

HEXASTEROPHORA *incertae sedis*

*Remarks*: Our phylogenetic results suggest that Dactylocalycidae Gray, 1867 is more closely related to Lyssacinosida Zittel, 1877 than to Sceptrulophora Mehl, 1992; consequently, order Hexactinosida Schrammen, 1912 (= Sceptrulophora + Dactylocalycidae) is abolished because it is not a monophyletic group. Furthermore, the genera *Heterorete* Dendy, 1916 and *Myliusia* Gray, 1859 also appear to be more closely related to Lyssacinosida than to Sceptrulophora and are consequently removed from Euretidae Zittel, 1877 (see above). Because erecting a Linnean taxon for a clade comprising Lyssacinosida, Dactylocalycidae, *Heterorete*, and *Myliusia* is problematic (see discussion in main text), we here provisionally treat the latter three taxa as Hexasterophora *inc. sed.* until their phylogenetic relationships are more precisely resolved. The genera *Deanea* Bowerbank, 1875, *Diaretula* Schmidt, 1879, and *Hyalocaulus* Marshall & Meyer, 1877 (formerly Hexactinosida *inc. sed.*), which were not included in our phylogenetic analyses, are also re-classified as Hexasterophora *inc. sed.* because their poor documentation does not allow confident assignment to any of the hexasterophoran orders*.*

DACTYLOCALYCIDAE GRAY, 1867

*Diagnosis (emended from Definition and Diagnosis of Reiswig* [84]*)*: Dictyonal Hexactinellida with rigid walls composed of branching systems of tubules. Channelization as cavaedia between branching tubules; tubule walls not channelized. Primary framework (tubule walls) is not euretoid in construction; dictyonal polyradial nodes result from tip-to-node and tip-to-tip fusion of dictyonalia. Body form as funnel or cup. Surface spicules as rough club-tipped pentactins and hexactins; sceptrules and uncinates absent. Microscleres include combinations of discohexasters, tylohexasters, hemioxyhexasters to oxyhexactins, and onychohexasters.

*Scope*: The family currently includes two genera, *Dactylocalyx* Stutchbury, 1841 and *Iphiteon* Bowerbank, 1869.

*Remarks*: Although monophyly of Dactylocalycidae could not be confirmed with molecular data (e.g., Fig. 7), in the absence of strong evidence for the contrary (see main text for discussion) there are no grounds for abolishing this taxon. The emended diagnosis reflects removal of *Auloplax* Schulze, 1904 [44].

## Additional files

Additional file 1: Morphological character matrix of Amphidiscophora in Nexus format. (NEX 2.05 KB)
Additional file 2: Morphological character matrix of Hexasterophora in Nexus format. (NEX 24.1 KB)
Additional file 3: Morphological character matrix of Amphidiscophora with annotated character list. (DOCX 79.8 KB)
Additional file 4: Morphological character matrix of Hexasterophora with annotated character list. (DOCX 258 KB)
Additional file 5: Complete 16S rDNA alignment in Nexus format. (NEX 58.8 KB)
Additional file 6: Complete COI alignment in Nexus format. (NEX 80.8 KB)
Additional file 7: Complete 28S rDNA alignment (incl. secondary structure) in Nexus format. (NEX 116 KB)
Additional file 8: Complete 18S rDNA alignment (incl. secondary structure) in Nexus format. (NEX 116 KB)
Additional file 9: Cleaned concatenated alignment (molecular supermatrix) in Nexus format. (NEX 354 KB)
Additional file 10: Secondary structure for molecular supermatrix (RAxML format). (TXT 4.69 KB)
Additional file 11: Partition information (gene boundaries) for molecular supermatrix (RAxML format). (TXT 80 bytes)
Additional file 12: Combined morphological character matrix of Amphidiscophora and Hexasterophora. (NEX 27.8 KB)
Additional file 13: Combined morphological character matrix of Amphidiscophora and Hexasterophora with additional characters not used for phylogenetic inference, including merged total-evidence MP tree of Amphidiscophora and Hexasterophora. (NEX 33.2 KB)
Additional file 14: Detailed account of the morphology-based and total-evidence phylogenies. (DOCX 172 KB)
Additional file 15: Figure S1. Phylogeny of Amphidiscophora inferred from the morphological data matrix with MrBayes. 50% majority rule consensus tree from 9000 post-burnin samples. Average standard deviation of split frequencies between two independent runs was 0.003873. Bayesian posterior probabilities <1.00 shown on branches. H, Hyalonematidae; M, Monorhaphididae; P, Pheronematidae. Scale bar, expected number of character replacements per character. (JPG 222 KB)
Additional file 16: Figure S2. Phylogeny of Amphidiscophora inferred from the morphological data matrix with RAxML. Bootstrap values >50% shown on branches (based on 1000 pseudoreplicates). H, Hyalonematidae; M, Monorhaphididae; P, Pheronematidae. Scale bar, expected number of character replacements per character. (JPG 179 KB)
Additional file 17: Figure S3. Phylogeny of Hexasterophora inferred from the morphological data matrix with MrBayes. 50% majority rule consensus tree from 9000 post-burnin samples. Average standard deviation of split frequencies between two independent runs was 0.026249. Bayesian posterior probabilities <1.00 shown on branches. Current family assignment given after genus names. Subfamilies indicated with letters: A = Acanthasci-nae (Rossellidae)/Aulocalycinae (Aulocalycidae), B = Bolosominae (Euplectelli-dae), C = Corbitellinae (Euplectellidae)/Chonelasmatinae (Euretidae)/Cyathellinae (Aulocalycidae), E = Euplectellinae (Euplectellidae)/Euretinae (Euretidae), L = Lanuginellinae (Rossellidae), R = Rossellinae (Rossellidae). Scale bar, expected number of character replacements per character. (JPG 714 KB)
Additional file 18: Figure S4. Phylogeny of Hexasterophora inferred from the morphological data matrix with RAxML. Bootstrap values >50% shown on branches (based on 650 pseudoreplicates). Current family assignment given after genus names. Subfamilies indicated with letters: A = Acanthascinae (Rossellidae)/Aulocalycinae (Aulocalycidae), B = Bolosominae (Euplectellidae), C = Corbitellinae (Euplectellidae)/Chonelasmatinae (Euretidae)/Cyathellinae (Aulocalycidae), E = Euplectellinae (Euplectellidae)/Euretinae (Euretidae), L = Lanuginellinae (Rossellidae), R = Rossellinae (Rossellidae). Scale bar, expected number of character replacements per character. (JPG 645 KB)
Additional file 19: Figure S5. Mitochondrial 16S rDNA phylogeny of Hexactinellida inferred with RAxML. Bootstrap values >50% shown on branches (based on 450 pseudoreplicates). Newly sampled species highlighted in bold. *, sequence data from mitochondrial genome sequencing projects [72, 73]. Scale bar, expected number of substitutions per site. (JPG 523 KB)
Additional file 20: Figure S6. Mitochondrial COI phylogeny of Hexactinellida inferred with RAxML from nucleotide alignment. Bootstrap values >50% shown on branches (based on 450 pseudoreplicates). Newly sampled species highlighted in bold. *, sequence data from mitochondrial genome sequencing projects [72, 73]. Scale bar, expected number of substitutions per site. (JPG 543 KB)
Additional file 21: Figure S7. 28S rDNA phylogeny of Hexactinellida inferred with RAxML. Bootstrap values >50% shown on branches (based on 600 pseudoreplicates). Newly sampled species highlighted in bold. Scale bar, expected number of substitutions per site. (JPG 514 KB)
Additional file 22: Figure S8. 18S rDNA phylogeny of Hexactinellida inferred with RAxML. Bootstrap values >50% shown on branches (based on 500 pseudoreplicates). Newly sampled species highlighted in bold. Scale bar, expected number of substitutions per site. (JPG 368 KB)
Additional file 23: Figure S9. Phylogeny of Hexactinellida inferred from concatenated molecular markers with MrBayes. 50% majority rule consensus tree from 25,000 post-burnin samples. Average standard deviation of split frequencies between two independent runs was 0.003858. Bayesian posterior probabilities <1.00 shown on branches. Newly sampled species highlighted in bold. *, 16S and COI sequence data from mitochondrial genome sequencing projects [72, 73]. Scale bar, expected number of substitutions per site. (JPG 615 KB)
Additional file 24: Figure S10. Phylogeny of Hexasterophora inferred with RAxML from reduced taxon set (one species per genus) of concatenated molecular markers. Bootstrap values >50% shown on branches (based on 450 pseudoreplicates). Newly sampled genera highlighted in bold. *, 16S and COI sequence data from mitochondrial genome sequencing project [72]. Scale bar, expected number of substitutions per site. (JPG 843 KB)
Additional file 25: Figure S11. Phylogeny of Hexasterophora inferred with RAxML from concatenated molecular and morphological data, including only genera with sequence data. Bootstrap values >50% shown on branches (based on 450 pseudoreplicates). Newly sampled genera highlighted in bold. *, 16S and COI sequence data from mitochondrial genome sequencing project [72]. Scale bar, expected number of substitutions/character replacements per site/character. (JPG 378 KB)
Additional file 26: Figure S12. Phylogeny of Hexasterophora inferred with MrBayes from concatenated molecular and morphological data, including only genera with sequence data. Fifty percent majority rule consensus tree from 45,000 post-burnin samples. Average standard deviation of split frequencies between two independent runs was 0.002564. Bayesian posterior probabilities <1.00 shown on branches. Newly sampled genera highlighted in bold. *, 16S and COI sequence data from mitochondrial genome sequencing project [72]. Scale bar, expected number of substitutions/character replacements per site/character.(JPG 422 KB)
Additional file 27: Figure S14. Phylogeny of Amphidiscophora inferred with MrBayes from concatenated molecular and morphological data, including all genera. Genera with sequence data highlighted in bold and connected with thick branches. Fifty percent majority rule consensus tree from 45,000 post-burnin samples. Average standard deviation of split frequencies between two independent runs was 0.003744. Bayesian posterior probabilities <1.00 shown on branches. H, Hyalonematidae; M, Monorhaphididae; P, Pheronematidae. Scale bar, expected number of substitutions/character replacements per site/character. (JPG 152 KB)
Additional file 28: Figure S13. Phylogeny of Amphidiscophora inferred with RAxML from concatenated molecular and morphological data, including all genera. Genera with sequence data highlighted in bold and connected with thick branches. Bootstrap values >50% shown on branches (based on 1000 pseudoreplicates). H, Hyalonematidae; M, Monorhaphididae; P, Pheronematidae. Scale bar, expected number of substitutions/character replacements per site/character. (JPG 162 KB)
Additional file 29: Figure S15. Placement of Amphidiscophora genera without sequence data (taxon names preceded by “QUERY__”) on the molecular backbone phylogeny using weighted morphology-based phylogenetic binning [22] as implemented in RAxML. H, Hyalonematidae; M, Monorhaphididae; P, Pheronematidae. (JPG 330 KB)
Additional file 30: Figure S16. Phylogeny of Hexasterophora inferred with RAxML from concatenated molecular and morphological data, including all genera. Genera with sequence data highlighted in bold and connected with thick branches. Bootstrap values >50% shown on branches (based on 550 pseudoreplicates). Current family assignment given after genus names: Aphr = Aphrocallistidae, Auloca = Aulocalycidae, Aulocy = Aulocystidae, Aulop = Auloplacidae, Crat = Craticulariidae, Crib = Cribrospongiidae, Dact = Dactylocalycidae, Diap = Diapleuridae, Eupl = Euplectellidae, Eure = Euretidae, Farr = Farreidae, Fiel = Fieldingiidae, Leuc = Leucopsacidae, Lyss inc. sed. = Lyssacinosida incertae sedis, Ross = Rossellidae, Scep inc. sed. = Sceptrulophora incertae sedis, Tret = Tretodictyidae, Unci = Uncinateridae. Subfamilies indicated with letters: A = Acanthascinae (Rossellidae)/Aulocalycinae (Aulocalycidae), B = Bolosominae (Euplectellidae), C = Corbitellinae (Euplectellidae)/ Chonelasmatinae (Euretidae)/Cyathellinae (Aulocalycidae), E = Euplectellinae (Euplectellidae)/Euretinae (Euretidae), L = Lanuginellinae (Rossellidae), R = Rossellinae (Rossellidae). Scale bar, expected number of substitutions/character replacements per site/character. (JPG 648 KB)
Additional file 31: Figure S17. Phylogeny of Hexasterophora inferred with MrBayes from concatenated molecular and morphological data, including all genera. Genera with sequence data highlighted in bold and connected with thick branches. Fifty percent majority rule consensus tree from 45,000 post-burnin samples. Average standard deviation of split frequencies between two independent runs was 0.043509. Bayesian posterior probabilities <1.00 shown on branches. Current family assignment given after genus names: Aphr = Aphrocallistidae, Auloca = Aulocalycidae, Aulocy = Aulo-cystidae, Aulop = Auloplacidae, Crat = Craticulariidae, Crib = Cribrospongiidae, Dact = Dactylocalycidae, Diap = Diapleuridae, Eupl = Euplectellidae, Eure = Eur- etidae, Farr = Farreidae, Fiel = Fieldingiidae, Leuc = Leucopsacidae, Lyss inc. sed. = Lyssacinosida incertae sedis, Ross = Rossellidae, Scep inc. sed. = Sceptrulo- phora incertae sedis, Tret = Tretodictyidae, Unci = Uncinateridae. Subfamilies in- dicated with letters: A = Acanthascinae (Rossellidae)/Aulocalycinae (Aulocalycidae), B = Bolosominae (Euplectellidae), C = Corbitellinae (Euplectelli- dae)/Chonelasmatinae (Euretidae)/Cyathellinae (Aulocalycidae), E = Euplectelli- nae (Euplectellidae)/Euretinae (Euretidae), L = Lanuginellinae (Rossellidae), R = Rossellinae (Rossellidae). Scale bar, expected number of substitutions/character replacements per site/character. (JPG 637 KB)
Additional file 32: Figure S18. Placement of Hexasterophora genera without sequence data (taxon names preceded by “QUERY__”) on the molecular backbone phylogeny (Additional file 24: Figure S10) using weighted morphology-based phylogenetic binning [22] as implemented in RAxML. Current family assignment given after genus names. Subfamilies indicated with letters: A = Acanthascinae (Rossellidae)/Aulocalycinae (Aulocalycidae), B = Bolosominae (Euplectellidae), C = Corbitellinae (Euplectellidae)/Chonelasmati- nae (Euretidae)/Cyathellinae (Aulocalycidae), E = Euplectellinae (Euplectellidae)/ Euretinae (Euretidae), L = Lanuginellinae (Rossellidae), R = Rossellinae (Rosselli- dae). (JPG 5.21 MB)

## Abbreviations

aMk2: Asymmetrical Mk 2-parameter model
ASR: Ancestral state reconstruction
bp: Base pairs
BS: Bootstrap support
CI: Consistency index
COI: Cytochrome oxidase subunit I
G4: Four-rate category gamma correction for among-site rate variation
GTR: General-time-reversible
inc. sed.: Incertae sedis
LCA: Last common ancestor
LD clade: Clade containing Lyssacinosida and Dactylocalycidae
MCMC: Markov Chain Monte Carlo
Mk: Markov-k model
Mk1: 1-parameter Mk model
ML: Maximum likelihood
MP: Maximum parsimony
MPT: Most parsimonious tree
MRC: Majority-rule consensus tree
mt: Mitochondrial
PCR: Polymerase chain reaction
pl: Proportional likelihood
PP: Posterior probability
RC: Rescaled consistency index
rDNA: Ribosomal DNA
RI: Retention index
s. nov.: Sensu novo
s.l.: Sensu lato
s.s.: Sensu stricto
TL: Treelength
VFB clade: Venus-flower-basket clade
