# Supplementary material for: An integrative systematic framework helps to reconstruct skeletal evolution of glass sponges (Porifera, Hexactinellida)
Source: Front Zool. 2017 Mar 21;14:18. doi: 10.1186/s12983-017-0191-3 (PMC5359874; doi:10.1186/s12983-017-0191-3)
Supplement: Additional file 3: — Morphological character matrix of Amphidiscophora with annotated character list. (DOCX 79.8 KB) [file 12983_2017_191_MOESM3_ESM.docx]

| **Character #** | **1** | **2** | **3** | **4** | **5** | **6** | **7** | **8** | **9** | **10** | **11** | **12** | **13** | **14** | **15** | **16** | **17** | **18** | **19** | **20** | **21** | **22** | **23** | **24** | **25** | **26** | **27** | **28** | **29** |
| --- | --- | --- | --- | --- | --- | --- | --- | --- | --- | --- | --- | --- | --- | --- | --- | --- | --- | --- | --- | --- | --- | --- | --- | --- | --- | --- | --- | --- | --- |
| *Hyalonema* (H) | 1 | 1 | 1 | 1 | 0 | 0 | 0 | 1 | 1 | 1 | 1 | 1 | 1 | 1 | 1 | 0 | 1 | 1 | 1 | 0 | 1 | 1 | 1 | 0 | 1 | 0 | 1 | 1 | 1 |
| *Chalaronema* (H) | 1 | 0 | 1 | 0 | 0 | 0 | 0 | 1 | 0 | 1 | 0 | 0 | 1 | 0 | 1 | 0 | 1 | 0 | 1 | 0 | 0 | 1 | 1 | 0 | 1 | 0 | 1 | 0 | 1 |
| *Compsocalyx* (H) | ? | ? | 1 | 0 | 0 | 0 | 0 | 1 | 0 | ? | ? | ? | 1 | 0 | 1 | 0 | 1 | 1 | 1 | 0 | 0 | 1 | 0 | 0 | 1 | 0 | ? | ? | 1 |
| *Lophophysema* (H) | 1 | 1 | 1 | 1 | 0 | 0 | 0 | 1 | 1 | 1 | 1 | 1 | 1 | 0 | 0 | 0 | 1 | 1 | 1 | 0 | 1 | 1 | 0 | 0 | 1 | 0 | ? | 1 | 1 |
| *Tabachnickia* (H) | 0 | 0 | 1 | 1 | 0 | 0 | 0 | 1 | 0 | 0 | 0 | 0 | 0 | 1 | ? | 0 | 1 | 1 | 1 | 0 | 0 | 0 | 0 | 0 | 1 | 0 | 0 | 0 | 1 |
| *Monorhaphis* (M) | 1 | 1 | 1 | 0 | 0 | 0 | 1 | 1 | 0 | 0 | 0 | 0 | 0 | 1 | 0 | 0 | 1 | 1 | 1 | 1 | 1 | 0 | 0 | 1 | 0 | 0 | 0 | 0 | 1 |
| *Pheronema* (P) | 1 | 0 | 1 | 1 | 1 | 1 | 0 | 0 | 1 | 0 | 0 | 0 | 1 | 1 | 0 | 1 | 1 | 1 | 1 | 1 | 0 | 0 | 0 | 1 | 1 | 1 | 0 | 0 | 0 |
| *Platylistrum* (P) | 1 | 1 | 1 | 0 | 1 | 1 | 1 | 0 | 1 | 0 | 0 | 0 | 0 | 1 | 0 | 1 | 0 | 1 | 0 | 0 | 0 | 0 | 0 | 1 | 1 | 1 | 0 | 0 | 0 |
| *Poliopogon* (P) | 1 | 1 | 0 | 0 | 1 | 1 | 1 | 0 | 1 | 0 | 0 | 0 | 0 | 1 | 0 | 1 | 1 | 1 | 1 | 0 | 1 | 0 | 0 | 1 | 1 | 1 | 0 | 0 | 0 |
| *Schulzeviella* (P) | 0 | 0 | 0 | 0 | 1 | 0 | 0 | 0 | 1 | 0 | 0 | 0 | 0 | 1 | 0 | 1 | 1 | 0 | 1 | 0 | 0 | 0 | 0 | 1 | 1 | 1 | 0 | 0 | 0 |
| *Semperella* (P) | 1 | 0 | 0 | 1 | 1 | 1 | 0 | 0 | 1 | 0 | 0 | 0 | 0 | 1 | 0 | 1 | 1 | 1 | 1 | 0 | 1 | 0 | 0 | 1 | 1 | 1 | 0 | 0 | 0 |
| *Sericolophus* (P) | 0 | 1 | 0 | 1 | 1 | 0 | 1 | 0 | 1 | 0 | 0 | 0 | 0 | 1 | 0 | 1 | 0 | 1 | 1 | 0 | 0 | 0 | 0 | 1 | 1 | 1 | 1 | 0 | 1 |

H, Hyalonematidae; M, Monorhaphididae; P, Pheronematidae

Annotated character list for Amphidiscophora

Numbers refer to "Character #" in the matrix given above. 0 = absent, 1 = present, ? = unknown. For further definitions and explanations, see Tabachnick and Reiswig (2002) and Leys et al. (2007).

Chars. 1-3: General presence of major spicule symmetries (number of rays). Six-rayed forms (hexactins) and five-rayed forms (pentactins) occur in all genera and are thus uninformative.

1. *Stauractins*.—Four-rayed spicules.

2. *Tauactins*.—Three-rayed spicules.

3. *Diactins*.—Two-rayed spicules.

Chars. 4-16: Megascleres. Major supporting skeletal elements, usually much larger than microscleres. General feature also present in Hexasterophora, except chars. 10-12, 15, 16, which are restricted to Amphidiscophora. Important are the number of rays (1-6) and the location of different types in the sponge body – dermal (outer surface layer), hypodermal (just below outer surface layer, sometimes protruding above surface), choanosomal (middle layer), hypoatrial (just below inner surface layer), and atrial (inner surface layer). Note that in Amphidiscophora, (hypo)dermal and atrial megasclere composition is rather uniform across genera (composed of five-rayed forms) and therefore not informative. [Chars. 24-29 are also megascleres but coded in a separate group because they are related to attachment of the sponges to the surface.]

4. *Choanosomal hexactins*.—Six-rayed megascleres in choanosomal tissue.

5. *Choanosomal pentactins*.—Five-rayed megascleres in choanosomal tissue.

6. *Choanosomal stauractins*.—Four-rayed megascleres in choanosomal tissue.

7. *Choanosomal tauactins*.—Three-rayed megascleres in choanosomal tissue.

8. *Choanosomal diactins*.—Two-rayed megascleres in choanosomal tissue.

9. *Hypoatrial pentactins*.—Large five-rayed megascleres supporting smaller dermal megascleres.

Chars. 10-12: Acanthophores. Special spicules located at the point where basalia (anchor spicules) leave the sponge body. Restricted to Hyalonematidae.

10. *Stauractine acanthophores*.—Four-rayed.

11. *Tauactine acanthophores*.—Three-rayed.

12. *Diactine acanthophores*.—Two-rayed.

13. *Diactine prostalia*.—Two-rayed megascleres protruding from dermal surface.

14. *Uncinates*.—Possibly one-rayed (monactine) megascleres ornamented with spines directed in one direction.

15. *Ambuncinates*.—Two-rayed megascleres similar to uncinates but with spines directed towards spicule center; restricted to Hyalonematidae.

16. *Sceptres*.—Special type of monactin prostal megascleres; restricted to Pheronematidae.

Chars. 17-21: Microscleres. Accessory skeletal elements usually much smaller than megascleres. General feature also present in Hexasterophora, although only six-rayed forms without secondary rays and pointed primary ray tips (oxyhexactins) occur in both subclasses. Besides oxyhexactins (present in all genera and hence excluded here), amphidiscophorans possess the eponymous amphidiscs, which are (usually) two-rayed spicules with an umbrella-like structure (umbel) at the end of each ray. In contrast to hexasters (the eponymous microscleres of Hexasterophora), the morphological diversity of amphidiscs is rather restricted, the major criterion being assignment to one of three size classes (macro, meso, micro). Other characters such as the ratio of umbel width to length are also taxonomically important, but their definition is rather vague, casting doubt on their phylogenetic value.

17. *Macramphidiscs*.—Amphidiscs assigned to large size class (>200 µm).

18. *Mesamphidiscs*.—Amphidiscs assigned to medium size class (~100-150 µm).

19. *Micramphidiscs*.—Amphidiscs assigned to small size class (~20-30 µm).

20. *Amphidiscs with fused umbels*.—Amphidiscs where the two umbels grew so long that they eventually touched in the middle and fused with each other.

21. *Amphidiscs with additional rays*.—Amphidiscs with up to four extra rays, then being hexactins with umbels (better called hexadiscs); occurence of these spicules is relatively rare.

22. *Apical cone*.—Conical structure in the body center formed by upper end of tuft of basalia; restricted to Hyalonematidae.

23. *Sieve plates*.—Spicular structures resembling perforated plates covering the main osculum; among Amphidiscophora restricted to Hyalonematidae.

Chars. 24-29: Basalia. Megascleres protruding from the bottom of the sponge body for attachment to the sediment. Especially important is the morphology of these spicules' distal ends.

24. *Oxyoidal or clavate diactin/monactin basalia*.—Two- or one-rayed basalia with pointed or club-shaped distal ends.

25. *Anchorate basalia*.—Basalia with distal ends featuring claw-like spines (teeth) for anchoring. 26. *Anchorate basalia with 1-2, mostly 4 teeth*.—Basalia with distal ends bearing 2, rarely 3-4 teeth; restricted to Pheronematidae.

27. *Anchorate basalia with multiple teeth*.—Basalia with distal ends bearing ≥ 4 teeth arranged in a whorl.

28. *Twisted anchorate basalia*.—Special case of char. 29 with twisted basalia; restricted to Hyalonematidae.

29. *Peduncle of naked basalia*.—Numerous long basalia forming a tuft devoid of soft tissue that elevates the sponge body above the surface.

References

Leys, S.P., Mackie, G.O., Reiswig, H.M. 2007. The biology of glass sponges. Adv. Mar. Biol. 52:1–145.

Tabachnick, K.R., Reiswig, H.M. 2002. Dictionary of Hexactinellida. In: Hooper, J.N.A., van Soest, R.W.M., editors. Systema Porifera. A Guide to the Classification of Sponges. New York: Plenum. p. 1224–1229.
