# Supplementary material for: An integrative systematic framework helps to reconstruct skeletal evolution of glass sponges (Porifera, Hexactinellida)
Source: Front Zool. 2017 Mar 21;14:18. doi: 10.1186/s12983-017-0191-3 (PMC5359874; doi:10.1186/s12983-017-0191-3)
Supplement: Additional file 14: — Detailed account of the morphology-based and total-evidence phylogenies. (DOCX 172 KB) [file 12983_2017_191_MOESM14_ESM.docx]

**Supplementary Discussion: Detailed account of the morphology-based and total-evidence phylogenies**

**Morphology-based phylogenies (Figures 4-6, Figures S1-S4)**

*Amphidiscophora (Figure 4, Figures S1-S2)*

Congruent with previous results [1], Pheronematidae is resolved as monophyletic (PP = 0.96 in the Bayesian tree). Characters in support of this clade, i.e., potential autapomorphies, include pentactine choanosomalia, sceptres, and anchorate basalia with 1-2 (at most 4) teeth, which is all in good agreement with the family diagnosis [2]. Other potential autapomorphies of the family are hypoatrial pentactins, although these also occur (likely as a convergence) in *Hyalonema* and *Lophophysema* (Hyalonematidae), and oxyoidal or clavate monactin/diactin basalia. The latter character also occurs in *Monorhaphis* (the sole genus of Monorhaphididae) in form of a single giant anchor spicule [3], which was interpreted as synapomorphic in [4] but might have to be interpreted as a convergence in light of our results (see below). In the MP and ML trees, the position of *Monorhaphis* is resolved as being inside Hyalonematidae, rendering this family paraphyletic. However, this result has to be viewed with caution (see below). In the following we focus on the MP and Bayesian trees, because the ML method tends to produce dubious results as was evident from the Hexasterophora analyses (see below).

*Pheronematidae*.—In the Bayesian tree, relationships within Pheronematidae are completely unresolved. In the MP tree, *Sericolophus*, *Semperella*, *Poliopogon*, *Platylistrum*, and *Pheronema* are recovered as a clade to the exclusion of *Schulzeviella*, although character support for this grouping remains largely elusive. However, a sister-group relationship of *Schulzeviella* to the remainder of the family is congruent with molecular results (this study). The presence of stauractins among choanosomalia supports a clade of *Semperella*, *Poliopogon*, *Platylistrum*, and *Pheronema* to the exclusion of *Sericolophus*. Within this clade, *Semperella* and *Poliopogon*, and *Platylistrum* and *Pheronema* are sister groups, respectively. Character support for these two clades is rather weak: *Semperella* and *Poliopogon* share the presence of diactins, which are otherwise restricted to Hyalonematidae and *Monorhaphis*, and *Platylistrum* and *Pheronema* share the presence of amphidiscs with additional rays, which also occur in Hyalonematidae (*Hyalonema*, *Lophophysema*) and *Monorhaphis*. The position of *Pheronema* conflicts with molecular results that suggest *Pheronema* is more closely related to *Sericolophus* than to *Semperella* [1].

*Hyalonematidae and Monorhaphididae*.—Four of the five genera of Hyalonematidae ("core hyalonematids" hereafter) form a monophyletic group (PP = 0.98). In terms of characters, this clade is supported by the presence of an apical cone, ambuncinates (although secondarily lost in *Lophophysema*), and stauractine acanthophores (although somewhat provisional since the acanthophores of *Compsocalyx* have not been found yet). Another possible autapomorphy are anchorate basalia with multiple teeth. However, the basalia of *Compsocalyx* and *Lophophysema* are insufficiently known due to poor specimen preservation [5], and multi-toothed basalia are also found in *Sericolophus* (Pheronematidae). Finally, diactine prostalia could be synapomorphic for the core hyalonematids, having only convergently evolved once in Pheronematidae (*Pheronema*).

Within the core hyalonematids, *Hyalonema* and *Lophophysema* are sister groups (PP = 0.99). These genera share the presence of tauactins and diactins among acanthophores, as well as twisted tufts of anchorate basalia, but these characters might also be present in *Compsocalyx* (see above). Another character unique to these two genera among Hyalonematidae is hypoatrial pentactins, which are however also characteristic for Pheronematidae (see above). In the MP tree, *Compsocalyx* and *Chalaronema* are successive sister groups to *Hyalonema* + *Lophophysema*, but there seem to be no clear-cut characters supporting this grouping.

In the MP tree, *Tabachnickia* (formerly known as *Platella*; cf. [6]), a monospecific hyalonematid genus lacking some of the typical features of this family [5], and *Monorhaphis* (Monorhaphididae) form successive sister groups to the core hyalonematids, rendering Hyalonematidae paraphyletic or suggesting inclusion of *Monorhaphis* in that family. This nested position of *Monorhaphis* within Hyalonematidae only seems to gain support from the shared presence of stauractins in that genus and the core hyalonematids to the exclusion of *Tabachnickia*. However, stauractins also occur within Pheronematidae (see above), and we do not consider this sufficient evidence for paraphyly of Hyalonematidae. Furthermore, *Tabachnickia* also might possess ambuncinates (here coded as missing data; cf. [5]), which, if confirmed, would definitely support monophyly of the family. A possible synapomorphy of *Monorhaphis* and Hyalonematidae is the presence of diactins among choanosomalia, a feature that is entirely absent from Pheronematidae. However, diactins are relatively rare in the choanosomal skeleton of *Monorhaphis*, which is mainly composed of tauactins, whereas in Hyalonematidae diactins dominate, which casts some doubt on the homology of this character. A "naked" peduncle – in case of *Monorhaphis* formed by a single giant spicule, in Hyalonematidae by a tuft of spicules ­– could also be synapomorphic, but *Sericolophus* (Pheronematidae) also forms a peduncle from a tuft of naked basalia. More importantly, the anchor spicule of *Monorhaphis* does not possess teeth at the distal end, as is typical for the basalia of Hyalonematidae, and might thus be homologous to oxyoidal basalia of Pheronematidae instead [4].

*Hexasterophora (Figures 5-6; Figures S3-S4)*

The ML tree inferred from the Hexasterophora matrix (Figure S4) displays a highly dubious topology where rossellids form a paraphyletic grade leading to the remaining taxa, and Leucopsacidae are nested within Euplectellidae (although bootstrap values are extremely low for most nodes, resampling methods are probably not very useful in our case; see main text). Given its strong conflict with molecular evidence and morphological taxonomy, we do not discuss this topology further. The Bayesian tree (Figure S3) is largely congruent with, but less well resolved than, the MP tree (Figures 5-6). Therefore, we focus our discussion on the latter and discuss differences to the Bayesian tree where appropriate.

Of the four orders of Hexasterophora, only Aulocalycoida (except for *Tretopleura* in the Bayesian tree^[[1]](#footnote--1)^) and the small relict group Lychniscosida are recovered as monophyletic. Monophyly of Lychniscosida has never been in doubt, as it is unambiguously supported by a choanosomal skeleton composed of fused lychniscs (lantern-like hexactine megascleres). The three extant lychniscosidan genera further share the presence of pentactine prostalia, which are otherwise restricted to Lyssacinosida. The sceptrule- and uncinate-lacking euretid genus *Heterorete* is reconstructed as the sister group of Lychniscosida in the MP tree. Possible synapomorphies are fused surface networks, which however also occur in *Laocoetis* and *Fieldingia*, and onychohexasters, which are also found in numerous genera of Sceptrulophora and Lyssacinosida, as well as *Dactylocalyx* (Dactylocalycidae), likely as convergences. In the Bayesian tree, the sister group of Lychniscosida is not resolved, and *Heterorete* groups with *Myliusia* instead.

The main autapomorphy of Aulocalycoida is longitudinal strands formed by continuous extension of dictyonalia. This taxon's position is unresolved in the Bayesian tree, but in the MP tree it is reconstructed as being deeply nested within Sceptrulophora. This position is intriguing because it implies that (par)aulocalycoid dictyonal frameworks evolved from a euretoid ground state, and that sceptrules and uncinates have been secondarily lost in Aulocalycidae (but see main text for contrasting molecular phylogenetic results). Furthermore, the sister group of Aulocalycoida is inferred to be Auloplacidae, a taxon also characterized by non-euretoid frameworks that have some similarities to the (par)aulocalycoid construction types: longitudinal strands with uniaxial connecting beams; axon-to-ray fusion of dictyonalia (only shared with Uncinateridae and *Cyathella* and here interpreted as secondarily lost in Aulocalycinae); and tip-to-ray fusion of dictyonalia (also present in Aphrocallistidae and *Pleurochorium*; see below).

Lychniscosida + *Heterorete* and the Sceptrulophora *sensu lato* clade form a clade with the second sceptrule- and uncinate-lacking euretid genus, *Myliusia*, the exact placement of which is not resolved (but see Figure S3). Dictyonal skeletons with parallel ray fusion are shared between these groups, although the topology implies secondary loss of this character in Auloplacidae + Aulocalycoida (followed by a "reversal" in *Cyathella*). The fact that the problematic genera *Myliusia* and *Heterorete*, which are currently classified in Sceptrulophora: Euretidae, although they lack sceptrules and uncinates [9], group outside Sceptrulophora *s.l.* implies that these spicule types are primitively absent in these genera.

In agreement with molecular results ([1, 10, 11]; this study), Dactylocalycidae comes out closer to Lyssacinosida than to other dictyonal groups, although characters supporting this clade are difficult to pin down. First of all, most of the dictyonal framework characters present in other dictyonal taxa are absent from Dactylocalycidae, explaining why this family does not group with these taxa, and hinting at the possibility that dactylocalycid dictyonal frameworks evolved convergently to similar structures in other groups. One possible synapomorphy of Dactylocalycidae and Lyssacinosida are (non-uncinate) diactins, which are otherwise only found in a few genera of Sceptrulophora (*Aphrocallistes*, *Fieldingia*, *Pinulasma*, *Pityrete*) and Aulocalycidae (*Aulocalyx*, and possibly *Cyathella*). Also, diactine prostalia are only found in *Aulocalyx* among the genera grouping outside of the Dactylocalycidae + Lyssacinosida clade, whereas they are quite widespread (at least in Rossellidae) among Lyssacinosida, and also occur in Dactylocalycidae (HMR and MD, pers. obs.). Finally, atrial hexactins are relatively rare in Sceptrulophora and Aulocalycoida, and absent from Lychniscosida, but present in Dactylocalycidae and the majority of lyssacinosidan genera. None of these features, however, is strong enough to provide a meaningful diagnosis for a higher taxon comprising Dactylocalycidae and Lyssacinosida.

In the Bayesian tree, relationships between Dactylocalycidae and the lyssacinosidan families are unresolved, so these results are at least consistent with monophyly of Lyssacinosida. In contrast, the MP tree displays Dactylocalycidae as the sister group to a Euplectellidae + Rossellidae clade to the exclusion of Leucopsacidae and Lyssacinosida *inc. sed.*, rendering Lyssacinosida paraphyletic. However, there are no clear-cut characters in support of this grouping and it also strongly conflicts with molecular results ([11]; this study). Lyssacinosida is morphologically well-delineated, and its members are easily recognizable by the presence of lyssacine (i.e., non-dictyonal) choanosomal skeletons in combination with hexasterous microscleres. However, amphidiscophorans are also lyssacine, so this character has to be interpreted as plesiomorphic [12], unless it can be shown that this type of skeletal architecture evolved convergently from a dictyonal ground state in Lyssacinosida (see discussion in [10]). Perhaps the best candidate for an autapomorphy of this order is a choanosomal skeleton composed mainly of diactins, which occurs in the majority of lyssacinosidans and is missing (by definition) from all dictyonal taxa. This interpretation would only require secondary losses in Leucopsacidae, *Hyaloplacoida*, and 10 genera of Euplectellidae (see below), and one convergent evolution in Hyalonematidae (Amphidiscophora). Other possible autapomorphies include stauractins, tauactins, sigmatocomes, and discasters, spicule types that are absent from or rare in dictyonal genera. However, their occurence is rather scattered across the genera of Lyssacinosida (sigmatocomes, discasters) or they are also widespread in Amphidiscophora (stauractins, tauactins), which complicates their phylogenetic interpretation.

In summary, these results support the previously suggested paraphyly of Hexactinosida (reviewed in [8]), confirm monophyly of Lychniscosida and Aulocalycoida, and suggest a revised scope of Sceptrulophora. In contrast, the phylogenetic status of Lyssacinosida cannot be unambiguously resolved by the present morphological dataset.

Of the 12 hexasterophoran families with more than one genus, nine are recovered as monophyletic groups (Aphrocallistidae, Aulocalycidae, Aulocystidae, Auloplacidae, Dactylocalycidae, Euplectellidae, Farreidae, Leucopsacidae, Rossellidae) and three are inferred to be para- or polyphyletic (Uncinateridae, Euretidae, Tretodictyidae). Character support for these various groupings, internal relationships of the larger families, as well as the inferred positions of the four monogeneric families (Craticulariidae, Cribrospongiidae, Diapleuridae, and Fieldingiidae) and the three *incertae sedis* genera (*Clathrochone*, *Hyaloplacoida*, *Sarostegia*) are discussed below.

*Aulocystidae and Diapleuridae (Lychniscosida)*.—*Lychnocystis* and *Neoaulocystis* form a clade to the exclusion of *Scleroplegma* (Diapleuridae), confirming monophyly of Aulocystidae. An unambiguous synapomorphy of these two genera is the arrangement of lychniscs in rectangular arrays or ranks. They further share a hypersilicified dermal cortex, which however also convergently evolved in Craticulariidae and within Tretodictyidae.

*Uncinateridae (Aulocalycoida)*.—Uncinateridae (*Uncinatera* + *Tretopleura*) is here reconstructed as a paraphyletic group, with *Uncinatera* more closely related to Aulocalycidae than to *Tretopleura* (but see Figure S3 where the family is polyphyletic). This might be due to the fact that of the character complex constituting the paraulocalycoid framework architecture – the defining feature of Uncinateridae [13] – only a single character (longitudinal strands with overlapping continuous rays) is unique to these two genera, whereas other aspects, such as axon-to-ray fusion of dictyonalia, are also present in Auloplacidae and/or Aulocalycidae (or even in Dactylocalycidae, in the case of tip-to-tip fusion of dictyonalia). On the other hand, synapomorphies of *Uncinatera* and Aulocalycidae remain elusive, except perhaps the loss of scopules, which is not a very strong character. Conversely though, besides their paraulocalycoid framework, *Uncinatera* and *Tretopleura* have not much in common either [13].

*Aulocalycidae (Aulocalycoida)*.—Aulocalycidae appears to be well-defined by dictyonal strands formed by single continuous rays, a character that is here resolved as an unambiguous autapomorphy. Loss of uncinates and scopules (but see main text for contrasting molecular and total-evidence results) appears as an additional synapomorphy of these six genera. Subfamily Aulocalycinae – all genera except *Cyathella* (Cyathellinae) – is also supported as a clade. Synapticular bridging between dictyonalia seems to be the most obvious potential autapomorphy of that taxon. Although this character is also found in *Heterorete*, *Tretochone*, and *Pleurochorium* (Euretidae), it does not play such a dominant role in the framework construction of these genera and probably evolved convergently. Another character resolved as an autapomorphy of the subfamily is the secondary absence of axon-to-ray fusion of dictyonalia (see above).

Resolution within Aulocalycinae differs between the Bayesian and MP trees. In the latter, only *Ijimadictyum* and *Rhabdodictyum* form a clade, which is supported by the presence of spirodiscohexasters (which also convergently evolved in three genera of Euplectellidae though), whereas relationships among the remaining genera are unresolved. In contrast, in the Bayesian tree, the positions of *Rhabdodictyum* and *Aulocalyx* are unresolved and the remaining genera form a clade. These four genera share the presence of skeletal channelization, which is absent in the other two. However, channelization is widespread among dictyonal taxa and we do not consider it informative for understanding relationships of these genera. In contrast, *Ijimadictyum* and *Rhabdodictyum* are virtually identical in loose spiculation [14], so we consider their sister-group relationship to be the better-supported hypothesis.

*Auloplacidae (Sceptrulophora)*.—Auloplacidae had only recently been resurrected for the genus *Auloplax* (formerly in Dactylocalycidae) after discovery of two new species, the definitive proof of autochtonous scopules, and a more detailed investigation of the dictyonal framework construction [15]. A second genus, *Dictyoplax*, with a very similar type of framework was recently recognized and also assigned to Auloplacidae [16]. Here, these two genera group together, confirming monophyly of the family. The defining synapomorphy of the two genera is a differentiation of the framework into distinct sieve- and ridge areas, which is not known from any other group. Other aspects of the "auloplacoid" framework construction, however, appear to be shared with Aulocalycoida, the sister group of Auloplacidae according to the MP analysis (see above).

*Euretidae (Sceptrulophora)*.—Monophyly of Euretidae, the largest and at the same time most poorly defined family of Sceptrulophora (see discussions in [8, 16]), is – as expected – not supported by our analysis, even if one assumes that the sceptrule- and uncinate-lacking *Myliusia* and *Heterorete* are simply misclassified in that taxon (see above). Although relationships among the sceptrule- and uncinate-bearing euretids are very well resolved in Figure 5, we doubt that this high resolution is meaningful in all of its aspects. For instance, although we could determine some potential autapomorphies associated with the deeper nodes within Sceptrulophora *s.l.*, these were always followed by multiple secondary losses and never involved really strong characters. Furthermore, large parts of this topology are incongruent with molecular evidence ([7, 16]; this study). Therefore, we only discuss the placement of some euretid genera in the following subsections, where relevant for the relationships of other families.

*Fieldingiidae (Sceptrulophora)*.—*Fieldingia*, the sole genus of Fieldingiidae, is here resolved as the sister group to all other Sceptrulophora *s.l.*. This appears to be mainly due to its complete lack of a regular pattern of the dictyonal framework, which clearly sets this genus aside from other sceptrulophorans. However, details of its framework construction are incompletely known such that we had to code some important characters as missing for this genus, which could have biased the analysis. A clade of all sceptrulophorans except *Fieldingia* is further supported by the presence of dermal sceptrules (secondarily lost within Aulocalycoida). However, the primitive absence of this character in *Fieldingia* is questionable as these sponges' dermal surfaces form massive crusts that preclude the presence of loose spicules, a condition that we consider derived. Thus, an early-branching position of *Fieldingia* – although not unlikely – has to be interpreted with caution until its skeletal anatomy is better understood.

Sarostegia *(Sceptrulophora* inc. sed.*)*.—This monospecific genus, which was originally classified in Euretidae [17], then Farreidae [18], and then back in Euretidae [7], was only recently re-classified as *incertae sedis* based on molecular phylogenetic results [16]. It is here resolved as the sister group to all the remaining Sceptrulophora *s.l.* except *Fieldingia*. The only character supporting this position is the lack of longitudinal strands with biaxial connecting beams, which is interpreted as a synapomorphy of all remaining genera (secondarily lost in Aulocalycoida and "re-gained" in *Cyathella*; also convergently evolved in *Heterorete*). However, an early-branching position of *Sarostegia* is clearly contradicted by molecular evidence, suggesting that secondary loss of this character is more likely (see main text).

*Craticulariidae and Cribrospongiidae (Sceptrulophora)*.—Our analysis recovered a sister-group relationship between *Laocoetis* and *Stereochlamis*. This result is quite plausible and expected, because Craticulariidae and Cribrospongiidae share a distinct skeletal channelization pattern in the form of diplorhyses. In contrast, character support for a close relationship of *Laocoetis* + *Stereochlamis* to *Tretocalyx* and *Pityrete*, as reconstructed here, remains elusive.

*Farreidae (Sceptrulophora)*.—Farreidae is recovered as a monophyletic taxon, consistent with previous molecular data and interpretation of morphology [7]. The clade gains support from the presence of clavules (but see File S4 for the case of *Asceptrulum*) and a primary dictyonal skeleton with biaxial connecting beams that is two-dimensional at growth margin – i.e., a farreoid framework. However, the latter character is also found in the euretids *Bathyxiphus* (cf. [19]: 211) and *Conorete* [15]. Indeed, farreoid frameworks are here interpreted as a synapomorphy of Farreidae and these two genera, grouping them together in a clade. The inclusion of the poorly known monospecific *Bathyxiphus* in Farreidae remains a realistic option because its entire framework is farreoid and the autochtonous vs. allochtonous nature of clavules and/or scopules reported from this species has not been clarified yet (cf. [19]: 211). In contrast, within *Conorete*, only *C. gordoni* has an entirely farreoid framework [15], and homology to the skeletons of Farreidae requires further study [16]. In any case, the here reconstructed position of Farreidae relative to other sceptrulophoran families is highly incongruent with molecular results ([16]; this study).

Inside Farreidae, *Farrea* and *Aspidoscopulia* are most closely related, and together are sister to *Claviscopulia*, whereas the positions of *Asceptrulum* and *Lonchiphora* remain undetermined. The *Farrea* + *Aspidoscopulia* clade is supported by the presence of anchorate clavules with a whorl of hooks below the head. Potential synapomorphies of this clade and *Claviscopulia* are long-principalled oxy- and discohexasters, although these spicules are also widespread in other sceptrulophoran families.

*Aphrocallistidae (Sceptrulophora)*.—Monophyly of Aphrocallistidae (*Aphrocallistes* + *Heterochone*) is recovered here, in line with molecular results (reviewed in [8]). The main and defining autapomorphy of the family are cylindrical diarhyses (honeycomb-shaped skeletal channels penetrating the whole body wall). Additional characters are tylohexasters (also present as convergent autapomorphies in a handful of unrelated genera) and tip-to-node fusion of dictyonalia (also found in *Cyathella*, *Psilocalyx*, and Dactylocalycidae, likely as convergences).

Our analysis recovered the family as sister to the Auloplacidae + Aulocalycoida clade (see above). This result seems to be caused by the presence of tip-to-ray fusion of dictyonalia in these three taxa plus *Pleurochorium* (Euretidae), which is the immediate outgroup to this assemblage, and the absence of longitudinal dictyonal strands with biaxial connecting beams in Aphrocallistidae and Auloplacidae + Aulocalycoida (the latter is interpreted as an apomorphic secondary loss, followed by a "reversal" in *Cyathella*). However, we consider this reconstruction unlikely. First, tip-to-ray fusion is not an integral part of the framework construction of Aphrocallistidae and *Pleurochorium*, and probably evolved convergently in these taxa. Second, absence of longitudinal strands cannot be considered positive evidence for the affinities of Aphrocallistidae. Finally, the position of the family reconstructed here contradicts molecular results that clearly support a close relationship of Aphrocallistidae to a clade of Farreidae + Euretidae *part.* to the exclusion of Auloplacidae and Uncinateridae ([16]; this study).

*Tretodictyidae (Sceptrulophora)*.—Only a subset of tretodictyids – *Tretodictyum*, *Hexactinella*, *Psilocalyx*, *Anomochone*, and *Cyrtaulon* – are recovered as a clade by our analysis ("core tretodictyids" hereafter; but see molecular phylogenetic results regarding the position of *Cyrtaulon*). The other three genera – *Sclerothamnus*, *Sclerothamnopsis*, and *Tretocalyx* – appear to be unrelated to the core tretodictyids (but see Figure S4), which somehow reflects the notion that their current classification in Tretodictyidae (at least concerning *Sclerothamnopsis* and *Tretocalyx*) is provisional [20].

Tretodictyidae is characterized by a number of features, none of which is unambiguously interpretable as a synapomorphy of all genera, however. First of all, the main diagnostic character of the family is a skeletal channelization in the form of schizorhyses. Schizorhyses occur in all core tretodictyids, and also in *Sclerothamnus* and *Sclerothamnopsis*, whereas the channelization of *Tretocalyx* is insufficiently known [20]. Thus, this character at least supports the inclusion of *Sclerothamnus* and *Sclerothamnopsis* in Tretodictyidae. Curiously, this was supported by the otherwise rather scrambled ML phylogeny (Figure S4), whereas the MP tree implies convergent evolution of schizorhyses in these two genera. We could not determine any clear-cut characters in support of their inferred position, and the same also applies to the position of *Tretocalyx* as sister to Cribrospongiidae + Craticulariidae. Thus, the placement of *Sclerothamnus*, *Sclerothamnopsis*, and *Tretocalyx* appears to be the result of more or less random resolution.

Another important character – interpreted by Mehl [12] as autapomorphic for Tretodictyidae ­– is the arrangement of uncinates in surface brushes (bundles). However, *Sclerothamnus* lacks this feature, and its presence or absence in *Anomochone*, *Cyrtaulon*, and *Sclerothamnopsis* appears to be unclear. Interestingly though, the euretids *Gymnorete* and *Endorete* also possess bundled uncinates, and indeed these genera appear to be successive sister groups to the core tretodictyids. *Gymnorete* further has uncinates with weakly developed brackets and barbs, another typical tretodictyid feature, which is here interpreted as synapomorphic with core tretodictyids. However, *Cyrtaulon* has proper uncinates, which complicates polarization of this character. Moreover, weakly developed uncinates are also found in *Sclerothamnopsis* and *Auloplax*. While in *Auloplax* these spicules probably evolved convergently, their presence in *Sclerothamnopsis* provides further support for its inclusion in Tretodictyidae.

A hypersilicified dermal cortex and swollen framework nodes have been reported from all core tretodictyid genera except the type genus, *Tretodictyum*. Indeed, *Tretodictyum* is resolved as the sister group to the remaining core tretodictyids, implying that these characters are synapomorphic for *Hexactinella*, *Psilocalyx*, *Anomochone*, and *Cyrtaulon*. However, this topology contradicts molecular results (and taxonomists' intuition) that strongly support a close relationship of *Tretodictyum* and *Hexactinella* to the exclusion of *Psilocalyx* [7]. Moreover, hypersilicified dermal cortices are also found in *Laocoetis* and Aulocystidae (see above), and swollen framework nodes also occur in *Iphiteon*, *Myliusia*, *Pararete*, and *Verrucocoeloidea*.

Finally, Mehl [12] also considered lophodiscohexasters as a synapomorphy of *Hexactinella*, *Tretodictyum*, and *Psilocalyx*, and these spicules are actually also present in *Sclerothamnus*. However, they are also found in six non-tretodictyid genera, namely *Farrea*, *Tretopleura*, *Neoaulocystis*, *Gymnorete*, *Endorete*, and *Periphragella*. Interestingly, this character is resolved as synapomorphic for the latter three genera and the core tretodictyids, with secondary loss in *Anomochone* and *Cyrtaulon*.

In summary, although not unambiguously supported, the monophyly of Tretodictyidae (possibly in a revised scope) seems likely. Although a deeply nested position of this family within Sceptrulophora, as inferred here, clearly contradicts molecular evidence ([7, 16]; this study), a closer relationship of certain euretid genera to Tretodictyidae cannot be ruled out as it finds some support from loose-spiculation characters.

*Dactylocalycidae ("Hexactinosida").—Iphiteon* and *Dactylocalyx* group together, confirming monophyly of Dactylocalycidae in its current scope. The main synapomorphy of these two genera is a special type of body wall construction represented by cavaedial channelization between branching tubules. Furthermore, tip-to-node and tip-to-tip contact are the only means of dictyonal fusion in Dactylocalycidae, resulting in a haphazard pattern. Although tip-to-node fusion also occurs in Aphrocallistidae, *Cyathella* (Aulocalycidae), and *Psilocalyx* (Tretodictyidae), and tip-to-tip fusion is also found in Uncinateridae, in these taxa these modes of contact never occur together and are always accompanied by additional means of dictyonal fusion. Thus, the "dactylocalycoid" framework construction appears to be unique and might represent a convergent solution to making a dictyonal body plan (see above).

*Lyssacinosida* incertae sedis*.—Hyaloplacoida* and *Clathrochone* are here reconstructed as sister taxa, supporting the idea of a fourth family of Lyssacinosida (discussed in [8]). However, the only strong support for this grouping comes from the presence of dermal pentactins with siliceous flattened structures, the homology of which is not entirely clear. Otherwise, these taxa have not much in common, and the position of *Hyaloplacoida* ultimately has to be resolved with molecular data [8].

*Leucopsacidae (Lyssacinosida)*.—Leucopsacidae is here reconstructed as monophyletic, with *Leucopsacus* and *Chaunoplectella* being more closely related to each other than either is to *Oopsacas*. Synapomorphies of these three genera are choanosomal skeletons dominated by unfused hexactins, and dermal/atrial megascleres that are poorly differentiated from choanosomalia, which basically reflects the family diagnosis [21]. *Leucopsacus* and *Chaunoplectella* share stauractins, sigmatocomes, synapticular fusion of megascleres, and tubular peduncles. However, all of these characters are also found outside Leucopsacidae. Nevertheless, these two genera are considered very closely related, differing only by the presence or absence of discohexactins, and there might even be grounds to synonymize them in future revisions [21].

*Euplectellidae (Lyssacinosida)*.—All euplectellid genera group together in a clade that is mainly supported by the presence of large dermalia with long thin rays. While this is not a very strong character, its inclusion was essential to recover monophyly of the family, because other characters considered typical for this taxon actually only occur in restricted subsets of its genera or are also found elsewhere.

First of all, the statements of Reiswig [22] and Tabachnick [23] that Euplectellidae is well-differentiated from the other families of Lyssacinosida by choanosomal skeletons composed of a mix of different megasclere types actually seems unwarranted. Indeed, this character only occurs in 10 of the 26 genera, whereas the remaining 16 genera have choanosomalia dominated by diactins, as is typical also for Rossellidae and *Clathrochone*. Likewise, hexactine dermalia are considered diagnostic for Euplectellidae among Lyssacinosida, but they are missing in four genera and also occur in 11 genera of Rossellidae. Another characteristic feature of Euplectellidae is synapticular fusion of megascleres, and it is indeed resolved as an autapomorphy here; however, it is missing in five genera and also occurs in Leucopsacidae and many genera of Rossellidae (primarily within the context of peduncle formation; see below). The iconic cylindrical "venus-flower basket" body shape with longitudinal and circular skeletal beams and regularly spaced lateral oscula only characterizes seven genera. Floricomes – the most distinct type of microsclere that is often cited as an autapomorphy of Euplectellidae [1, 11, 12] – are unknown from 10 genera, and hexasters with floricoidal secondary ray ends are also known from *Leucopsacus* (Leucopsacidae), *Rhabdocalyptus* and *Staurocalyptus* (Rossellidae), and *Myliusia* and *Heterorete* (Euretidae). Graphiocomes occur in 54% of euplectellid genera, but also in the lychniscosidan *Neoaulocystis*, which is here interpreted as a convergence (*contra* Mehl [12], who hypothesized a sister-group relationship of Lychniscosida and Euplectellidae based on this character). Sigmato- or drepanocomes are only present in 10 genera, and are also found in *Leucopsacus* and *Chaunoplectella* (Leucopsacidae), and one species of *Crateromorpha* (Rossellidae). Finally, discoplumicomes (see File S4) are only known from five genera of Euplectellidae. Thus, although the family is widely considered to be morphologically well supported [8], unambiguous synapomorphies of these 26 genera are actually hard to pin down. Nevertheless, molecular data have so far upheld the monophyly of Euplectellidae [8].

None of the three currently recognized subfamilies (Euplectellinae, Bolosominae, Corbitellinae) is recovered as a natural group here, which is in line with molecular evidence [11]. Euplectellinae is solely defined by a lophophytous mode of attachment to the substrate [23]. Although we excluded this character from the final matrix, we did include toothed anchorate basalia, which are present in six of the seven genera of this subfamily (the seventh genus, *Chaunangium*, has bundles of non-toothed basalia instead). Nonetheless, this character seems to conflict with many others: in the Bayesian tree, only *Acoelocalyx*, *Malacosaccus*, *Docosaccus*, and *Placopegma* form a clade, whereas the type genus of the (sub)family, *Euplectella*, forms an unrelated clade with *Holascus* (also Euplectellinae), implying that toothed basalia evolved twice convergently in Euplectellidae; the MP tree even implies four independent origins. The sister-group relationship of *Euplectella* and *Holascus* is also supported by choanosomalia containing pentactins; they further share stauractine and tauactine choanosomalia with *Dictyaulus*, which forms their sister group. The close relationship of *Acoelocalyx* and *Malacosaccus* is consistent with molecular evidence [1] and finds support from a peculiar peduncle morphology (see [1] and File S4); another synapomorphy according to the present analyses appears to be the loss of diactins.

Corbitellinae is the largest and most weakly defined subfamily (basiphytous without peduncle) and accordingly these genera are more or less scattered across the tree. In contrast, Bolosominae is morphologically quite distinct, with most of its genera having a mushroom- or wineglass-like body with a tubular peduncle and lateral oscula-bearing outgrowths on the main body. Although monophyly of this subfamily is contradicted by molecular studies with a limited taxon sampling ([10]; this study), it can at least be expected that some of these genera are closely related to each other, also because of similarities in spiculation. Indeed, in the MP tree all bolosomins with the typical body shape group together in a clade ("core bolosomins" hereafter) that only excludes *Hyalostylus*, *Caulocalyx*, and the new genus from off Hawaii. Exclusion of the latter from this clade is likely to be inaccurate because a) it has a mushroom-like body, only lacking the lateral outgrowths (hence we coded it as 0 for this character), b) it is similar in spiculation to *Rhabdopectella*^[[2]](#footnote-0)^, and c) a close relationship of this new genus to *Rhabdopectella* is also strongly supported by molecular evidence (this study). Thus, the Bayesian tree seems more accurate in grouping these latter two genera together (although they appear to be unrelated to the other core bolosomins on that tree). Inclusion of *Saccocalyx* in the core bolosomins as found here conflicts with molecular evidence that suggests a close relationship of that genus to *Hertwigia* (Corbitellinae), which has a completely different body shape but similar spicule complement [10]. Among the core bolosomins, *Bolosoma*, *Amphidiscella*, and *Vityaziella* form a clade, which is supported by the shared presence of codondiactins ("amphidiscs"; see File S4) in these genera. Within this clade, *Bolosoma* and *Amphidiscella* are most closely related. This latter grouping finds support from the presence of microdiscohexactins and codonstauractins. Spirodiscohexasters are interpreted as a synapomorphy of *Rhabdopectella* and *Saccocalyx*, implying convergent evolution of this spicule type in *Hertwigia*, which groups with *Placopegma, Chaunangium,* and *Caulocalyx* based on the shared presence of discoplumicomes. However, *Saccocalyx* also has discoplumicomes. Furthermore, molecular evidence suggests that spirodiscohexasters and discoplumicomes are homologous in *Hertwigia* and *Saccocalyx* (see above) whereas they evolved convergently in *Rhabdopectella*, which appears closely related to *Bolosoma* ([10]; this study). These results demonstrate the amount of conflicting phylogenetic signal present in the highly complex morphology of euplectellids and suggest that molecular data of additional genera will be indispensable for an accurate reconstruction of character evolution in this family.

Interestingly, all the venus-flower basket, or "iconic" euplectellids, are reconstructed as a clade by our analysis, which corresponds to the "Euplectellidae *s. str.*" of Mehl [12] (although she also included *Dictyocalyx*). This clade mostly includes members of Corbitellinae (*Corbitella*, *Dictyaulus*, *Heterotella*, *Ijimaiella*, *Pseudoplectella*, *Regadrella*), but also *Euplectella* and *Holascus* (Euplectellinae). We propose that this group of genera – hereafter the "Venus-Flower-Basket" or "VFB" clade^[[3]](#footnote-1)^ – should form the basis for future revisions of the suprageneric classification of Euplectellidae (see also discussion of total evidence results below and in the main text). However, the scope of this VFB clade is not entirely clear yet. First, *Acoelocalyx* and *Malacosaccus* (Euplectellinae) also have a venus-flower-basket-like body shape, although they lack the rigid framework with regularly spaced lateral oscula and a sieve plate. As they form the sister group to the VFB clade here – which is also consistent with molecular data ([11]; this study) – this lack could be primitive and the cylindrical body shape as such evolved first, before being modified into the fully-fleged euplectellid framework. Molecular evidence further suggests that some other genera that have a not-so-obvious venus-flower basket body shape – i.e., as tubular sacs without rigid framework and more irregularly spaced lateral oscula – should be included in the VFB clade, as is the case for *Docosaccus* and *Atlantisella* ([10, 11]; this study).

*Rossellidae (Lyssacinosida)*.—Rossellidae is here supported as monophyletic, including *Symplectella* (see [24]). In contrast to molecular evidence, which resolves *Symplectella* as deeply nested within Rossellidae ([24]; this study), the genus here appears as the sister group to the remainder of the family. Small dermalia supported by large hypodermal pentactins are known from all genera of Rossellidae except *Symplectella*, which is likely responsible for the early-branching position of *Symplectella* reconstructed here, i.e., hypodermalia are interpreted as primitively absent in that genus. However, hypodermalia are missing in some species of certain other rossellid genera (e.g., *Aulosaccus*), and given that *Symplectella* is monospecific, secondary loss seems the more plausible interpretation (see further discussion in [24]). It is noteworthy that small dermalia supported by large hypodermal pentactins also characterize all genera of Amphidiscophora, raising the question whether that feature was present in the ground plan of Hexactinellida and got lost multiple times or if it evolved convergently in Amphidiscophora and Rossellidae. Other characters supporting monophyly of Rossellidae include dermal stauractins, which are found in > 50% of rossellid genera and only occur in *Fieldingia* (Sceptrulophora) as a convergence, and dermal diactins, which do not occur outside of Rossellidae (although they are only known from eight genera).

Not surprisingly, subfamily Rossellinae, which is solely defined by negative features and has been shown by molecular analyses to be an artificial group [1], is not recovered as a clade here. In contrast, the recently resurrected subfamily Acanthascinae [25], i.e. *Acanthascus*, *Rhabdocalyptus*, and *Staurocalyptus*, is supported; the presence of discoctasters in these three genera provides an unambiguous synapomorphy. Within Acanthascinae, *Rhabdocalyptus* and *Staurocalyptus* are sister groups, which is supported by the occurence of dermal diactins and microscleres with floricoidal tips (in some species). However, the former also occur in six other rossellid genera and the latter are also found in *Leucopsacus* (Leucopsacidae), *Myliusia* and *Heterorete* (Euretidae), and many euplectellids.

*Caulophacus* and *Caulophacella* (formerly Rossellinae [26]) are firmly nested within Lanuginellinae, which supports their recent reallocation to that subfamily [27] with cladistic methods, and further corroborates molecular phylogenetic results [11]. Lanuginellinae was originally defined by the presence of strobiloplumicomes [26], which obviously got lost in *Caulophacus* and *Caulophacella* (see discussion in [11]). Another possible autapomorphy of Lanuginellinae *sensu* [27] is the loss of microdiscohexasters (which also convergently happened in *Aphorme*, *Bathydorus*, and *Trichasterina*). In Appendix 2 of the main text, we provide a revised diagnosis of Lanuginellinae.

Within Lanuginellinae, all genera except *Doconesthes* and *Mellonympha* form a clade characterized by a significant amount of hexactins among choanosomalia (also present in *Vitrollula*, likely as a convergence; but see results of total-evidence analyses in the main text). Within this clade, all genera except *Lanuginella* group together, an assemblage that is supported by hypoatrial pentactins (which however also convergently evolved in *Asconema*, *Crateromorpha*, and within Amphidiscophora) and synapticular fusion of megascleres (which also convergently evolved in *Crateromorpha* and outside Rossellidae, and is secondarily absent in *Caulophacella*). The remaining genera share a tubular peduncle, a character that is otherwise only present in *Crateromorpha* among Rossellidae (but also occurs in other lyssacinosidans; see above). Convergent evolution of peduncles in Rossellidae had already been suggested by early molecular work [1], and the present morphological analysis further discourages the taxonomic idea of grouping all pedunculate rossellids together ([26]: 1443). Finally, *Caulophacus*, *Caulophacella*, *Calycosoma*, and *Sympagella* form a clade that is (among Hexasterophora) unambiguously supported by pinular pentactins; the further resolution within this clade finds no obvious support from any characters.

Relationships between the remaining genera of Rossellidae are poorly resolved. *Anoxycalyx*, *Trichasterina*, and the new genus from off New Zealand group together, which is supported by the presence of trichasters, a spicule type restricted to these three genera. *Rossella*, *Scyphidium*, *Crateromorpha*, and *Vazella* form a clade that gains support from the presence of microdiscohexasters with unequal secondary ray lengths (anisodiscohexasters). *Rossella*, *Scyphidium,* and *Crateromorpha* further share the presence of discohexactins, which convergently evolved in *Caulophacus* (and in numerous non-rossellid genera). For the remaining resolution of the topology we could not determine any meaningful character support. Overall, except for monophyly of Lanuginellinae *sensu* [27], the topology of Rossellidae inferred here is largely incompatible with molecular phylogenies ([11]; this study).

**Total-evidence phylogeny of Hexasterophora (Figures 9-10) – Sceptrulophora, Euplectellidae, and Rossellidae**

*Sceptrulophora*.—Except for *Fieldingia*, which occupies the earliest branch (as in Figure 5; see previous section for discussion), a clade containing Uncinateridae (*Uncinatera* + *Tretopleura*; here reconstructed as monophyletic) and the three tretodictyids *Cyrtaulon*, *Sclerothamnus*, and *Tretocalyx* is the sister group to the remaining sceptrulophorans. Although sceptrules have so far not been described from the poorly known *Uncinatera*, the possession of uncinates and a similar (paraulocalycoid) framework construction as in the scopule-bearing *Tretopleura* support its inclusion in Sceptrulophora (see also Appendix 2). Specifically, monophyly of Uncinateridae is supported by the following framework characters: longitudinal strands formed by continuous extension, with overlapping continuous rays and uniaxial connecting beams; fusion of dictyonalia axon-to-ray, tip-to-tip, and tip-to-ray. While the combination of these features characterizes the paraulocalycoid pattern, only overlapping continuous rays are exclusive to Uncinateridae. All other characters convergently occur in other taxa, most notably Aulocalycidae and Auloplacidae. The different positions of Uncinateridae and Auloplacidae within Sceptrulophora and the placement of Aulocalycidae within Lyssacinosida (Figure 10) imply that the unusual framework architectures of Uncinateridae and Auloplacidae were derived independently from a more regular euretoid construction type, and that similarities to the aulocalycid architecture are entirely convergent developments (in strong contrast to the morphology-only phylogeny [Figure 5; see previous section]). A closer relationship of Uncinateridae to the three above-mentioned tretodictyids finds only weak morphological character support: loss of oxyhexasters, loss of atrial pentactins, and the evolution of skeletal channelization, all of which have occurred independently in several other taxa.

For the clade uniting all remaining sceptrulophorans, (sub)tyloscopules could be interpreted as an autapomorphy, although the phylogeny implies multiple independent losses. The topology within this clade seems to be driven largely by the molecular characters and for the deepest nodes morphological support remains unclear. One of the major subclades contains Tretodictyidae *s. str.* (see main text), which here also includes *Anomochone* and *Sclerothamnopsis*. Schizorhysial framework channelization is apomorphic for this group, although the topology recovered here implies convergent evolution of this character in *Cyrtaulon* and *Sclerothamnus* (for an in-depth discussion of tretodictyid characters, see previous section). However, the total-evidence tree (Figure 9) appears more parsimonious than the morphology-only tree (Figure 5) by being compatible with a diphyletic instead of supporting a triphyletic origin of Tretodictyidae *sensu* [20]. As in the morphology-only tree (Figure 5), the euretids *Gymnorete*, *Endorete*, and *Periphragella* are successive sister groups to Tretodictyidae *s. str.*, which finds some notable morphological support (see previous section). Sister to the above assemblage is a clade composed of the euretids *Pleurochorium* and *Eurete*, and *Laocoetis* (Craticulariidae) + *Stereochlamis* (Cribrospongiidae). A sister-group relationship of the latter two, paleontologically important (cf. [28]), relict taxa is also found in the morphology-only phylogeny (Figure 5) and is supported by the presence of diplorhysial channelization. A closer relationship of the two euretids to this clade is supported by the presence of oxyscopules, although these have also convergently evolved in *Aphrocallistes*.

The second major clade contains Auloplacidae (*Auloplax* + *Dictyoplax*; see previous section) and the molecularly supported *Homoieurete* + *Sarostegia* clade (see main text) as successive sister groups to a clade that unites Aphrocallistidae, Farreidae, and a number of euretids, including the sequenced genera *Conorete*, *Verrucocoeloidea*, and *Lefroyella* (see [16]). *Pararete* and *Pityrete* are more closely related to Aphrocallistidae, whereas the remaining euretids group around Farreidae. The placement of *Pararete* and *Pityrete* is supported by the occurrence of diarhysial channelization (also convergently present in *Anomochone* and *Uncinatera*); a cylindrical (honey-comb) shape of the diarhyses is an unambiguous autapomorphy of Aphrocallistidae (*Aphrocallistes* + *Heterochone*; see also previous section). Monophyly of Farreidae is supported by the typical "farreoid" framework construction and the presence of clavules instead of scopules (see [7]). Farreoid frameworks can also be found in the euretids *Bathyxiphus* and *Conorete* (see previous section), but in contrast to the morphology-only tree (Figure 5), where this was interpreted as a synapomorphy, the total-evidence tree (Figure 9) implies convergent evolution of this feature in Farreidae and *Bathyxiphus* + *Conorete*, because Farreidae appears more closely related to the remaining five than to these two genera. As in the molecular phylogenies (Figure 7, Figure S9), *Verrucocoeloidea* and *Lefroyella* are related, but here the unsequenced genera *Pinulasma*, *Tretochone*, and *Chonelasma* are also included in this clade. However, we could not find any meaningful morphological support for this grouping.

*Euplectellidae*.—As in the morphology-only analysis (Figure 6; see previous section), the total-evidence analysis recovered a clade of all genera with the typical venus-flower-basket body shape featuring longitudinal and circular skeleton beams leaving regularly arrayed parietal oscula, as well as sieve plates covering the main osculum (the latter character has evolved convergently in *Placopegma* as well as several times outside of Euplectellidae; see [24]). Hereafter we refer to this group as the "venus-flower-basket (VFB) clade" (similar to Euplectellidae *s. str.* of [12]; see previous section). Consistent with the morphology-only analysis (Figure 6), the VFB clade is sister to *Malacosaccus* + *Acoelocalyx*, which share some similarities in body shape (see previous section). *Atlantisella*, which appears closely related to the VFB genera *Euplectella* and *Regadrella* in the molecular phylogenies (e.g., Figure 7), is here reconstructed as the sister group of the VFB + *Malacosaccus* + *Acoelocalyx* clade, followed by *Docosaccus*. *Atlantisella* and *Docosaccus* are tubular sacs without rigid frameworks and with more irregularly spaced lateral oscula. This body shape can be interpreted as derived from or ancestral to the venus-flower basket, so we refer to the group uniting all the above taxa as VFB *s. l*. Within the VFB clade, the only subclade consistent with Figure 6 is *Euplectella* + *Holascus* + *Dictyaulus*, which seems morphologically well-supported (see previous section). Sister to these three genera is *Heterotella*, which shares sigmato- or drepanocomes with them. However, these spicules also occur outside the VFB clade. The same applies to graphiocomes, which within the VFB clade unite all genera except *Pseudoplectella*, but are also present in *Atlantisella* and in several genera outside VFB *s. l*. For the exact placement of *Corbitella*, *Regadrella*, and *Ijimaiella*, we could not find any meaningful morphological character support.

Sister group to VFB *s. l.* is a clade of seven genera that includes *Walteria* + *Dictyocalyx* plus a clade uniting all taxa with discoplumicomes. Thus, compared to the morphology-only analysis (Figure 6), addition of the molecular data resulted in a more parsimonious scenario where this peculiar spicule type only evolved once. This finding might provide another source of inspiration for future revisions of the euplectellid subfamily-classification, i.e. similar to Lanuginellinae and Acanthascinae within Rossellidae, certain types of microscleres might be used to delimit groups of genera. Within the discoplumicome-bearing clade, loss of hexactine dermalia might be a synapomorphy of *Placopegma*, *Chaunangium*, and *Caulocalyx* (convergent only in *Atlantisella* among Euplectellidae). As discussed previously ([10]; see also previous section), the sequenced genera *Hertwigia* and *Saccocalyx*, despite having very different body shapes, share a similar microsclere composition (although no spicule type alone is exclusive to these two genera); we might here add complete loss of pentactins as a proper synapomorphy that, within Anuncinataria, only happened twice convergently according to our results (in *Neocaledoniella* and *Cyathella*).

*Walteria* and *Dictyocalyx* share discasters and graphiocomes, but these spicules are also widespread elsewhere. However, a very close relationship of these two genera has been recognized previously on the basis of overall spicule combinations [23, 29]. A closer relationship of *Walteria* + *Dictyocalyx* to the discoplumicome-bearing clade finds no clear-cut character support; instead we suspect that with increased taxon sampling for the molecular partition these two genera will turn out to be related to the VFB *s. l.* clade as they also have more or less cylindrical body shapes with numerous lateral oscula.

The sister group to all the above groups is a clade composed of the majority of Bolosominae (Bolosominae *s. str.* hereafter), a subfamily characterized by a mushroom-like body shape with tubular peduncles [23]. Besides the peduncle, loss of oxyhexactins might be a proper autapomorphy of Bolosominae *s. str.*, although these spicules have also been lost in seven other genera within Euplectellidae according to our results (besides from that, oxyhexactins might actually have evolved convergently multiple times, so their absence in Bolosominae *s. str.* might be primitive instead; see main text). In contrast to the "core bolosomin" clade recovered from the morphological data matrix (see previous section), Bolosominae *s. str.* includes *Hyalostylus* and the new genus from off Hawaii, but excludes *Saccocalyx* (see above). These results suggest that the mushroom-like body shape in *Saccocalyx* evolved convergently and that lateral outgrowths on the main body were secondarily reduced in the new genus and in *Hyalostylus*. As in Figure 6, the poorly known, probably pedunculate, *Caulocalyx* is here also excluded from Bolosominae on the basis of it having discoplumicomes (see above). Thus, tubular peduncles appear to have evolved three times independently in Euplectellidae: once in the LCA of Bolosominae *s. str.*, and twice independently within the discoplumicome-bearing clade. Within Bolosominae *s. str.*, *Neocaledoniella* is sister to the well-supported, codondiactin-bearing *Bolosoma* + *Amphidiscella* + *Vityaziella* group (see previous section), which finds some support from the secondary absence of atrial pentactins in these four genera. The remaining four genera also form a clade, support for which remains somewhat elusive. Within this clade, *Rhabdopectella* and *Hyalostylus* share the presence of spiroxyhexasters (convergent to *Amphidiscella*) and the secondary absence of graphiocomes (convergent to *Amphidiscella* + *Bolosoma*).

In summary, our total-evidence analysis supports three major clades within Euplectellidae that might form the basis of future revisions of its subfamilial division: 1) a group uniting all genera with the iconic venus-flower-basket body shape plus some genera where this type of body shape is not so obvious but can still be recognized (i.e., *Acoelocalyx*, *Malacosaccus*, *Docosaccus*, *Atlantisella*, and possibly *Walteria* and *Dictyocalyx*); 2) a group uniting all genera with discoplumicomes, and 3) Bolosominae Tabachnick, 2002 with a revised scope and diagnosis to exclude discoplumicome-bearing genera.

*Rossellidae*.—As discussed in the main text, Rossellidae is divided into Lanuginellinae and a clade of mostly microdiscohexaster-bearing genera. Here we discuss the internal relationships of these clades. Within Lanuginellinae, *Sympagella* + *Calycosoma* form the sister group of the remaining genera, and *Doconesthes* + *Mellonympha* group together; the other genera branch off successively between these two clades. In accordance with the molecular phylogeny, *Caulophacus* and *Caulophacella* are not closely related, implying that strobiloplumicomes were lost independently in these two taxa. Morphological support for the Lanuginellinae topology recovered here is rather weak and mostly involves losses of characters inferred to be present in the ground pattern of the subfamily: pinular pentactins in the LCA of *Lanugonychia* and *Doconesthes*, synapticular fusion and hypoatrial pentactins in the LCA of *Lanuginella* and *Doconesthes* (the former also convergently lost in *Caulophacella*), and a tubular peduncle in the LCA of *Lophocalyx* and *Doconesthes*. The only positive character is the presence of tauactin megascleres, which is inferred as a synapomorphy of *Lanugonychia*, *Lophocalyx*, *Lanuginella*, *Mellonympha*, and *Doconesthes*. However, this spicule type is widespread within Lyssacinosida and has apparently evolved multiple times convergently, including in other rossellids. Within the microdiscohexaster clade, *Crateromorpha*, *Scyphidium*, and *Vazella* group together to the exclusion of all other genera. This assemblage is supported by anisodiscohexasters, which however also convergently evolved in *Rossella* according to the topology of Figure 10. In concordance with the molecular phylogeny, Acanthascinae is the sister group of *Nodastrella* + *Aulosaccus*. This clade gains some morphological support from the presence of discasters, although these spicules are (secondarily) absent in *Staurocalyptus* and are also found in the unrelated *Asconema* and *Caulophacus*, as well as outside Rossellidae. As in the morphology-only tree (Figure 6), the new genus from off New Zealand forms a clade with the other two trichaster-bearing genera *Anoxycalyx* and *Trichasterina* (see previous section). *Rossella* and *Symplectella* are reconstructed as sister groups, which mainly finds support from the presence of calycocomes in these two genera, although these spicules also occur elsewhere (see discussions in [24, 30]). For the remaining, especially deeper, nodes within the microdiscohexaster clade we could not find any meaningful morphological character support.

**References**

1. Dohrmann M, Janussen D, Reitner J, Collins AG, Wörheide G. Phylogeny and evolution of glass sponges (Porifera, Hexactinellida). Syst Biol*.* 2008;57:388-405.

2. Tabachnick KR, Menshenina LL. Family Pheronematidae Gray, 1870. In: Hooper JNA, van Soest RWM, editors. Systema Porifera. A Guide to the Classification of Sponges. New York: Plenum; 2002. p. 1267-1280.

3. Tabachnick KR. Family Monorhaphididae Ijima, 1927. In: Hooper JNA, van Soest RWM, editors. Systema Porifera. A Guide to the Classification of Sponges. New York: Plenum; 2002. p. 1264-1266.

4. Tabachnick KR, Menshenina LL. An approach to the phylogenetic reconstruction of Amphidiscophora (Porifera: Hexactinellida). Mem Qld Mus*.* 1999;44:607-615.

5. Tabachnick KR, Menshenina LL. Family Hyalonematidae Gray, 1857. In: Hooper JNA, van Soest RWM, editors. Systema Porifera. A Guide to the Classification of Sponges. New York: Plenum; 2002. p. 1232-1263.

6. Özdikmen H. *Tabachnickia* nom. nov., a new name for the preoccupied sponge genus *Platella* Tabachnick, 1988 (Porifera: Hexactinellida). Mun Ent Zool*.* 2010;5:297-298.

7. Dohrmann M, Göcke C, Janussen D, Reitner J, Lüter C, Wörheide G. Systematics and spicule evolution in dictyonal sponges (Hexactinellida: Sceptrulophora) with description of two new species. Zool J Linn Soc*.* 2011;163:1003-1025.

8. Wörheide G, Dohrmann M, Erpenbeck D, Larroux C, Maldonado M, Voigt O, Borchiellini C, Lavrov DV. Deep phylogeny and evolution of sponges (Phylum Porifera). Adv Mar Biol*.* 2012;61:1-78.

9. Reiswig HM, Wheeler B. Family Euretidae Zittel, 1877. In: Hooper JNA, van Soest RWM, editors. Systema Porifera. A Guide to the Classification of Sponges. New York: Plenum; 2002. p. 1301-1331.

10. Dohrmann M, Collins AG, Wörheide G. New insights into the phylogeny of glass sponges (Porifera, Hexactinellida): monophyly of Lyssacinosida and Euplectellinae, and the phylogenetic position of Euretidae. Mol Phylogenet Evol*.* 2009;52:257-262.

11. Dohrmann M, Haen KM, Lavrov DV, Wörheide G. Molecular phylogeny of glass sponges (Porifera, Hexactinellida): increased taxon sampling and inclusion of the mitochondrial protein-coding gene, cytochrome oxidase subunit I. Hydrobiologia*.* 2012;687:11-20.

12. Mehl D. Die Entwicklung der Hexactinellida seit dem Mesozoikum. Paläobiologie, Phylogenie und Evolutionsökologie. Berl Geowiss Abh E*.* 1992;2:1-164.

13. Reiswig HM. Family Uncinateridae fam. nov. In: Hooper JNA, van Soest RWM, editors. Systema Porifera. A Guide to the Classification of Sponges. New York: Plenum; 2002. p. 1372-1376.

14. Reiswig HM. Family Aulocalycidae Ijima, 1927. In: Hooper JNA, van Soest RWM, editors. Systema Porifera. A Guide to the Classification of Sponges. New York: Plenum; 2002. p. 1362-1371.

15. Reiswig HM, Kelly M. The Marine Fauna of New Zealand: Hexasterophoran Glass Sponges of New Zealand (Porifera: Hexactinellida: Hexasterophora): Orders Hexactinosida, Aulocalycoida and Lychniscosida. NIWA Biodiv Mem*.* 2011;124:1-176.

16. Reiswig HM, Dohrmann M. Three new species of glass sponges (Porifera: Hexactinellida) from the West Indies, and molecular phylogenetics of Euretidae and Auloplacidae (Sceptrulophora). Zool J Linn Soc*.* 2014;171:233-253.

17. Topsent E. *Sarostegia oculata*, Hexactinellide nouvelle des îles du Cap-Vert. Bull Mus Océanogr Monaco*.* 1904;10:1-8.

18. Reiswig HM. Family Farreidae Gray, 1872. In: Hooper JNA, van Soest RWM, editors. Systema Porifera. A Guide to the Classification of Sponges. New York: Plenum; 2002. p. 1332-1340.

19. Duplessis K, Reiswig HM. Three new species and a new genus of Farreidae (Porifera: Hexactinellida: Hexactinosida). Proc Biol Soc Wash*.* 2004;117:199-212.

20. Reiswig HM. Family Tretodictyidae Schulze, 1886. In: Hooper JNA, van Soest RWM, editors. Systema Porifera. A Guide to the Classification of Sponges. New York: Plenum; 2002. p. 1341-1354.

21. Tabachnick KR. Family Leucopsacidae Ijima, 1903. In: Hooper JNA, van Soest RWM, editors. Systema Porifera. A Guide to the Classification of Sponges. New York: Plenum; 2002. p. 1435-1440.

22. Reiswig HM. Order Lyssacinosida Zittel, 1877. In: Hooper JNA, van Soest RWM, editors. Systema Porifera. A Guide to the Classification of Sponges. New York: Plenum; 2002. p. 1387.

23. Tabachnick KR. Family Euplectellidae Gray, 1867. In: Hooper JNA, van Soest RWM, editors. Systema Porifera. A Guide to the Classification of Sponges. New York: Plenum; 2002. p. 1388-1434.

24. Dohrmann M. *Symplectella rowi* (Porifera: Hexactinellida: Lyssacinosida) is a rossellid, not a euplectellid. J Mar Biol Ass UK*.* 2016;96:291-295.

25. Reiswig HM, Stone RP. New glass sponges (Porifera: Hexactinellida) from deep waters of the central Aleutian Islands, Alaska. Zootaxa*.* 2013;3628:1-64.

26. Tabachnick KR. Family Rossellidae Schulze, 1885. In: Hooper JNA, van Soest RWM, editors. Systema Porifera. A Guide to the Classification of Sponges. New York: Plenum; 2002. p. 1441-1505.

27. Boury-Esnault N, Vacelet J, Reiswig HM, Fourt M, Aguilar R, Chevaldonné P. Mediterranean hexactinellid sponges, with the description of a new *Sympagella* species (Porifera, Hexactinellida). J Mar Biol Ass UK*.* 2015;95:1353-1364.

28. Krautter M. Fossil Hexactinellida: an overview. In: Hooper JNA, van Soest RWM, editors. Systema Porifera. A Guide to the Classification of Sponges. New York: Plenum; 2002. p. 1211-1223.

29. Tabachnick KR, Lévi C. Lyssacinosa du Pacifique sud-ouest (Porifera: Hexactinellida). In: Marshall B, Richer de Forges B, editors. Tropical Deep-Sea Benthos 23. Mém. Mus. Natl. Hist. Nat. 191; 2004. p. 11-71.

30. Dohrmann M, Göcke C, Reed J, Janussen D. Integrative taxonomy justifies a new genus, *Nodastrella* gen. nov., for North Atlantic "*Rossella*" species (Porifera: Hexactinellida: Rossellidae). Zootaxa*.* 2012;3383:1-13.

1. *Tretopleura* (Uncinateridae) is the only known aulocalycoid genus with sceptrules, and thus should be moved from Aulocalycoida to Sceptrulophora [7, 8], which is also supported by molecular data (this study) [↑](#footnote-ref--1)
2. *Rhabdopectella* is currently classified in Corbitellinae, but we have definite proof that it belongs in Bolosominae (Reiswig and Dohrmann, unpubl. obs.) [↑](#footnote-ref-0)
3. The term Euplectellidae *s. str.* proposed by Mehl [12] appears inappropriate because only a minority (8 of 27) of euplectellid genera belong to this clade; therefore they are not typical (euplectellids in the strict sense), but rather highly specialized. [↑](#footnote-ref-1)
