# Supplementary figures and images for: An integrative systematic framework helps to reconstruct skeletal evolution of glass sponges (Porifera, Hexactinellida)
Source: Front Zool. 2017 Mar 21;14:18. doi: 10.1186/s12983-017-0191-3 (PMC5359874; doi:10.1186/s12983-017-0191-3)

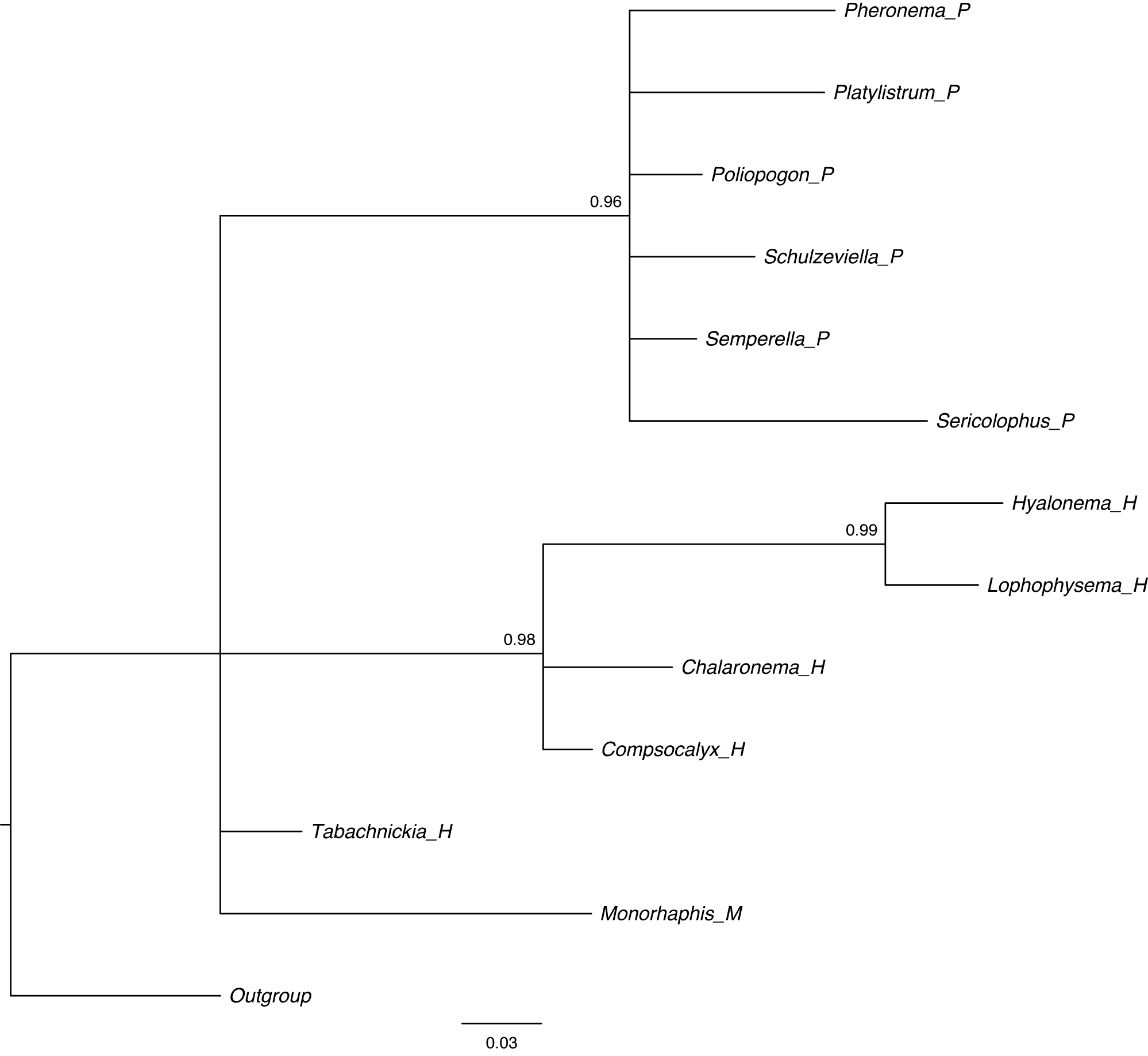

Supplement: Additional file 15: Figure S1. — Phylogeny of Amphidiscophora inferred from the morphological data matrix with MrBayes. 50% majority rule consensus tree from 9000 post-burnin samples. Average standard deviation of split frequencies between two independent runs was 0.003873. Bayesian posterior probabilities <1.00 shown on branches. H, Hyalonematidae; M, Monorhaphididae; P, Pheronematidae. Scale bar, expected number of character replacements per character. (JPG 222 KB) [file 12983_2017_191_MOESM15_ESM.jpg]

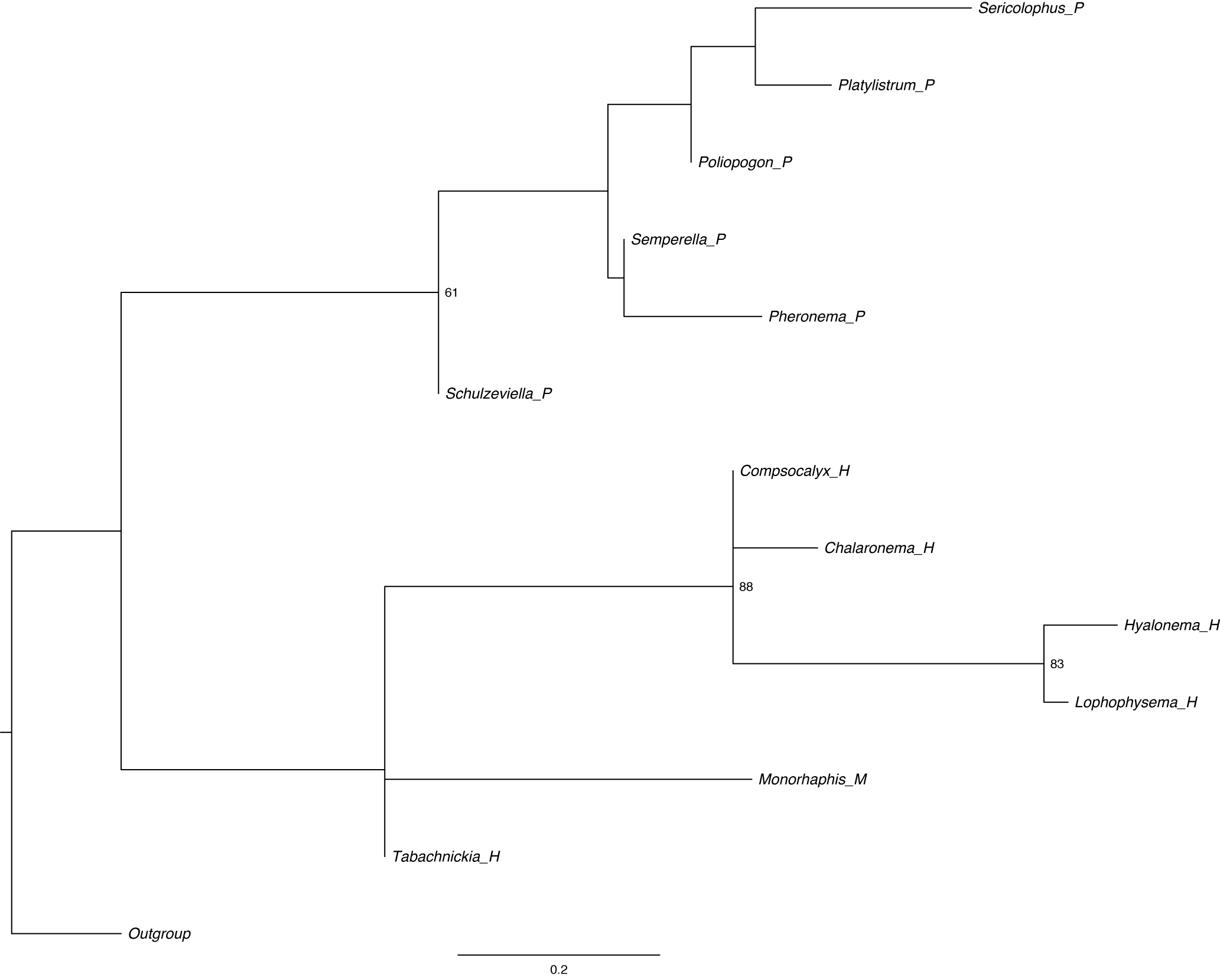

Supplement: Additional file 16: Figure S2. — Phylogeny of Amphidiscophora inferred from the morphological data matrix with RAxML. Bootstrap values >50% shown on branches (based on 1000 pseudoreplicates). H, Hyalonematidae; M, Monorhaphididae; P, Pheronematidae. Scale bar, expected number of character replacements per character. (JPG 179 KB) [file 12983_2017_191_MOESM16_ESM.jpg]

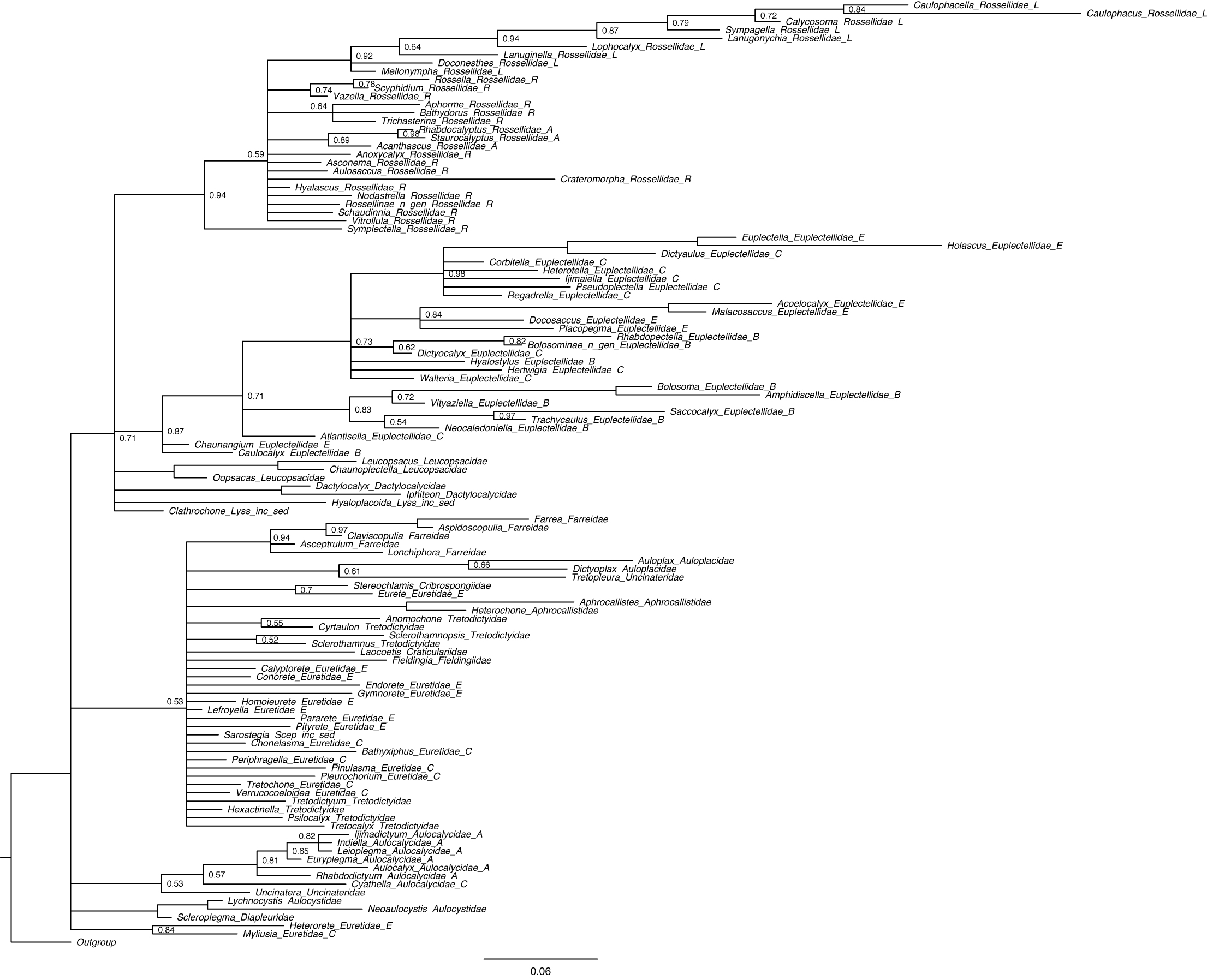

Supplement: Additional file 17: Figure S3. — Phylogeny of Hexasterophora inferred from the morphological data matrix with MrBayes. 50% majority rule consensus tree from 9000 post-burnin samples. Average standard deviation of split frequencies between two independent runs was 0.026249. Bayesian posterior probabilities <1.00 shown on branches. Current family assignment given after genus names. Subfamilies indicated with letters: A = Acanthasci-nae (Rossellidae)/Aulocalycinae (Aulocalycidae), B = Bolosominae (Euplectelli-dae), C = Corbitellinae (Euplectellidae)/Chonelasmatinae (Euretidae)/Cyathellinae (Aulocalycidae), E = Euplectellinae (Euplectellidae)/Euretinae (Euretidae), L = Lanuginellinae (Rossellidae), R = Rossellinae (Rossellidae). Scale bar, expected number of character replacements per character. (JPG 714 KB) [file 12983_2017_191_MOESM17_ESM.jpg]

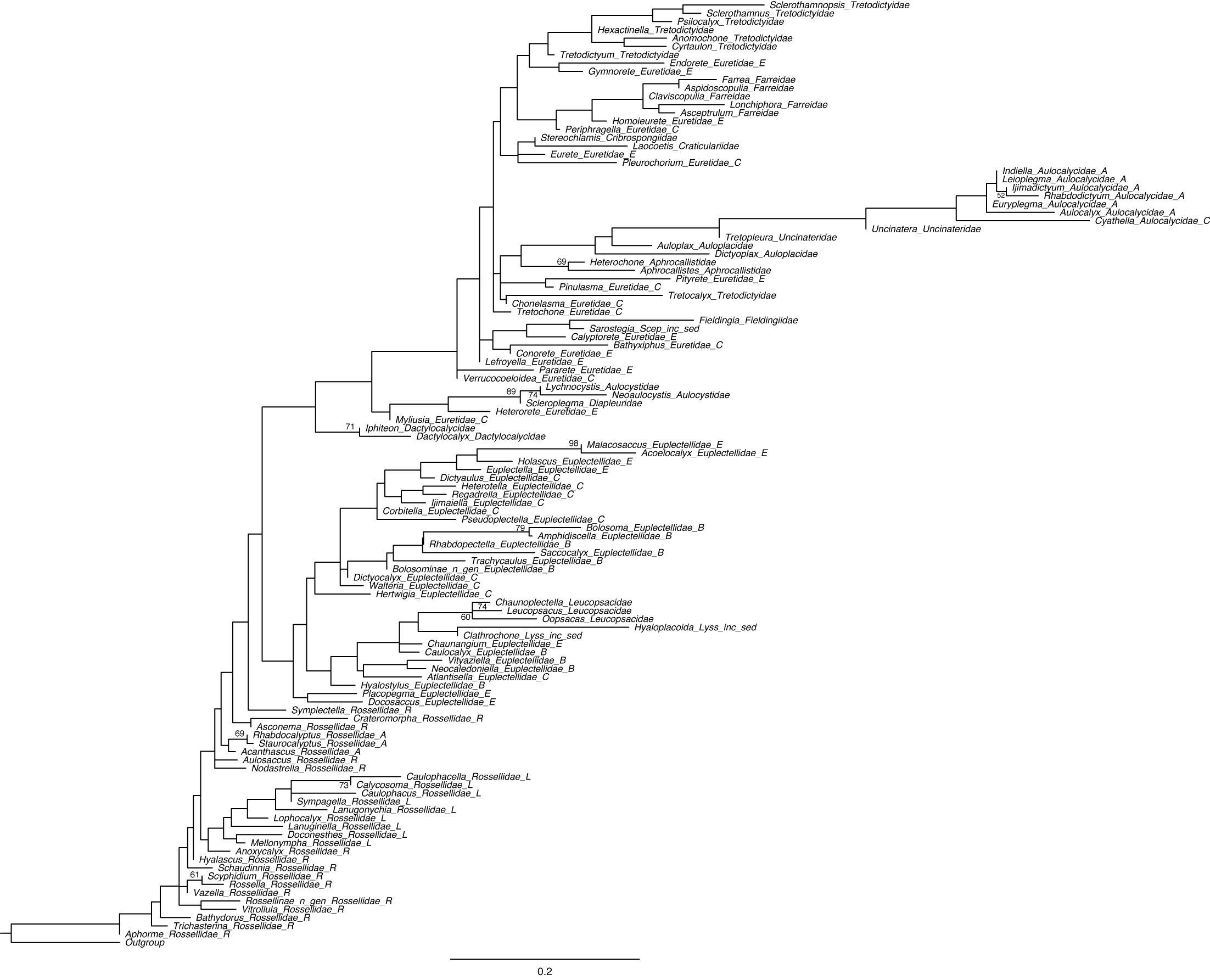

Supplement: Additional file 18: Figure S4. — Phylogeny of Hexasterophora inferred from the morphological data matrix with RAxML. Bootstrap values >50% shown on branches (based on 650 pseudoreplicates). Current family assignment given after genus names. Subfamilies indicated with letters: A = Acanthascinae (Rossellidae)/Aulocalycinae (Aulocalycidae), B = Bolosominae (Euplectellidae), C = Corbitellinae (Euplectellidae)/Chonelasmatinae (Euretidae)/Cyathellinae (Aulocalycidae), E = Euplectellinae (Euplectellidae)/Euretinae (Euretidae), L = Lanuginellinae (Rossellidae), R = Rossellinae (Rossellidae). Scale bar, expected number of character replacements per character. (JPG 645 KB) [file 12983_2017_191_MOESM18_ESM.jpg]

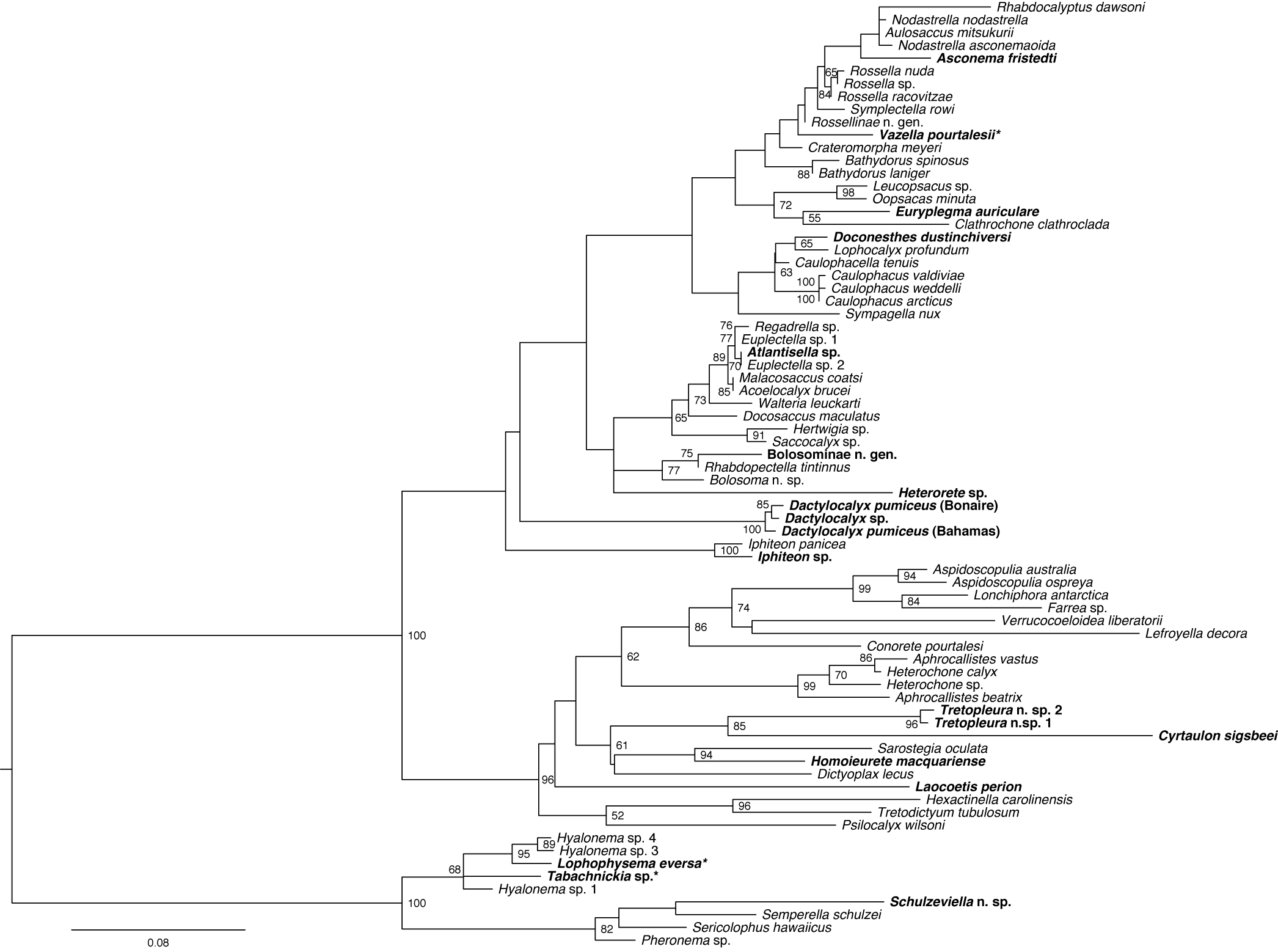

Supplement: Additional file 19: Figure S5. — Mitochondrial 16S rDNA phylogeny of Hexactinellida inferred with RAxML. Bootstrap values >50% shown on branches (based on 450 pseudoreplicates). Newly sampled species highlighted in bold. *, sequence data from mitochondrial genome sequencing projects [72, 73]. Scale bar, expected number of substitutions per site. (JPG 523 KB) [file 12983_2017_191_MOESM19_ESM.jpg]

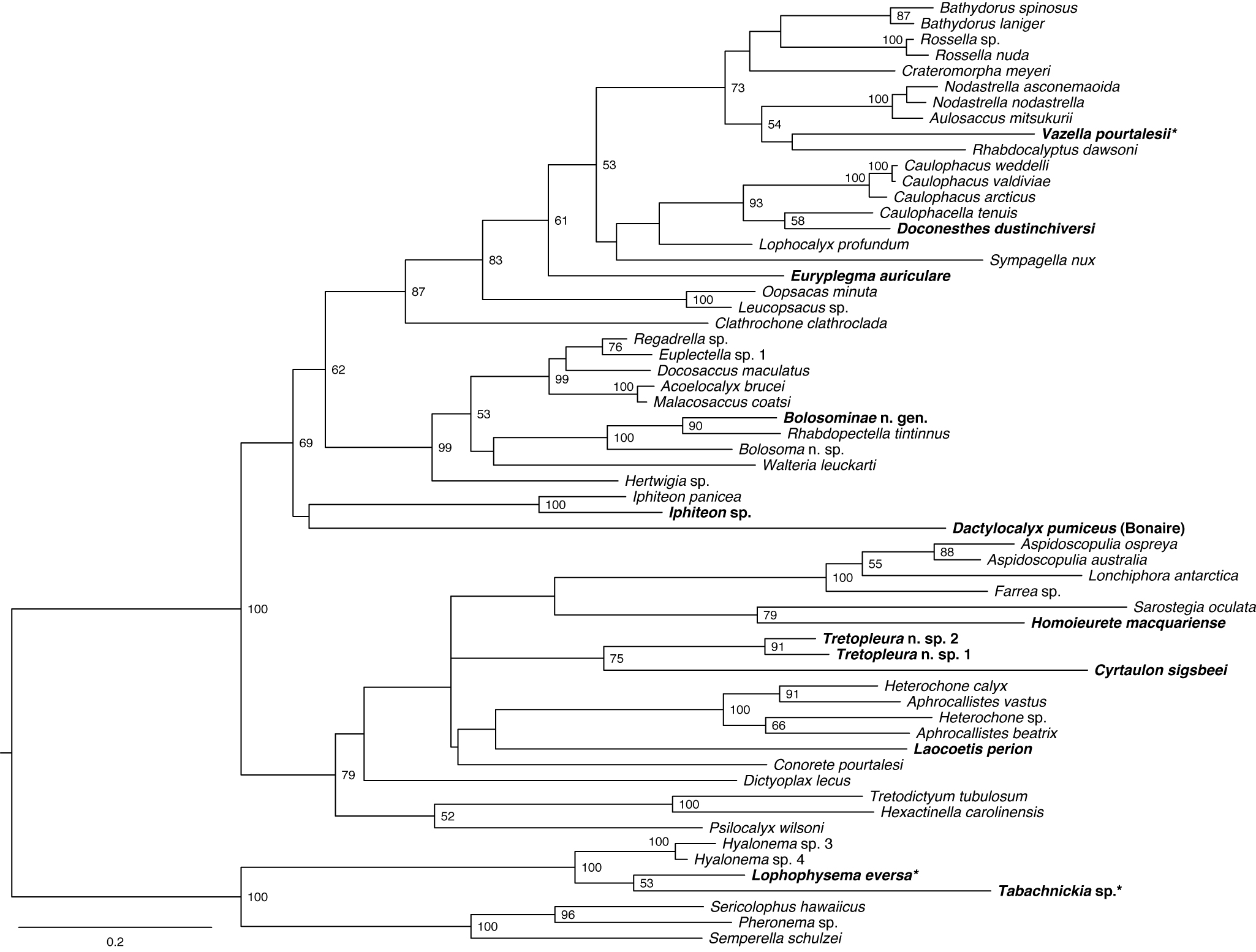

Supplement: Additional file 20: Figure S6. — Mitochondrial COI phylogeny of Hexactinellida inferred with RAxML from nucleotide alignment. Bootstrap values >50% shown on branches (based on 450 pseudoreplicates). Newly sampled species highlighted in bold. *, sequence data from mitochondrial genome sequencing projects [72, 73]. Scale bar, expected number of substitutions per site. (JPG 543 KB) [file 12983_2017_191_MOESM20_ESM.jpg]

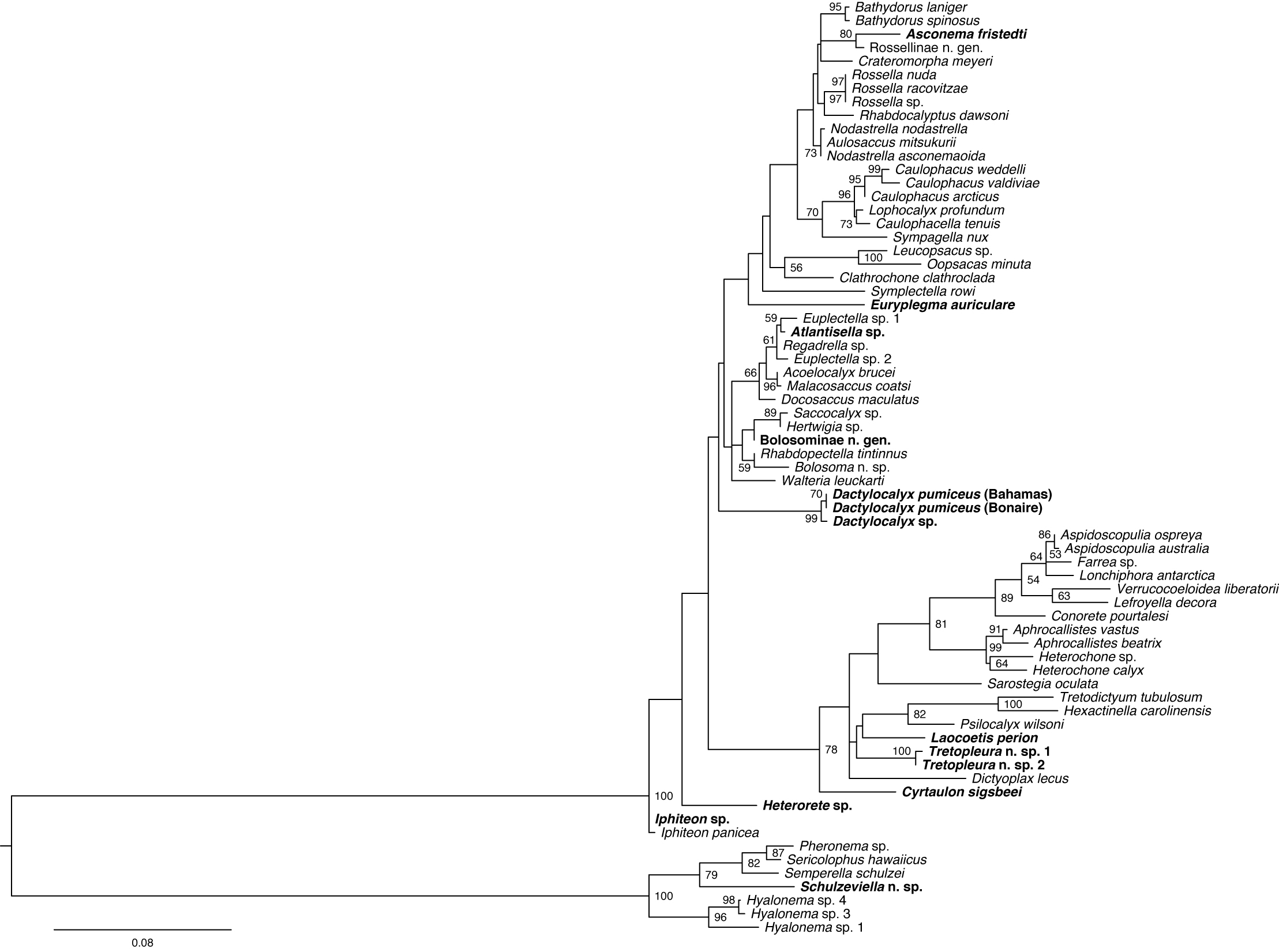

Supplement: Additional file 21: Figure S7. — 28S rDNA phylogeny of Hexactinellida inferred with RAxML. Bootstrap values >50% shown on branches (based on 600 pseudoreplicates). Newly sampled species highlighted in bold. Scale bar, expected number of substitutions per site. (JPG 514 KB) [file 12983_2017_191_MOESM21_ESM.jpg]

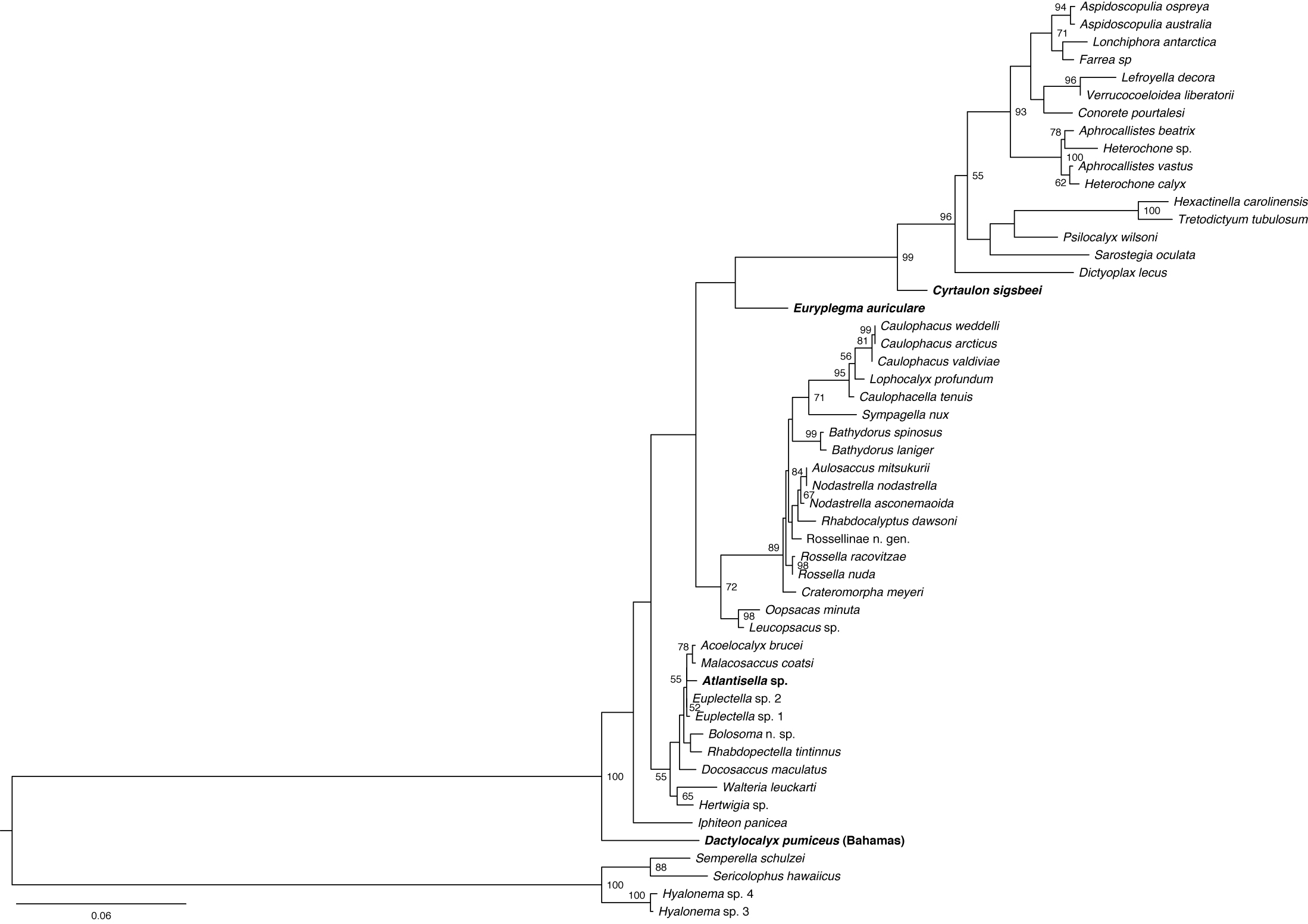

Supplement: Additional file 22: Figure S8. — 18S rDNA phylogeny of Hexactinellida inferred with RAxML. Bootstrap values >50% shown on branches (based on 500 pseudoreplicates). Newly sampled species highlighted in bold. Scale bar, expected number of substitutions per site. (JPG 368 KB) [file 12983_2017_191_MOESM22_ESM.jpg]

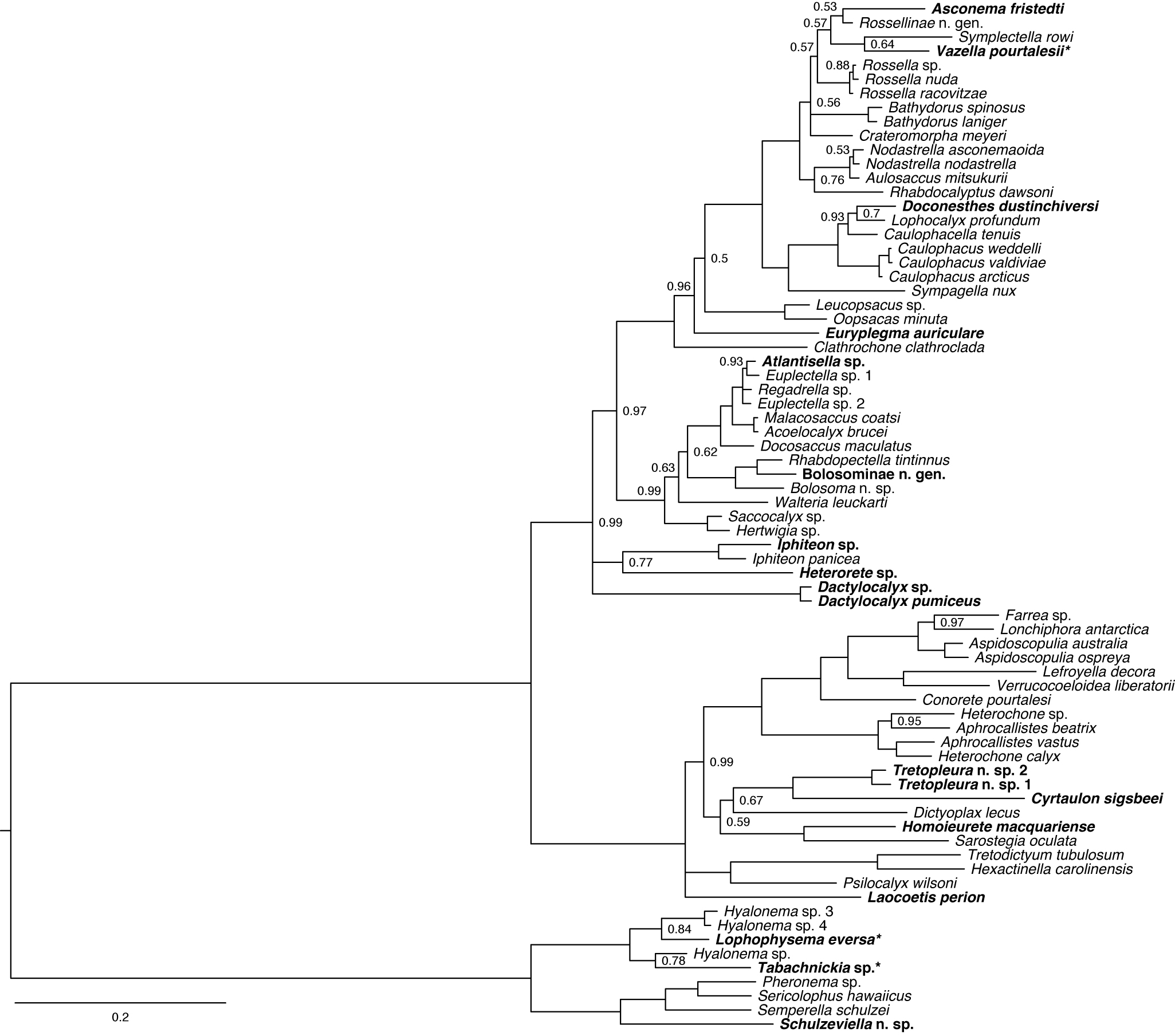

Supplement: Additional file 23: Figure S9. — Phylogeny of Hexactinellida inferred from concatenated molecular markers with MrBayes. 50% majority rule consensus tree from 25,000 post-burnin samples. Average standard deviation of split frequencies between two independent runs was 0.003858. Bayesian posterior probabilities <1.00 shown on branches. Newly sampled species highlighted in bold. *, 16S and COI sequence data from mitochondrial genome sequencing projects [72, 73]. Scale bar, expected number of substitutions per site. (JPG 615 KB) [file 12983_2017_191_MOESM23_ESM.jpg]

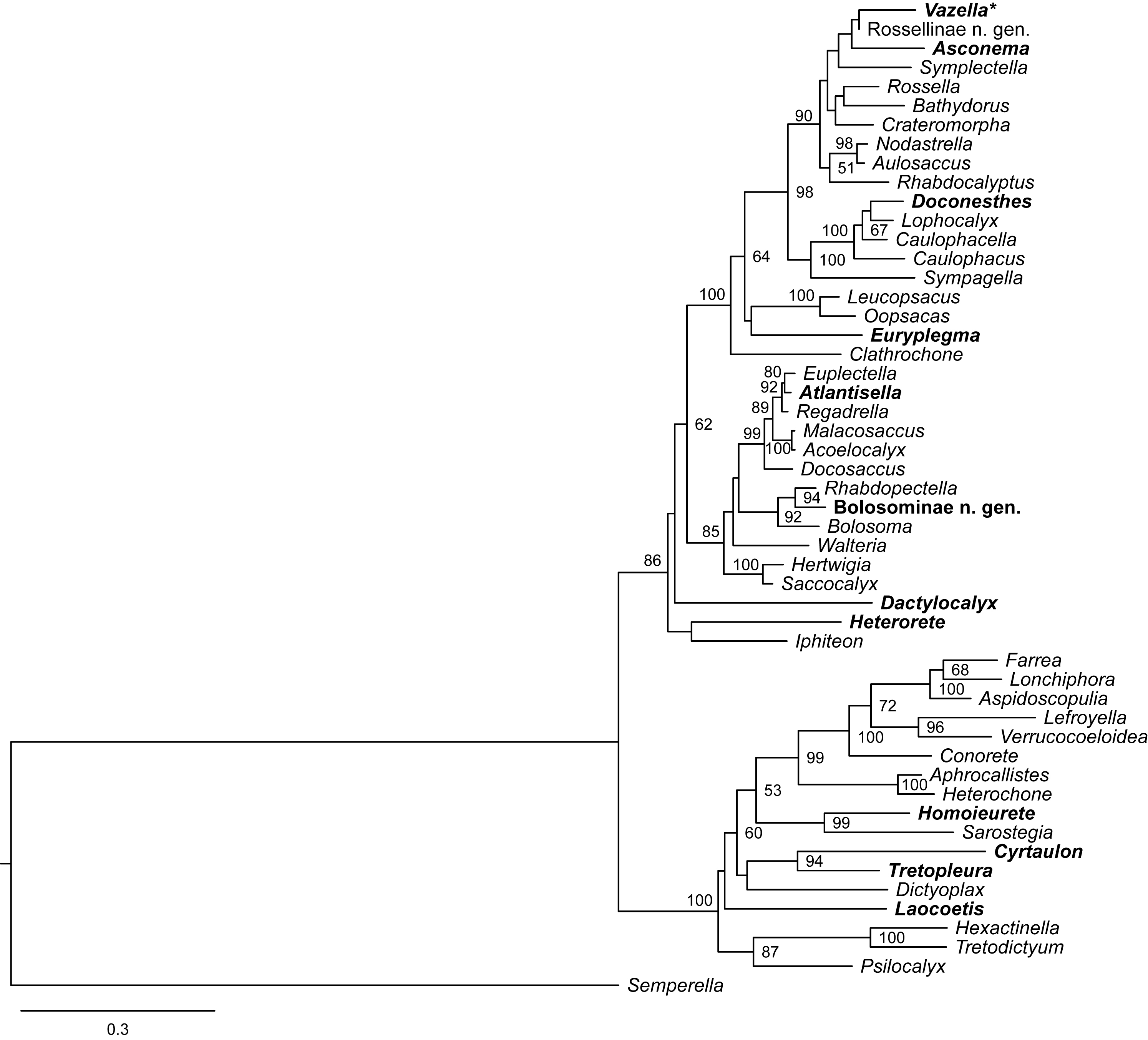

Supplement: Additional file 24: Figure S10. — Phylogeny of Hexasterophora inferred with RAxML from reduced taxon set (one species per genus) of concatenated molecular markers. Bootstrap values >50% shown on branches (based on 450 pseudoreplicates). Newly sampled genera highlighted in bold. *, 16S and COI sequence data from mitochondrial genome sequencing project [72]. Scale bar, expected number of substitutions per site. (JPG 843 KB) [file 12983_2017_191_MOESM24_ESM.jpg]

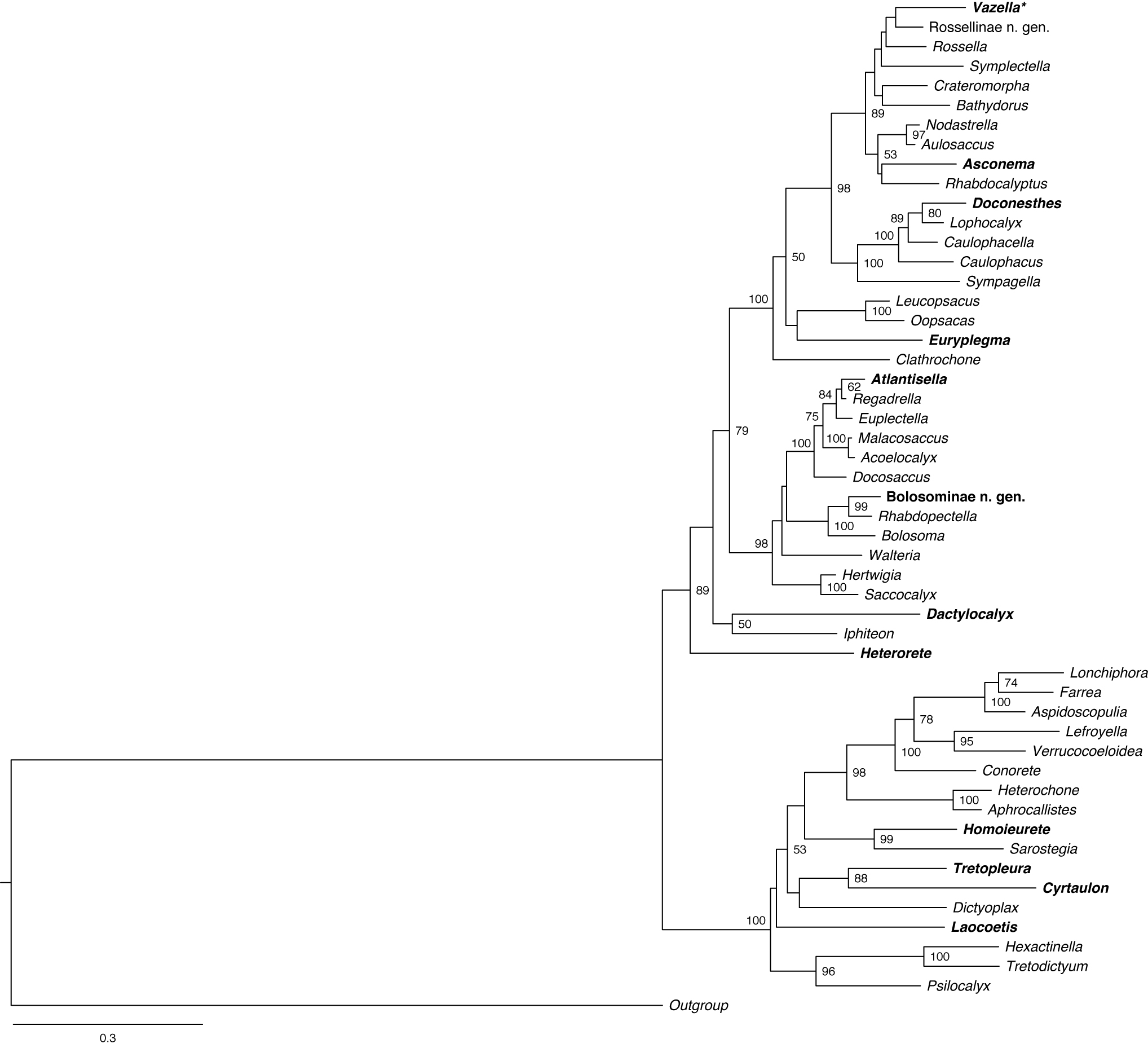

Supplement: Additional file 25: Figure S11. — Phylogeny of Hexasterophora inferred with RAxML from concatenated molecular and morphological data, including only genera with sequence data. Bootstrap values >50% shown on branches (based on 450 pseudoreplicates). Newly sampled genera highlighted in bold. *, 16S and COI sequence data from mitochondrial genome sequencing project [72]. Scale bar, expected number of substitutions/character replacements per site/character. (JPG 378 KB) [file 12983_2017_191_MOESM25_ESM.jpg]

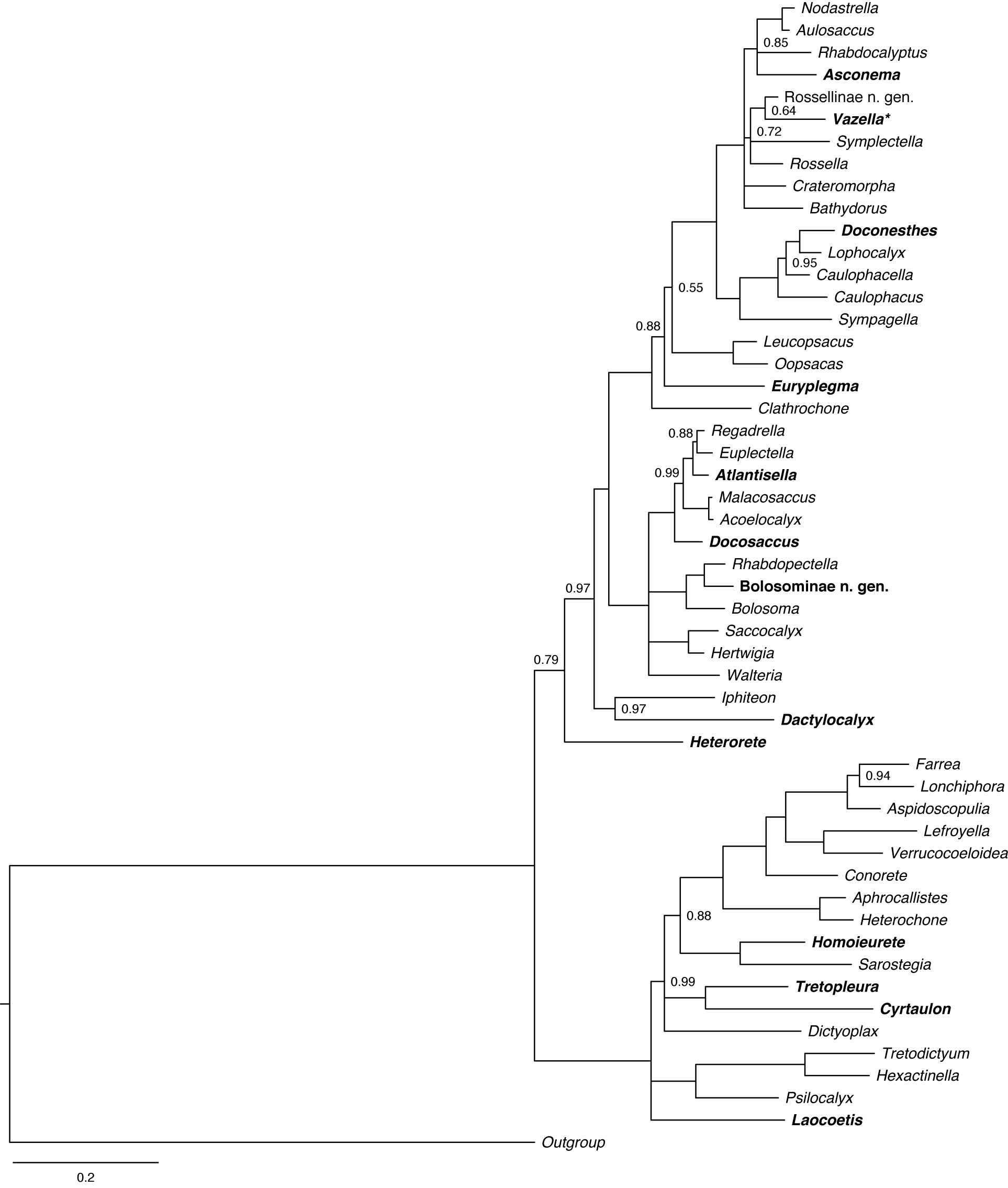

Supplement: Additional file 26: Figure S12. — Phylogeny of Hexasterophora inferred with MrBayes from concatenated molecular and morphological data, including only genera with sequence data. Fifty percent majority rule consensus tree from 45,000 post-burnin samples. Average standard deviation of split frequencies between two independent runs was 0.002564. Bayesian posterior probabilities <1.00 shown on branches. Newly sampled genera highlighted in bold. *, 16S and COI sequence data from mitochondrial genome sequencing project [72]. Scale bar, expected number of substitutions/character replacements per site/character.(JPG 422 KB) [file 12983_2017_191_MOESM26_ESM.jpg]

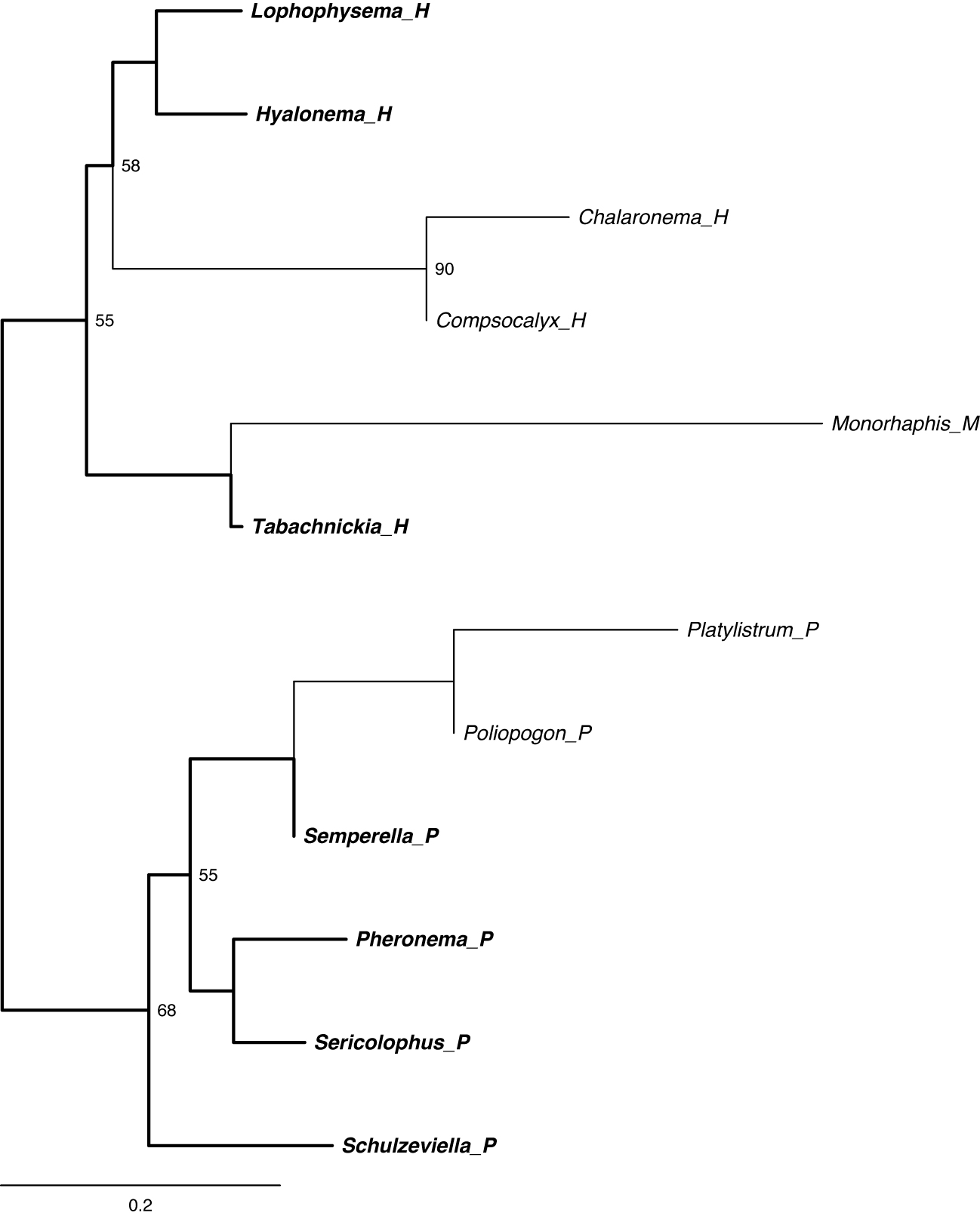

Supplement: Additional file 27: Figure S14. — Phylogeny of Amphidiscophora inferred with MrBayes from concatenated molecular and morphological data, including all genera. Genera with sequence data highlighted in bold and connected with thick branches. Fifty percent majority rule consensus tree from 45,000 post-burnin samples. Average standard deviation of split frequencies between two independent runs was 0.003744. Bayesian posterior probabilities <1.00 shown on branches. H, Hyalonematidae; M, Monorhaphididae; P, Pheronematidae. Scale bar, expected number of substitutions/character replacements per site/character. (JPG 152 KB) [file 12983_2017_191_MOESM27_ESM.jpg]

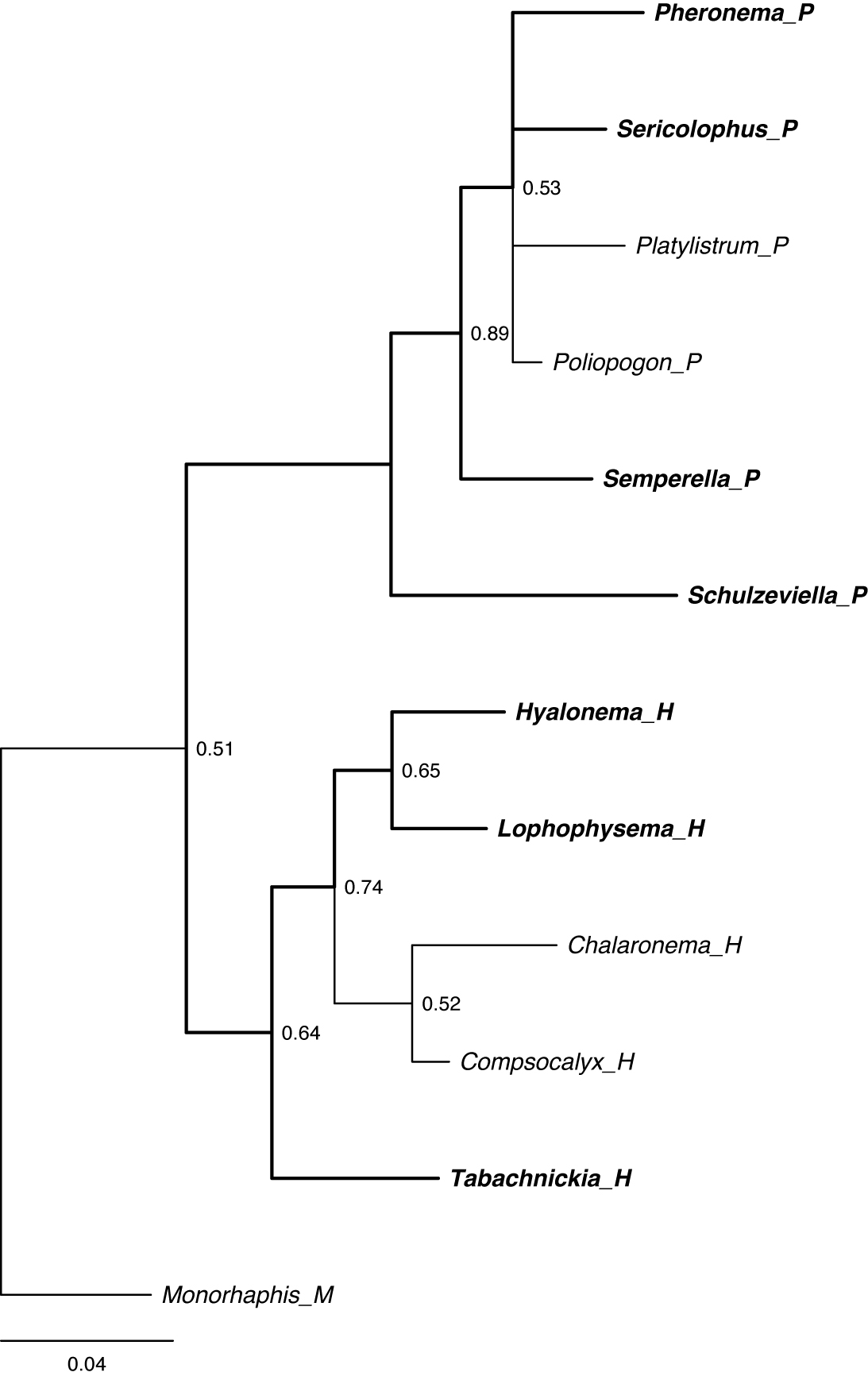

Supplement: Additional file 28: Figure S13. — Phylogeny of Amphidiscophora inferred with RAxML from concatenated molecular and morphological data, including all genera. Genera with sequence data highlighted in bold and connected with thick branches. Bootstrap values >50% shown on branches (based on 1000 pseudoreplicates). H, Hyalonematidae; M, Monorhaphididae; P, Pheronematidae. Scale bar, expected number of substitutions/character replacements per site/character. (JPG 162 KB) [file 12983_2017_191_MOESM28_ESM.jpg]

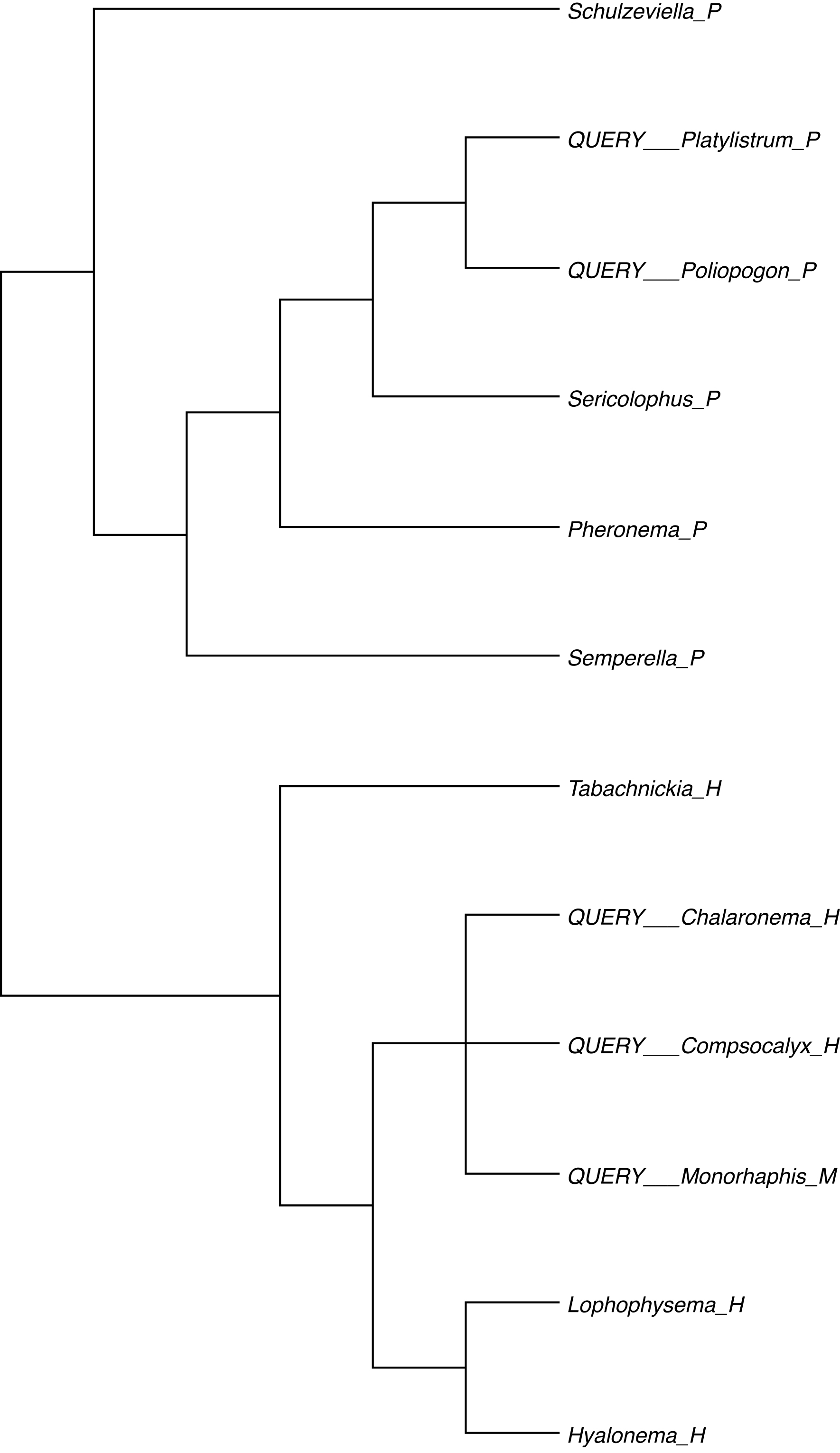

Supplement: Additional file 29: Figure S15. — Placement of Amphidiscophora genera without sequence data (taxon names preceded by “QUERY__”) on the molecular backbone phylogeny using weighted morphology-based phylogenetic binning [22] as implemented in RAxML. H, Hyalonematidae; M, Monorhaphididae; P, Pheronematidae. (JPG 330 KB) [file 12983_2017_191_MOESM29_ESM.jpg]

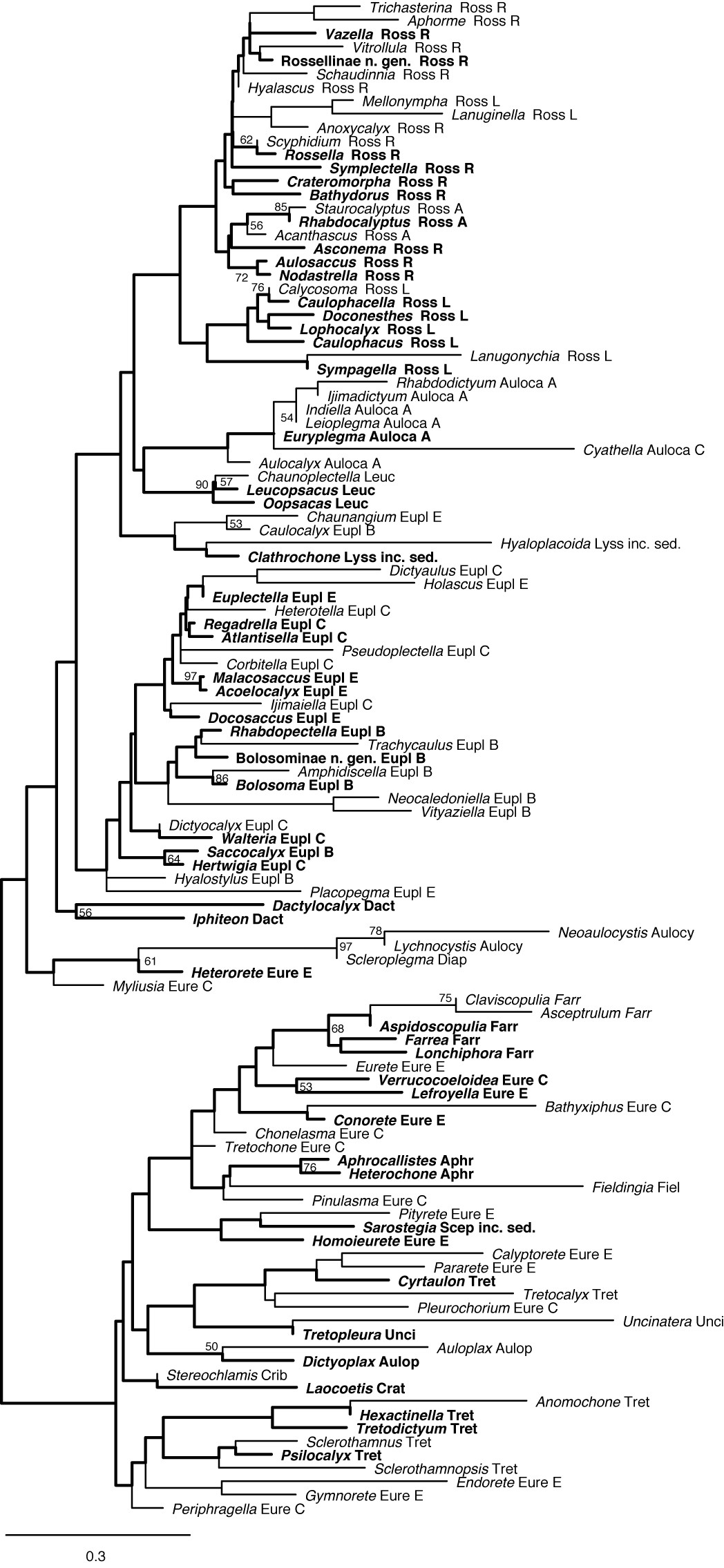

Supplement: Additional file 30: Figure S16. — Phylogeny of Hexasterophora inferred with RAxML from concatenated molecular and morphological data, including all genera. Genera with sequence data highlighted in bold and connected with thick branches. Bootstrap values >50% shown on branches (based on 550 pseudoreplicates). Current family assignment given after genus names: Aphr = Aphrocallistidae, Auloca = Aulocalycidae, Aulocy = Aulocystidae, Aulop = Auloplacidae, Crat = Craticulariidae, Crib = Cribrospongiidae, Dact = Dactylocalycidae, Diap = Diapleuridae, Eupl = Euplectellidae, Eure = Euretidae, Farr = Farreidae, Fiel = Fieldingiidae, Leuc = Leucopsacidae, Lyss inc. sed. = Lyssacinosida incertae sedis, Ross = Rossellidae, Scep inc. sed. = Sceptrulophora incertae sedis, Tret = Tretodictyidae, Unci = Uncinateridae. Subfamilies indicated with letters: A = Acanthascinae (Rossellidae)/Aulocalycinae (Aulocalycidae), B = Bolosominae (Euplectellidae), C = Corbitellinae (Euplectellidae)/ Chonelasmatinae (Euretidae)/Cyathellinae (Aulocalycidae), E = Euplectellinae (Euplectellidae)/Euretinae (Euretidae), L = Lanuginellinae (Rossellidae), R = Rossellinae (Rossellidae). Scale bar, expected number of substitutions/character replacements per site/character. (JPG 648 KB) [file 12983_2017_191_MOESM30_ESM.jpg]

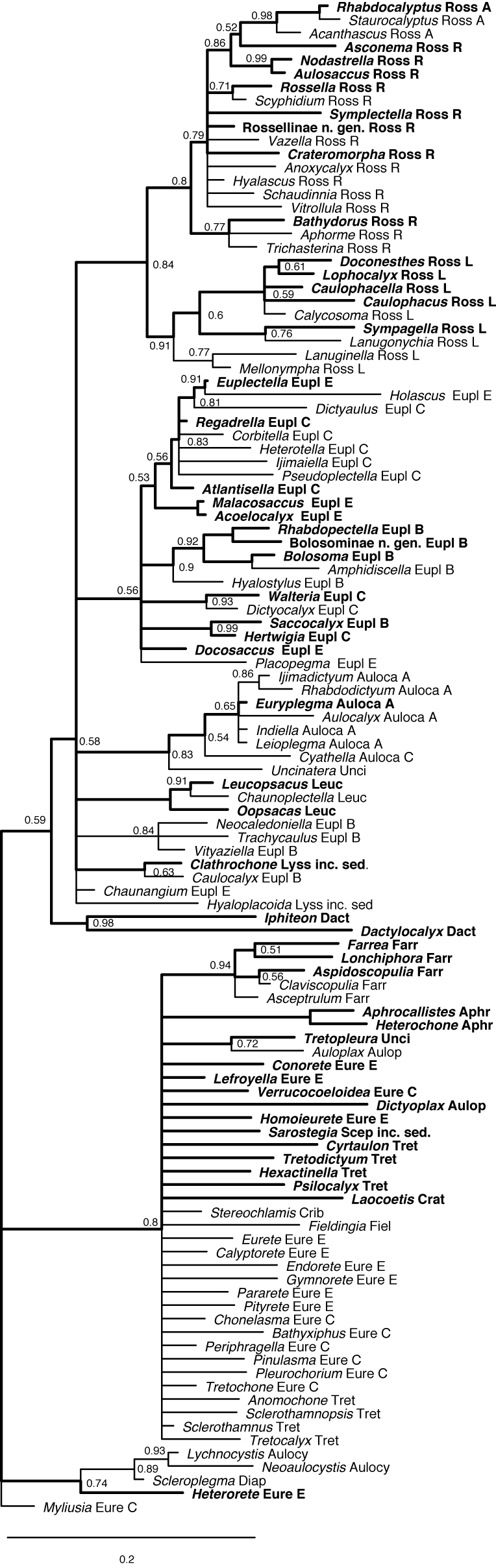

Supplement: Additional file 31: Figure S17. — Phylogeny of Hexasterophora inferred with MrBayes from concatenated molecular and morphological data, including all genera. Genera with sequence data highlighted in bold and connected with thick branches. Fifty percent majority rule consensus tree from 45,000 post-burnin samples. Average standard deviation of split frequencies between two independent runs was 0.043509. Bayesian posterior probabilities <1.00 shown on branches. Current family assignment given after genus names: Aphr = Aphrocallistidae, Auloca = Aulocalycidae, Aulocy = Aulo-cystidae, Aulop = Auloplacidae, Crat = Craticulariidae, Crib = Cribrospongiidae, Dact = Dactylocalycidae, Diap = Diapleuridae, Eupl = Euplectellidae, Eure = Eur- etidae, Farr = Farreidae, Fiel = Fieldingiidae, Leuc = Leucopsacidae, Lyss inc. sed. = Lyssacinosida incertae sedis, Ross = Rossellidae, Scep inc. sed. = Sceptrulo- phora incertae sedis, Tret = Tretodictyidae, Unci = Uncinateridae. Subfamilies in- dicated with letters: A = Acanthascinae (Rossellidae)/Aulocalycinae (Aulocalycidae), B = Bolosominae (Euplectellidae), C = Corbitellinae (Euplectelli- dae)/Chonelasmatinae (Euretidae)/Cyathellinae (Aulocalycidae), E = Euplectelli- nae (Euplectellidae)/Euretinae (Euretidae), L = Lanuginellinae (Rossellidae), R = Rossellinae (Rossellidae). Scale bar, expected number of substitutions/character replacements per site/character. (JPG 637 KB) [file 12983_2017_191_MOESM31_ESM.jpg]

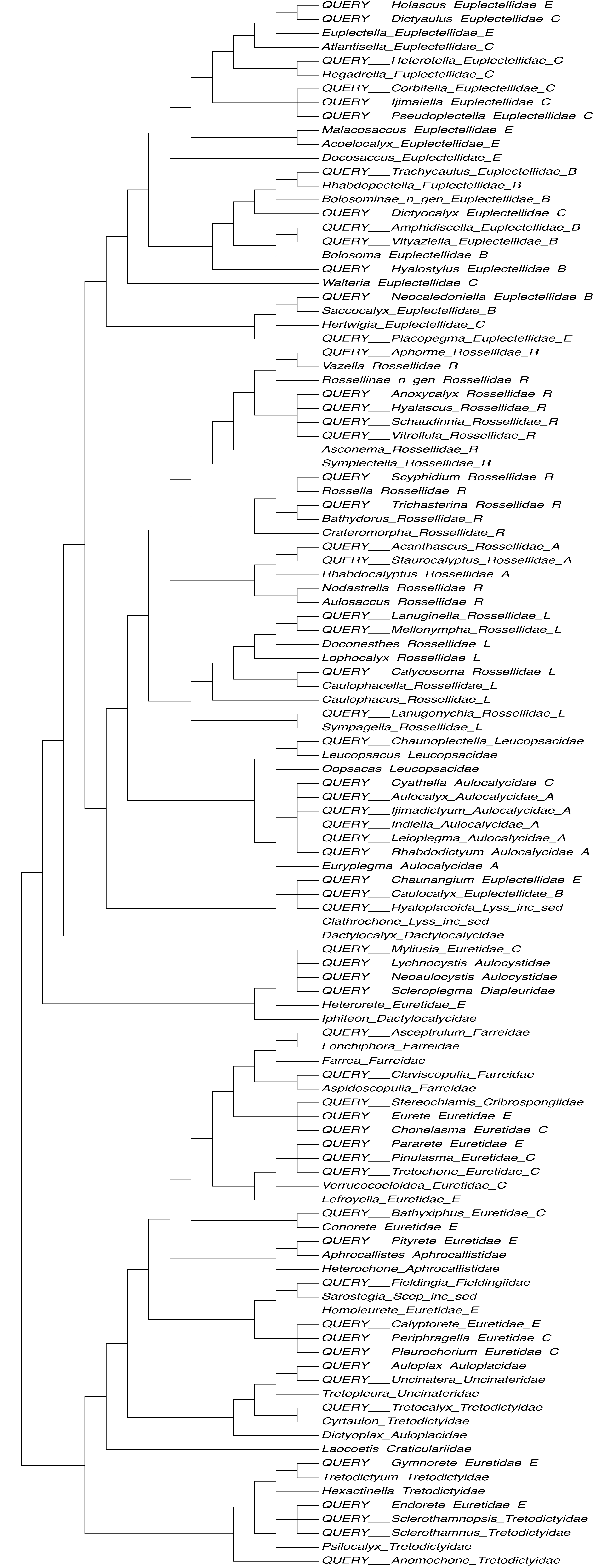

Supplement: Additional file 32: Figure S18. — Placement of Hexasterophora genera without sequence data (taxon names preceded by “QUERY__”) on the molecular backbone phylogeny (Additional file 24: Figure S10) using weighted morphology-based phylogenetic binning [22] as implemented in RAxML. Current family assignment given after genus names. Subfamilies indicated with letters: A = Acanthascinae (Rossellidae)/Aulocalycinae (Aulocalycidae), B = Bolosominae (Euplectellidae), C = Corbitellinae (Euplectellidae)/Chonelasmati- nae (Euretidae)/Cyathellinae (Aulocalycidae), E = Euplectellinae (Euplectellidae)/ Euretinae (Euretidae), L = Lanuginellinae (Rossellidae), R = Rossellinae (Rosselli- dae). (JPG 5.21 MB) [file 12983_2017_191_MOESM32_ESM.jpg]
